# Cross-cultural adaptation, internal consistency, test-retest reliability and feasibility of the German version of the evidence-based practice inventory

**DOI:** 10.1186/s12913-019-4273-0

**Published:** 2019-07-05

**Authors:** Tobias Braun, Katja Ehrenbrusthoff, Carolin Bahns, Lisa Happe, Christian Kopkow

**Affiliations:** 1 Department of Applied Health Sciences, Division of Physiotherapy, Hochschule für Gesundheit (University of Applied Sciences), Gesundheitscampus 6-8, 44801 Bochum, Germany; 2 Department Therapy Science I, Brandenburg Technical University Cottbus – Senftenberg, Universitätsplatz 1, 01968 Senftenberg, Germany

**Keywords:** Evidence-based practice, Evidence-based medicine, Measurement instrument, Reliability, Measurement error, Questionnaire, Cross-sectional survey, Healthcare professional

## Abstract

**Background:**

A psychometrically robust measurement instrument is prerequisite to tailor and monitor interventions aiming to improve evidence-based practice (EBP). The recently developed “Evidence-based Practice Inventory” (EBPI) questionnaire (five dimensions) provides a sound inventory for a comprehensive assessment of adherence to EBP, and identification of barriers and facilitators for EBP. The aims of this study were to establish a German language version of the EBPI and to examine the instrument’s reliability in a diverse sample of healthcare professionals.

**Methods:**

The English version of the EBPI was translated, adopted and subsequently test-retest reliability of the German language EBPI was examined in a nationwide online survey. Participants working in Germany were invited to complete the questionnaire twice.

For each EBPI dimension, internal consistency reliability (Cronbach’s alpha) and the relative test-retest reliability (intraclass correlation coefficient, ICC) were calculated. The standard error of measurement, limits of agreement and minimal detectable change values were estimated to quantify measurement error.

**Results:**

A German language version of the EBPI was established. In the online survey, the EBPI was initially completed by 889 healthcare professionals. At follow-up, 344 individuals (39%) completed the questionnaire (74% female; mean work experience: 13.6 years).

The ICCs for the five dimensions varied between 0.78 and 0.86. The standard error of measurement varied between 6.5 and 8.8% of the respective dimension scale range, and the limits of agreement between 24 and 37%. For internal consistency reliability, alpha varied between 0.64 and 0.90. There were neither floor nor ceiling effects, nor any other relevant feasibility issues.

**Conclusions:**

The German language EBPI can be used to assess EBP adherence of healthcare professionals in clinical practice, and to identify barriers and facilitators for an EBP conform behaviour. Results on test-retest reliability indicate that the EBPI produces reliable scores when used for group comparisons, but the questionnaire seems insufficiently reliable for individual measurements over time. Methods of item response theory or Rasch measurement theory should be used for further evaluation and revision of the EBPI, informed by the results of this study.

**Trial registration:**

German Clinical Trials Register (DRKS00013792). Registered 19 January 2018.

**Electronic supplementary material:**

The online version of this article (10.1186/s12913-019-4273-0) contains supplementary material, which is available to authorized users.

## Background

Evidence-based practice (EBP) is the conscientious, explicit, and judicious use of current best evidence in making decisions about the care of individual patients [1]. EBP is a problem-solving approach providing “a framework for the integration of research evidence and patients’ values and preferences into the delivery of health care” [2, 3]. EBP is an essential competence for clinicians, and the implementation of EBP principles is crucial for improving the quality of delivered healthcare as well as patient outcomes [4].

In general, healthcare professionals have positive attitudes towards EBP and the use of evidence to support clinical decision-making [5–7], perceive EBP as necessary and are interested in incorporating evidence from research into clinical practice [5–9]. However, the implementation of EBP into clinical practice remains challenging, highlighted by a discrepancy between its acceptance and the extent of research use in health care practice [5].

The most frequently reported factors believed to inhibit the use of EBP in clinical practice are time restrictions, limited access to literature as well as poor skills in literature search and critical appraisal of existing literature [5–9]. Major facilitators of EBP implementation reported in the literature include frequent educational sessions, specific additional staff to enable research evidence implementation, support from colleagues, personal motivation, and access to resources [6].

To improve EBP behaviour of healthcare professionals, strategies such as an increased support from the organization and on management levels have been discussed [5]. Another important strategy are educational interventions, such as journal clubs, aimed at facilitating skills and knowledge of EBP [10].

To assess the facilitators and barriers of EBP and to evaluate the effectiveness of intervention strategies for improving EBP performance, a comprehensive measurement instrument is prerequisite [4]. Among content and construct validity, reliability of test scores is an important characteristic. Especially when an instrument is used to detect changes in EBP conform behaviour, the measurement instrument must provide trustworthy test scores over time (change scores). To differentiate real change from measurement error, sound evidence on the extent of the latter must be established. Test-retest reliability (relative reliability) concerns the extent to which scores of respondents who have not changed are the same for repeated measurements over time [11]. A further aspect of reliability is measurement error (absolute reliability), defined as the systematic and random error of a patient’s score, not attributed to true changes in the construct under investigation [11]. Parameters of measurement error are the standard error of measurement, the Bland and Altman limits of agreement, and the minimal detectable change (MDC) [11–13].

A wide range of instruments exists for assessing knowledge and skills in EBP performance [14, 15]. Leung et al. reviewed instruments for measuring evidence-based knowledge, skills and/or attitudes in nursing practice [14]. Among 24 different instruments, the authors identified two promising instruments, including the “Evidence Based Practice Questionnaire” developed by Upton et al. 2006 [16, 17]. Although this instrument demonstrates many good measurement properties, there are concerns about its content and construct validity [14]. However, the instrument has been especially developed for measuring the knowledge, skills and attitudes of nurses towards EBP, indicating a strength for this population, but limiting the instrument’s use with other healthcare professionals. Another recently developed instrument is the ‘Health Sciences-Evidence Based Practice’ (HS-EBP) questionnaire for measuring transprofessional EBP [18, 19]. The HS-EBP demonstrated sufficient measurement properties in a large sample of Spanish health science professionals, but the third dimension (Development of professional practice) presented certain difficulties with its content validity. Thus, the authors proposed that the HS-EBP needs subsequent review [19].

**Fig. 1:**
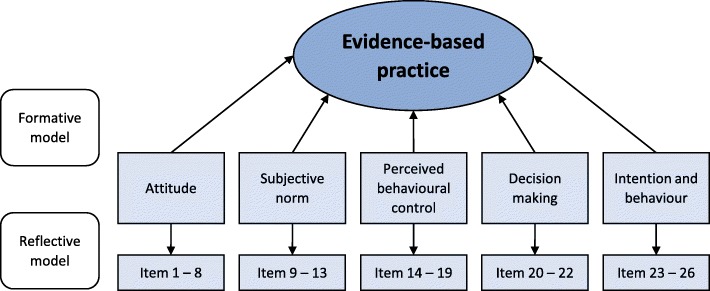
Combined formative and reflective measurement model of the Evidence-based Practice Inventory (EBPI), adopted from Kaper et al. [20]

Another promising instrument is the recently developed “Evidence-based Practice Inventory” (EBPI), an inventory for the comprehensive assessment of EBP adherence and the identification of barriers and facilitators of EBP, through reflection and self-report of clinicians [20]. The EBPI includes crucial domains of EPB, such as competences (knowledge and skills), attitude and behaviour. Furthermore, the instrument aims to address local conditions for EBP in various clinical settings, information processing, and decision making [20]. The English language EBPI, developed in the Netherlands by Kaper et al. [20], is a 26-item questionnaire, including five dimensions (Fig. 1). The EBPI initially proofed to be easy to complete within 15 min and has an established structural validity based on factor analysis [20]. It also demonstrated first evidence of construct validity based on known-groups validity examinations, for sufficient internal consistency reliability for all dimensions but “decision making” (dimension 4), and moderate to substantial test-retest reliability (intraclass correlation coefficient (ICC) between 0.53 and 0.83) [20]. These psychometric properties of the EBPI were examined in a sample of 93 medical doctors, and further reliability analyses in a larger and heterogeneous sample of healthcare professionals seem necessary [11].

A German-language version of the EBPI is deemed valuable to identify facilitators and barriers of EBP and to evaluate implementation strategies of EBP in German speaking countries. Thus, the first objective of this study was the translation and cross-cultural adaptation of the EBPI into German language.

A second objective was to evaluate the internal consistency reliability, the test-retest reliability and the feasibility of the German EBPI, as the psychometric evaluation of a translated measurement instrument in the new linguistic and cultural context is highly recommended [21].

The EBPI was designed to “differentiate in the adherence to EBP among clinicians of different specialties, in various stages of career and vocational training, and with different background and experience in EBP” [20]. However, psychometric testing was performed in an exclusive sample of medical doctors. Thus, the third objective of our study was to evaluate the reliability and the feasibility of the EBPI in a mixed sample and in various uniform sub-samples of healthcare professionals, such as physiotherapists or midwifes.

## Methods

Reporting of this study followed the Strengthening the Reporting of Observational Studies in Epidemiology (STROBE) guideline for observational studies [22] and the Checklist for Reporting Results of Internet E-Surveys (CHERRIES) [23]. Reporting was further informed by the criteria of the COnsensus-based Standards for the selection of health Measurement INstruments (COSMIN) Risk of Bias checklist [24] and the Guidelines for Reporting Reliability and Agreement Studies (GRRAS) [25].

### Design

We performed a prospective cross-sectional study with two phases. In the first phase, the EBPI was translated into German language and adopted for the use in a population of German speaking healthcare professionals according to the ‘translation and cross-cultural adaptation guidelines for self-report measures’ [26]. In a second phase, an online survey was used to assess test-retest reliability and internal consistency of the German EPBI in a sample of healthcare professionals working in Germany.

This study was approved by the Ethical Review Board of the German Confederation for Physiotherapy (registration number: 2017–13). All respondents participated anonymously and voluntarily. By initiating the survey, participants gave informed consent for data analysis and publication. The study was performed according to the ethical principles described in the Declaration of Helsinki and registered a priori in the German Clinical Trials Register (DRKS00013792).

### Evidence-based practice inventory

The EBPI is a questionnaire designed for clinicians to identify barriers and facilitators of EBP [20]. The EPBI consists of 26 items (questions and statements) covering five dimensions: attitude, subjective norm, perceived behavioural control, decision making and intention, and behavior, each rated on a scale from 1 to 6. Usually, items have a positive phrasing for the high-scale extreme and a negative phrasing for the low-scale extreme. For items with two extremes, not necessarily positive or negative, we phrased the item in such a way that it was balanced between one and another extreme. Individual dimensions can be summarized, although summation of all dimensions is not permitted [20]. Items of the originally published EPBI are presented in the table in Additional file 1 and the conceptual design of the EBPI is illustrated in Fig. 1.

### Translation and cross-cultural adaptation

The aim of the translation procedure was to reach conceptual equivalence between the English and the German version of the EBPI. Conceptual equivalence is a prerequisite to maintain content validity across different cultures [26]. Initially, permission to establish a German language version of the EBPI was obtained from the developer of the questionnaire (Nina M. Kaper), who was involved throughout the process of cross-cultural adaptation.

For the German version of the EBPI, the following parts from the original EBPI as published by Kaper et al. [20] were used: Introduction and definitions, dimensions with corresponding definitions and the 26 items from the questionnaire (Additional file 1).

#### Step I: translation

Two native German-speaking translators with fluent English language skills translated the English version of the EBPI into German. One of the translators had a medical background (physiotherapist) and was informed about the concept and the background of the questionnaire. The other translator was uninformed and had no medical background. Each translator worked independently and wrote a report on uncertainties, challenging phrases or alternative phrasing options.

#### Step II: synthesis

The translators discussed their forward translations and produced a synthesis. A written report was used to list issues of dissent and how they were resolved.

#### Step III: back translation

A back translation into English (source language) was performed to ensure that the translated version reflected the same item content as the source version. The German synthesis of the EBPI was independently translated into English by two bilingually raised (British English and German) translators. Both translators were informed (medical students) about the concept and the background of the questionnaire and were blinded regarding the original questionnaire version. Each translator worked independently and wrote a report on uncertainties, challenging phrases or alternative phrasing options.

#### Stage IV: expert committee

Following the translation processes, an expert committee was organized as recommended [26] with the aim to create a pre-final questionnaire version, which could be used for the field-test in stage V. The committee reviewed all translations, all reports and the original English language questionnaire to identify any discrepancies in meaning and suggested modifications to resolve existing discrepancies.

The expert committee consisted of 13 people with different educational and professional backgrounds, including the four translators, two methodologists, an academic language professional and six health professionals (one speech and language therapist, one occupational therapist, three physiotherapists and one physician). The comparison between the original English and the German pre-final version was subjected to semantic, idiomatic, experiential and conceptual equivalence. The complete expert committee meeting was documented in a report written by the principal investigator.

#### Stage V: test of the pre-final version

The pre-final German language EBPI version was field-tested in a convenient sample of healthcare professionals, representing the population in which the questionnaire is intended to be used. A sample size of 30 to 40 individuals is recommended [26]. Participants were recruited via personal address by the study investigators. Inclusion criteria for this piloting sample were: (1) ≥18 years old, (2) graduated healthcare professional, and (3) sufficient German language (spoken and written) to complete the questionnaire. All participants gave written informed consent.

The printed pre-final questionnaire was handed to the participants by research assistants (three graduated physiotherapists). After obtaining socio-demographic data, participants self-completed the EBPI via paper-pencil, followed by a content review (cognitive interviewing). Consultation of the research assistants was prohibited during completion of the questionnaire. The research assistants were well acquainted with the concept of EPB, the process of cross-cultural adaptation of health related outcome measures and the English original questionnaire [20]. Prior to data acquisition, the research assistants participated in a detailed training on the conduction of questionnaires and the technique of ‘cognitive interviewing’ [27].

Based on the concept of ‘cognitive interviewing’, the participants were asked to report their experiences and impressions during the completion of the questionnaire [28]. Four questions were asked: (1) “Was there anything in the introduction, the definitions or the question that you did not understand or did not understand well?” (2) “How confident were you in answering these questions?” (3) “Were there any terms/expressions/formulations that you have never heard before or that were unclear to you?” (4) “How much time did it take to complete the questionnaire?” Participants were encouraged to make further comments on the questionnaire (by verbalizing feelings, impressions, and/or unclearness) in ‘think-aloud-interviews’. These comments, together with all other answers, were recorded in written form.

#### Stage VI: final version and appraisal of the adaptation

All evidence and experiences from the previous stages were used to refine the pre-final questionnaire version. The study team discussed relevant issues, and produced slightly modified translations where necessary. Modifications were reported to the questionnaire developer, and the feedback was used to create a final version of the EPBI. This German language version was submitted to the developer for appraisal of the adaptation process and for final approval.

### Online survey

To analyse the psychometric properties of the final German language version of the EBPI, an online survey was created and set up by using the software “SoSci Survey” (SoSci Survey GmbH, Munich, Germany; https://www.soscisurvey.de). The survey was accessible online only via an internet link to a “survey homepage”. The whole survey was distributed on 14 separate pages/screens (Additional file 2, written in German language): (#1) Invitation letter, including a short description of the background and the aims of the research project, the estimated conduction time of approximately 10 min, and some short instructions on how to complete the survey; (#2) the informed consent formula; (#3) data security information; (#4) notes on how to complete the questionnaire and definitions of the key terms used in the questionnaire, such as “clinician”, “patient” and “evidence”; (#5–7) options to provide socio-demographic data; and (#8–12) the full German language EBPI (one screen for each scale dimension). On a 13th screen, participants were invited to leave an e-mail address if they so wished, which was later used to provide the EBPI a second time (test-retest reliability analysis). A 14th screen finished the survey.

We aimed to provide the definitions given on screen #4 available throughout the online survey. Thus, these terms were always presented in underlined format, and placing the cursor on the term opened a pop-up definition when desired.

Participants could access the survey without any restrictions, such as a password or registration. All the survey’s items were offered in a standardized, unaltered order and participants were able to review and change their answers at any time before completion. In the case of a missing answer, participants were reminded, but not forced to complete missing answers before proceeding to the next screen.

### Participants

The voluntary online survey was accessible to all German-speaking healthcare professionals (unrestricted public internet link and sample of convenience). In this study, we only included data from adult (≥18 years) health professionals (e.g. medicine, midwifery, nursing, occupational therapy, speech and language therapy, sports therapy, psychology, physiotherapy) mainly working in Germany.

We excluded questionnaires from respondents (a) who reported to work < 1 h per week with patients or in clinical care, (b) who aborted the survey before completing the 12th screen and, (c) those who did not complete any of the five dimensions of the EBPI.

### Recruitment procedures and data collection

The online survey was launched for 81 days, starting on 25 January 2018 and ending on 15 April 2018. To recruit healthcare professionals, diverse media and communication channels were used. We created an invitation letter, a press release and a short “advertisement”, all including a description of the study aim and procedure and in addition contact information for the persons responsible of the study. We informed the community of healthcare professionals in Germany about the survey through publication via different media by the institutions, professional societies and journals listed in Additional file 3. No incentives were offered for participation.

Participants who provided an e-mail address for a second participation received an automatically generated invitation e-mail for a second (test-retest) survey 14 days after the first survey completion, including an individualized code to allow for matching of baseline and test-retest data without violating the principle of anonymous participation. A reminder was not sent. Completion of the test-retest survey was possible until 15 May 2018.

To ensure stable/unchanged subject conditions for test-retest reliability analyses [11], a 4-weeks participation period (minimum of 2-weeks and maximum of 6-weeks after first completion) was chosen, considering enough time to avoid recall bias due to memory effects but at the same time unaltered EBP conditions for the participants (facilitators and barriers of EBP).

We asked to distribute the survey link and the project to as many colleagues as possible (“snowball principle”). Per protocol, a total sample size of 800 participants was targeted to allow for sub-group analyses for single healthcare professions with sufficient sample sizes (*n* ≥ 100) [11, 29, 30]. However, we aimed to include as many participants as possible and did not define a maximum sample for the survey.

The number of participants who completed the EBPI a second time was smaller than expected. Thus, most sub-groups (e.g. medicine, nursing) did not reach an “excellent” sample size of 100 participants for reliability analyses, according to recommendations of the COSMIN group [11, 29]. However, deviating from the study protocol, we decided to analyse the reliability of the EBPI of all sub-group with a “good” sample size of ≥50 participants (physiotherapy, occupational therapy, midwifery) [11, 29].

### Statistical analysis

Data from the online survey were saved as an SPSS data file by SoSci Survey. All data were analysed with SPSS Version 25.0 statistical software (SPSS Inc., Chicago, IL, USA) and R Version 3.5.0 (The R Project for Statistical Computing, Vienna, Austria). Descriptive statistics (e.g. mean values with 95% confidence intervals) were used to present sample characteristics.

No weighting of items or propensity scores were used. There was no imputation for missing values. We excluded participants who reported more than one professional background (e.g. nursing and midwifery) from sub-group analyses for single professions.

### Measurement properties

#### Internal consistency reliability

Cronbach’s coefficient alpha, an adequate measure of internal consistency in case of a unidimensional scale, together with appropriate CIs [31], was derived from the baseline sample data because of the large sample size [32]. An alpha value between 0.70 and 0.95 was considered acceptable [32].

#### Test-retest reliability

The relative test-retest reliability for all five dimensions of the EBPI was examined by using the intra-class correlation coefficient model 2.1 (two-way random effects model; ICC_AGREEMENT_) [12]. The ICC_AGREEMENT_ was calculated by dividing the systematic differences between the “true” scores of participants by the error variance, which consists of the systematic differences between the true scores of participants, the variance due to systematic differences between the two measurements, and the residual variance [12].

For group-comparisons, an ICC ≥ 0.7 is deemed acceptable [32, 33]. For individual measurements over time, an ICC ≥0.9 is considered sufficient [32, 33].

#### Measurement error: standard error of measurement

We calculated the standard error of measurement (SEM_AGREEMENT_) for each EBPI dimension. For that we used the same variance components used for the calculation of the ICC_AGREEMENT_. The SEM_AGREEMENT_ was calculated using the square root of the variance between the two questionnaire completions and the error variance of the ICC_AGREEMENT_ [12]. For interpretation, the SEM_AGREEMENT_ was related to the scale range of each dimension.

#### Measurement error: limits of agreement/Bland and Altman plot

To illustrate agreement between the baseline and retest assessment of each EBPI dimension, the method by Bland and Altman was used to calculate the 95% limits of agreement [13]. Homoscedasticity and normally distributed differences are required [34]. Heteroscedasticity was denoted in case of a positive Kendall’s tau (τ) correlation > 0.1 between the absolute differences and the corresponding means was observed [35]. For heteroscedastic data, the following formula was used to calculate the limits of agreement: $-2\mathrm{X}\ \frac{\left({10}^a-1\right)}{\left({10}^a+1\right)} and+2\mathrm{X}\ \frac{\left({10}^a-1\right)}{\left({10}^a+1\right)}$, where *a* = 95% limits of agreement of the 10 log transformed data, and X = the mean score [36]. A corresponding bar charts for frequencies of differences was added to allow better interpretation.

#### Measurement error: minimal detectable change

The minimal detectable change (MDC) is a quantification of absolute agreement. MDC values were calculated based on the test-retest reliability data as MDC_90_ = 1.64*√2*SEM_AGREEMENT_ (MDC with 90 confidence) and MDC_95_ = 1.96*√2*SEM_AGREEMENT_ (MDC with 95% confidence), respectively. The MDC_95_ (MDC_90_) is defined as the minimal amount of change that needs to occur between repeated assessments in an individual to exceed the error of the measurement with 95% (90%) confidence [37].

#### Feasibility

We counted the number of missing values per item in the baseline data of the complete sample and sub-samples by healthcare profession. For items with > 5% of missing values, this item (and the respective dimension) were considered infeasible.

Floor or ceiling effects were assessed by dimension and considered if ≥15% of the participants scored the highest or lowest possible EBPI score [32].

## Results

### Translation process and cross-cultural validation

The translation stages (I to IV) of the EBPI were conducted as planned. During the expert committee stage, some minor and major ambiguities evolved and some terms and items lead to intensive discussions. Detailed results on the stages I to V are described in the Additional file 4, including the characteristics of the pilot sample (*n* = 30) and a list of the major issues evolved during the process of translation and adaptation.

### Online survey

**Fig. 2:**
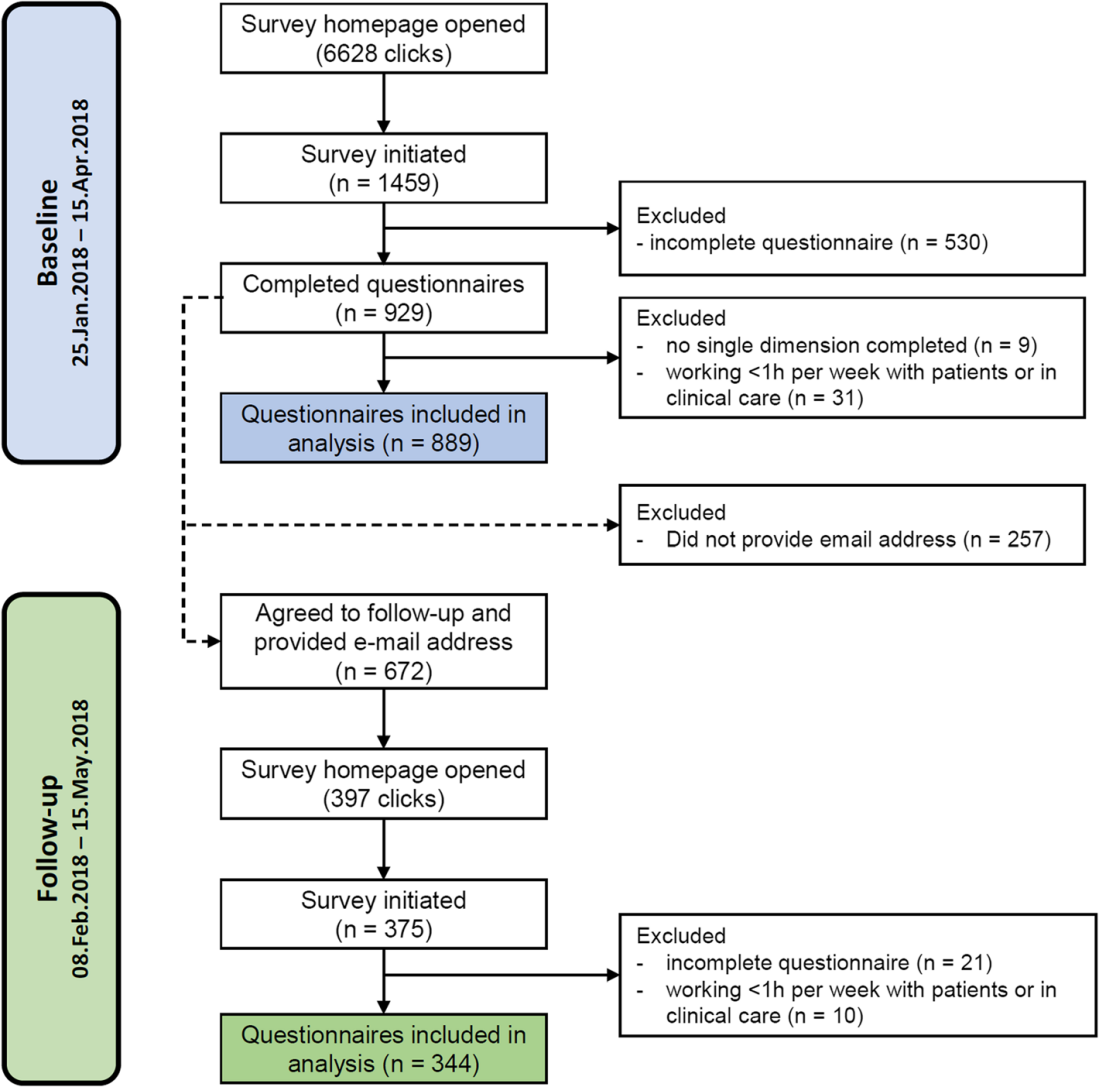
Flow chart of the study

The revised final German language version of the EBPI was included in the online survey (Additional file 2). The total baseline sample included 889 participants (data sets), and 344 participants completed the EBPI twice (follow-up sample), including 130 physiotherapists, 56 occupational therapists and 55 midwifes. A flow chart of the study is provided in Fig. 2. The participants’ characteristics of the baseline samples are given in Table 1. The median time between baseline and follow-up was 14 days (interquartile range: 14–16; mean: 16.4 ± 6.0).

**Table 1:** Socio-demographic characteristics of the participants by sample

Characteristic	Baseline sample (n = 889)	Follow-up sample (*n* = 344)
Age in years, mean	37. 4 ± 11.5 (18–69)	37.9 ± 11.5 (22–69)
Gender: male/female/other, n (%)	225/657/3 (25.4/73.2/0.3) (*n* = 885)	88/253/2 (25.7/73.8/0.6) (*n* = 343)
Individual number of professions: one/two/three, n (%)	838/49/2 (94.3/5.5/0.2) (*n* = 889)	320/22/2 (93.0/6.4/0.6) (n = 344)
Professions^a^, n (%)		
Physiotherapy	318 (35.8)	130 (37.8)
Occupational therapy	154 (17.3)	56 (16.3)
Midwifery	137 (15.4)	55 (16.0)
Speech and language therapy	79 (8.9)	25 (7.3)
Nursing	73 (8.2)	20 (5.8)
Medicine	28 (3.1)	16 (4.7)
Sport therapy/sport sciences	26 (2.9)	9 (2.6)
Psychology	9 (1.0)	2 (0.6)
Other	14 (1.6)	7 (2.0)
≥1 profession	51 (5.7)	24 (7.0)
Highest professional degree, n (%)		
Undergraduate	90 (10.1)	28 (8.1)
Diploma (vocational school)	368 (41.4)	130 (37.8)
Bachelor/diploma (university)	273 (30.7)	108 (31.4)
Master	106 (11.9)	54 (15.7)
Higher academic degree	40 (4.5)	21 (6.1)
Missing	12 (1.3)	3 (0.9)
Contact time with patients, hours per week, mean	26.7 ± 11.8 (1–60) (*n* = 861)	26.1 ± 12.2 (1–55) (*n* = 339)
Primary setting of work, n (%)		
Hospital	196 (22.0)	72 (20.9)
University clinic	58 (6.5)	21 (6.1)
Rehabilitation clinic	61 (6.9)	28(8.1)
Outpatient clinic/private practice	468 (52.6)	186 (54.1)
Other	96 (10.8)	36 (10.5)
Missing	10 (1.1)	1 (0.3)
Work experience in years, mean	13.3 ± 10.8 (0–44) (*n* = 870)	13.6 ± 11.0 (0–42) (*n* = 341)
Employment situation, n (%)		
Employee/worker	584 (65.7)	234 (68.0)
Self-employed	159 (17.9)	59 (17.2)
Freelancer	83 (9.3)	37 (10.8)
Undergraduate/in training/practical year	59 (6.6)	14 (4.1)
Missing	4 (0.4)	0 (0.0)
Leading/leadership position: yes/no, n (%)	286/589 (67.3/32.7) (*n* = 875)	106/231 (31.5/68.5) (*n* = 337)
Having inter-professional communication, n (%)	769 (86.5) (*n* = 884)	299 (87.2) (n = 343)
Inter-professional communication with other professions†, n (%)		
Medicine	679 (88.3)	261 (87.3)
Nursing	461 (59.9)	174 (50.6)
Physiotherapy	454 (59.0)	171 (57.2)
Occupational therapy	345 (44.9)	121 (40.5)
Psychology	297 (38.6)	129 (43.1)
Speech and language therapy	270 (35.1)	93 (31.1)
Sport therapy/sport sciences	146 (19.0)	66 (22.1)
Midwifery	92 (12.0)	45 (13.1)
Other	96 (10.8)	42 (14.0)
Size of the city/municipality of employment, n (%)		
< 5.000 (rural community)	64 (7.2)	23 (6.7)
5.000–20.000 (small town)	153 (17.2)	53 (15.4)
20.000–100.000 (mean sized city)	235 (26.4)	93 (27.0)
> 100.000 (large city)	427 (48.0)	174 (50.6)
Missing	10 (1.1)	1 (0.3)
Available time for scientific literature studies at work within a typical week in minutes per week, mean	53.6 ± 100.8 (0–900) (*n* = 751)	53.3 ± 104.0 (0–900) (*n* = 304)
Availability of scientific literature at work place, n (%)	578 (66.5)	236 (70.0)
Drafting of or involvement in ≥1 scientific publication, n (%)	277 (31.2)	130 (37.8)
Hosting of lectures or workshops on evidence-based practice, n (%)	120 (13.6)	66 (19.3)

Values are the total numbers (percent) or indicated otherwise. Mean values are given with the standard deviation (range). Different sample sized within each sample due to missing values.

^a^ Multiple answers possible

The figures in Additional file 5 (additional results) illustrate the distribution of the respondents across the 16 German federal states for the baseline and the follow-up sample. The figures in Additional file 5 illustrate the baseline and follow-up sample compositions by profession. The characteristics of the follow-up sub-samples by profession are listed in the table in Additional file 5.

### Reliability

#### Internal consistency reliability

**Fig. 3:**
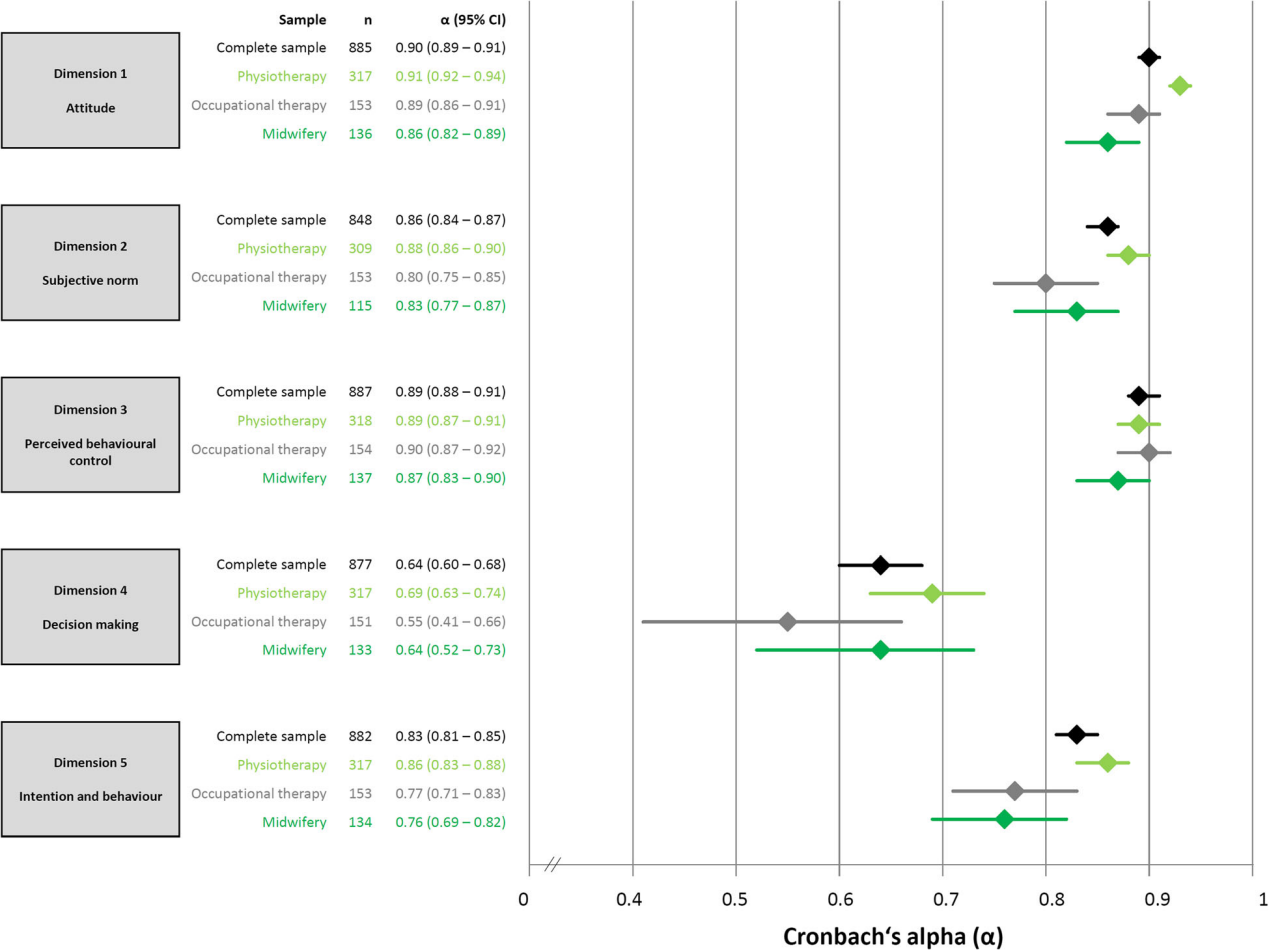
Internal consistency reliability of the Evidence-based Practice Inventory (EBPI)

Cronbach’s alpha values are illustrated in Fig. 3, and varied between the acceptable range (0.70 to 0.95) [32] for dimension 1, 2, 3 and 5, but not for dimension 4 (alpha between 0.55 and 0.69). The same distribution pattern of alpha also applies for the sub-groups physiotherapy, occupational therapy and midwifery.

#### Test-retest reliability

The results of test-retest reliability analysis for the complete follow-up sample are listed in Table 2. Reliability data for the sub-groups of physiotherapists, occupational therapists and midwifes are presented in the tables in the Additional file 5.

**Table 2:** Test-retest reliability of the German language version of the Evidence-based Practice Inventory (EBPI) for the complete sample (n = 344)

Dimension	Dimension 1	Dimension 2	Dimension 3	Dimension 4	Dimension 5
Description of dimension	Attitude	Subjective norm	Perceived behavioural control	Decision making	Intention and behaviour
Items included in dimension	Item 1–8	Item 9–13	Item 14–19	Item 20–22	Item 23–26
Scale range	8–48	5–30	6–36	3–18	4–24
Number completed dimension	343	327	344	342	344
Mean ± SD score 1st measure	39.1 ± 6.4	18.3 ± 5.9	28.1 ± 5.7	13.0 ± 2.7	15.6 ± 4.3
Mean ± SD score 2nd measure	39.3 ± 6.0	19.0 ± 5.7	28.4 ± 5.6	13.2 ± 2.6	15.9 ± 4.2
Mean difference absolute (95% CI)	0.2 (−0.2 to 0.6)	0.7 (0.4 to 1.0)	0.3 (0.0 to 0.7)	0.2 (0.0 to 0.4)	0.3 (0.1 to 0.6)
Mean difference relative to score of 1st measure	0.5%	2.8%	1.0%	1.5%	1.9%
*P* value for mean difference	0.29	< 0.01	0.08	0.03	< 0.01
σ^2^_p_	31.9	29.1	25.8	5.4	15.3
σ^2^_o_	0.0	0.2	0.0	0.0	0.0
σ^2^_residual_	6.8	4.7	6.3	1.5	2.4
ICC_AGREEMENT_	0.82	0.86	0.80	0.78	0.86
95% CI for ICC	0.79 to 0.86	0.82 to 0.88	0.76 to 0.84	0.73 to 0.82	0.83 to 0.89
P value for ICC	< 0.01	< 0.01	< 0.01	< 0.01	< 0.01
SEM_AGREEMENT_ (absolute value)	2.6	2.2	2.5	1.2	1.6
SEM_AGREEMENT_ (relative to scale range)	6.5%	8.8%	8.3%	8.0%	8.0%
τ -correlation ^a^	−0.23	−0.08	−0.27	−0.12	−0.17
Normal distribution of differences ^b^	*p* < 0.01	p < 0.01	p < 0.01	p < 0.01	p < 0.01
95% LoA (log as function of X)	−0.24X + 0.2 to 0.24X + 0.2	0.37X + 0.7 to − 0.37X + 0.7	0.34X + 0.3 to − 0.34X + 0.3	−0.29X + 0.2 to 0.29X + 0.2	0.32X + 0.3 to − 0.32X + 0.3
MDC_90_	6.1	5.2	5.8	2.9	3.6
MDC_95_	7.2	6.2	7.0	3.5	4.3

*SD* Standard deviation, *CI* Confidence interval, σ^2^_p_ Variance between participants, σ^2^_o_ variance due to systematic differences between questionnaire administrations, σ^2^_residual_ Residual variance, *ICC* Intraclass correlation coefficient, *SEM* Standard error of measurement, *LoA* Absolute limits of agreement with 95% confidence, *X* test score, MDC_90_ minimal detectable change with 90% confidence, MDC_95_ Minimal detectable change with 95% confidence

^a^Kendall’s Tau correlation between absolute difference and mean scores of two measures; ^b^ Shapirow Wilk test of Normality

For the complete follow-up sample (*n* = 344), there were statistically significant mean test-retest differences for the dimensions 2, 4 and 5, all below 3% of the baseline scores (Table 2). During follow-up, respondents showed slightly higher scores across all dimensions in comparison to baseline measures. There was no considerable variance due to systematic differences over time in any dimension (σ^2^_o_ between 0 and 0.2).

**Fig. 4:**
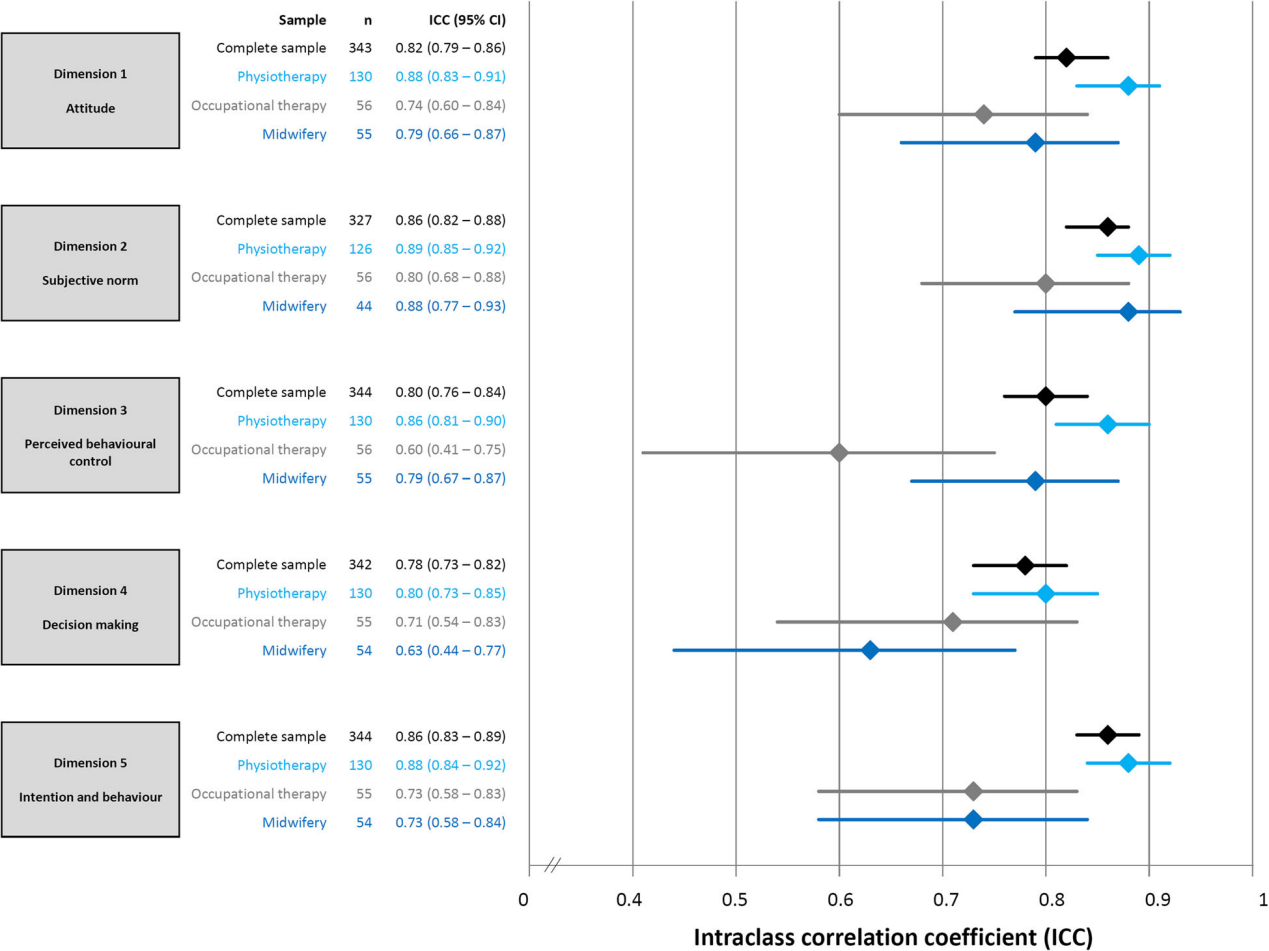
Test-retest reliability of the Evidence-based Practice Inventory (EBPI)

The ICC_AGREEMENT_ of the five dimensions was between 0.78 and 0.86 for the complete sample (Fig. 4). Figure 4 also shows the test-retest reliability of all five dimensions according to sub-groups of physiotherapists, occupational therapists and midwifes. For these sub-groups, the ICC varied between 0.71 and 0.89, except for the dimension 3 for occupational therapists (ICC = 0.60) and dimension 4 for midwifes (ICC = 0.64). However, the upper 95% CI was above the 0.7 cut-off value for those two dimensions.

#### Measurement error: standard error of measurement

SEM_AGREEMENT_ values for all dimension of the complete sample (Table 2) varied between 6.5 and 8.8% of the total scale range of the respective dimension. SEM_AGREEMENT_ values for sub-groups are presented in the tables in Additional file 5.

#### Measurement error: limits of agreement/Bland and Altman plot

**Fig. 5:**
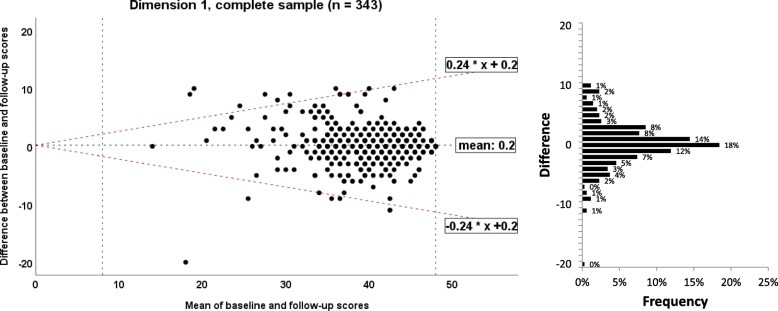
Bland and Altman plots for the dimension 1 of the Evidence-based Practice Inventory (EBPI) for the complete sample

**Fig. 6:**
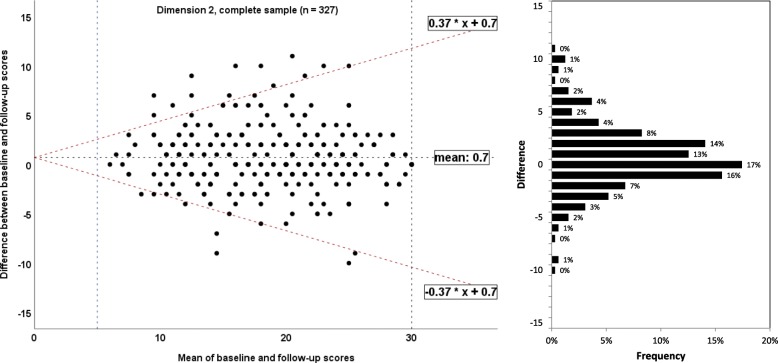
Bland and Altman plots for the dimension 2 of the Evidence-based Practice Inventory (EBPI) for the complete sample

**Fig. 7:**
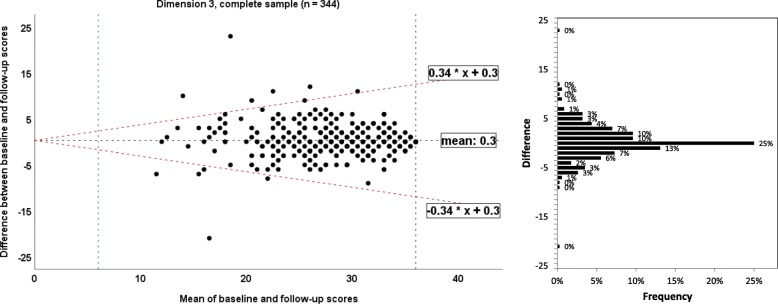
Bland and Altman plots for the dimension 3 of the Evidence-based Practice Inventory (EBPI) for the complete sample

**Fig. 8:**
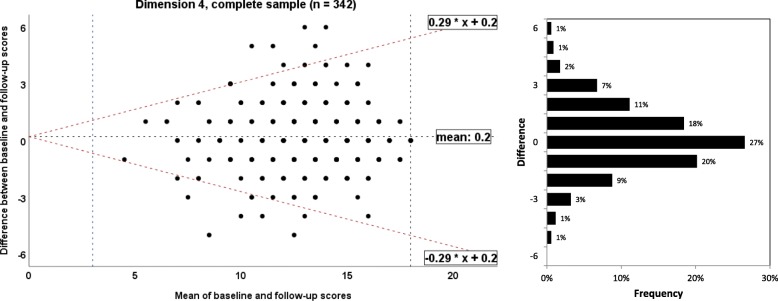
Bland and Altman plots for the dimension 4 of the Evidence-based Practice Inventory (EBPI) for the complete sample

**Fig. 9:**
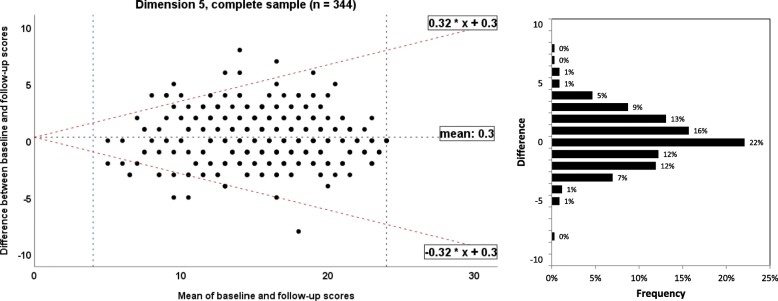
Bland and Altman plots for the dimension 5 of the Evidence-based Practice Inventory (EBPI) for the complete sample

The 95% absolute limits of agreement for the complete sample and sub-samples by profession are listed in Table 2 and in Additional file 5, respectively. Bland and Altman plots for the dimensions 1 to 5 for the complete sample are presented in the Figs. 5, 6, 7, 8 and 9, respectively. The limits of agreement were between 24% (dimension 1) and 37% (dimension 2). Bland and Altman plots for the sub-samples of physiotherapists, occupational therapists and midwifes are given in the Additional file 5.

#### Measurement error: minimal detectable change

MDC_90_ and MDC_95_ values are given in Table 2 (complete sample) and the tables in Additional file 5 (sub-groups by profession).

#### Feasibility

The figures in Additional file 5 illustrate the distribution of response categories and missing values per item for the complete sample and the three sub-samples. The number of missing values per item was < 5%, except for the items #10 (6.6%) and #11 (14.6%) in the sample of midwifes.

The distributions of baseline total scores by dimension and by each sample are shown in the histograms in the Additional file 5. There were no floor or ceiling effects.

## Discussion

This study describes the process of cross-cultural adaptation of the German language EBPI and provides a comprehensive analysis of the internal consistency, test-retest reliability and feasibility of the questionnaire. The three objectives of this study are discussed in the following.

The first objective was the translation and cross-cultural adaptation of the EBPI into German language. A key result is the successful production of a German language EBPI according to recommended, standardized procedures [26]. Based on the results of this study, the German language EBPI can be used to assess EBP adherence of healthcare professionals in clinical practice, and to identify barriers and facilitators of EBP conform behaviour. Furthermore, the EBPI can be used in research and clinical practice to evaluate the impact of efforts of implementing and maximizing EBP [2].

The second objective was to evaluate the internal consistency reliability, the test-retest reliability and the feasibility of the German language EBPI in the new linguistic and cultural context, as recommended [26]. Results indicate adequate internal consistency of the dimensions 1, 2, 3, and 5. Dimension 4 (decision making) shows insufficient internal consistency (α < 0.7). This result is in agreement with the findings reported for the original EBPI, where all dimensions showed good or excellent internal consistency, except for dimension 4 (α = 0.60) [20]. A possible explanation might be the low number of items (*n* = 3) in dimension 4. As stated by Pallant [38], Cronbach's alpha values are quite sensitive to the number of items in the scale, and with short scales (e.g. scales with fewer than ten items) it is common to find quite low Cronbach's alpha values.

The relative test-retest reliability of the 5 dimensions of the German language EBPI for the complete sample of healthcare professionals was sufficient for group-comparisons (ICC ≥ 0.7), but insufficient for individual measurements over time (ICC < 0.9) [32, 33]. However, the comparison between the reliability estimations of the German and the original EBPI is limited since the latter was evaluated in a uniform sample of medical doctors only, and the authors did not report 95% CI for the ICCs. It should further be noted that some of the 95% CI of ICCs found in the present study were not within the “critical” borders of 0.7 and 0.9 (Fig. 3). Thus, the EBPI in this form seems not suitable to measure individual change over a time frame of 14 days based on test-retest reliability estimations.

The interpretation of measurement error is not straight forward, since no clear criteria for an acceptable SEM value are available in the literature. Van Baalen et al. [39] proposed an arbitrary SEM cut-off value of <10% of the total possible range. The relative SEM of the German language EBPI is between 6.5 and 8.8% of the individual dimension scale range, indicating acceptable relative measurement error of the EBPI. In contrast, the 95% limits of agreement are between 24 and 37% for the five dimensions, indicating large (not acceptable) measurement error of the EBPI. No estimations of measurement error for the English language EBPI have been reported yet.

The German EBPI seems feasible in that the administration time is 10 to 15 min and the number of missing values per item is <5%. However, during the process of cross-cultural adaptation, for some items of the EBPI conceptual concerns were noted, resulting in possible lacking comprehensibility and content validity (items #8, #14–19, #25).

The third objective of this study was to evaluate the reliability and the feasibility of the EBPI in various uniform sub-samples of healthcare professionals. We intended to include substantial sub-samples of participants from each major group of healthcare professionals, such as medical doctors, nurses, psychotherapist et cetera. However, the number of participants who completed the EBPI a second time was smaller than expected and thus, participation rates allowed for robust sub-group analyses of physiotherapists (*n* = 130), occupational therapists (*n* = 56) and midwifes (*n* = 55) only. Findings indicate acceptable internal consistency for all five dimensions in all three sub-groups, except for dimension 4.

Since reliability differs substantially between sub-groups, test-retest reliability for sub-groups by profession will be discussed for each single dimension. For dimension 1 (attitude), we found ICC values of 0.74, 0.79 and 0.88 for samples of occupational therapists, midwifes and physiotherapist, respectively. However, the test-retest reliability of dimension 1 of the original EBPI in a sample of medical doctors is substantially lower (ICC = 0.53). For dimension 2 (subjective norm) of the original EBPI, an insufficient ICC of 0.63 is reported [20]. Surprisingly, we found a higher test-retest reliability of 0.80 to 0.89 in sub-samples of allied healthcare professionals for this dimension. Dimension 3 (perceived behavioural control) shows sufficient reliability in medical doctors (ICC = 0.83) [20], physiotherapists (ICC = 0.86) and midwifes (ICC = 0.79), but low reliability (ICC = 0.60) in occupational therapists. Dimension 4 (decision making) and dimension 5 (intention and behaviour) have sufficient test-retest reliability for group-comparisons (ICC ≥ 0.7) in physiotherapists, occupational therapists and medical doctors [20], but dimension 4 seems not sufficiently reliable when it is applied in midwifes (ICC = 0.63). These results indicate that the EBPI produces reliable scores for most dimensions if used for group comparisons in uniform groups of healthcare professionals. However, some dimensions are insufficiently reliable over time if the EBPI is used to quantify EBP performance in uniform groups, such as midwifes or occupational therapist.

The substantial deviations in reliability estimations in some single dimensions, e.g. dimension 1 and 2, may be based on errors in the translation and adaptation process of the German language EBPI, which may have led to disagreement in the content-related equivalence of the English and the German versions. However, we consider distinct amounts of variability between sub-samples by profession to be the reason for diverging reliability estimations between the sub-samples.

Based on the results of this study, the EBPI seems feasible in physiotherapists and occupational therapists, but item #10 and item #11 seem problematic in midwifes. Those two items refer to “my department” and “managers in my department”, respectively. We assume that especially freelancing midwifes and people working in outpatient nursing services did not respond to these items.

### Strengths and limitations

There is no consensus on a method to perform a cross-cultural adaptation of a questionnaire [21]. However, we followed an established guideline [26] and included key elements which are widely recommended (expert committee, target population input, back translation) [21]. Since the committee meeting is an important part of the cross-cultural adaptation process [40], all translators and a relatively large and diverse number of 13 participants with different educational and professional backgrounds collaborated in this meeting. As we strictly followed all recommended steps [26], involved the instrument developer and field-tested the pre-final version in a sample of the target population that was as large as recommended (*n* = 30), we consider the translation and adaptation of the EBPI to be sound and valid.

Sampling bias might be a major limitation of this study. With respect to the online survey, we put much effort into including a representative sample by using different media to inform the community of healthcare professionals in Germany about the survey. We consider it a strength of this study to use the “snowball-principle” and to involve many national professional societies, journals, newspapers, informal groups in social media and other ways to distribute the survey on a national level to as many potential participants as possible. The survey was accessible to all healthcare professionals working in Germany online without any restrictions, such as a password. However, the total number of participants in the baseline (*n* = 889) and the follow-up (*n* = 344) samples were relatively low compared to the total number of healthcare professionals working in Germany. There are, for example, approximately 385,100 active doctors (in 2017) [41], 1,064,342 nurses, midwifes and people working in emergency medical service who are subjected to social insurance contributions (in 2018) [42] and approximately 192,000 physiotherapists (in 2016) [43] working in Germany. Thus, we assume that most healthcare professionals in Germany were not informed about the survey, although we put much effort into a broad distribution. Especially medical doctors and nurses were underrepresented. A more intensive announcement of the survey within these professional groups and the use of the Total Design Method as offered by D.A. Dillman [44] might have increased the participation rate.

A further limitation is that the survey was only accessible online. This might have increased the participation rate of (younger) healthcare professionals and people who were proficient in digital media and online content. But (older) people who are not proficient in online content or people working in institutions without internet access might have been deterred by online administration procedures. The mean age of respondents (37.4 years) was lower than the mean age of the total working population in Germany (43 years) [45]. There are no representative data available for the age distributions of healthcare professionals working in Germany. Younger healthcare professionals may differ from older (more experienced) ones with respect to EPB conform attitudes and behaviour. For example, Dysart et al. [46] reported greater scepticism towards research evidence of more experienced occupational therapists compared to less experienced ones.

Theoretically, multiple baseline questionnaire entries from the same individual were possible since we did not use restrictions, e.g. based on cookies or IP addresses, to assign a unique user identifier to each client computer. However, the test-retest survey was only accessible via a personalized link and survey completion was possible one time only.

Other sources of sampling bias might be the heterogeneous distribution of participants throughout the regional states of Germany and, with respect to the follow-up sub-samples, the overrepresentation of physiotherapists (52%), occupational therapists (46%) and midwifes (45%) with a university-based professional degree in the present samples compared to representative figures. For example, approximately 3% of German physiotherapists have a bachelor degree or higher, respectively [43, 47].

One strength of this study is the sufficiently large [29] sample size of 889 participants for the internal consistency and feasibility analyses. The sample sizes for the test-retest reliability analyses for the complete sample (*n* = 344) and the sample of physiotherapist (*n* = 130) can be judged as “excellent” [11, 29]. However, reliability sample sizes for sub-samples of occupational therapists (*n* = 56) and midwifes (*n* = 55) were smaller and can be judged as “good” [11, 29]. For other common sub-groups, such as medical doctors and nurses, the sample sizes were too small for credible reliability analyses. Further studies are needed to examine the psychometric properties of the EBPI in more balanced samples of healthcare professionals, and in particular, in uniform samples of other caregivers such as nurses, psychologists and medical doctors.

### Implications for further research

The evaluation of other relevant psychometric properties of the German language EBPI was not within the scope of this study, but needs to be analysed further. With respect to the properties proposed by the COSMIN group [11], aspects of content and construct validity seem most important. Before the German EBPI can be used for assessing the effect of interventions to improve EBP behaviour, robust evidence for responsiveness to change of the instrument needs to be established. To allow for interpretation of change scores in the EBPI over time, aspects such as minimal important change values are needed.

The EBPI was published in 2015 [20], and the present study is the second one to evaluate the psychometric quality of the questionnaire. The developers [20] used established methods, including a Delphi study of four rounds with a large international panel of EBP experts to create the EBPI and to achieve sufficient content validity, factor analysis to assess structural validity, and know-group comparisons to assess construct validity in a sufficient-sized group of clinicians [20]. Content validity is the most important measurement property (of a patient-reported outcome measure) [48]. Some authors have argued to stronger acknowledge bioethical values and bioethical principles in EBP, such as respect for autonomy, non-malevolence, beneficience, and justice/equity [49, 50]. These aspects of EBP are neither addressed in the EBPI, nor in other recently published instruments to assess EPB conform behaviour [18, 19] and may limit the content validity of these instruments. To incorporate bioethical values and concepts, such as benefit, harms, costs and justice/equity [49], into a revised version of the EBPI, or a new measurement instrument to assess EPB conform behaviour, these aspects of EBP should be included in initial item-generation and selection (Delphi) processes.

Structural validity refers to the degree to which the scores of a measurement questionnaire are an adequate reflection of the dimensionality of the construct to be measured and is usually assessed by factor analysis or item response theory methods (IRT) or Rasch analysis [11, 51, 52]. The latter methods have not been used to develop the EBPI, but provide a more thorough psychometric evaluation [52, 53]. Thus, we assume further evaluation of structural validity (including unidimensionality), scaling, individual item fit, differential item functioning, and other forms of measurement invariance of the EBPI with methods of IRT or Rasch measurement theory.

## Conclusions

In conclusion, the process of translation and cross-cultural adaptation of the German language EBPI has been completed successfully. The German language EBPI can be used to measure EBP performance of healthcare professionals in Germany and to identify barriers and facilitators of EBP in clinical practice. In general, the feasibility of the German EBPI seems acceptable. However, the reliability issues of the EBPI in both, the English and the German language version, suggest a critical examination and revision of the EBPI, with consideration of minor feasibility issues raised in the present study. Especially dimension 4 (decision making) needs special attention based on its inadequate internal consistency reliability.

Results on the psychometric properties of the EBPI in sub-groups by profession may inform revision of the EBPI. Future examinations should also focus on other groups of healthcare professionals, such as nurses and psychologists, to examine the EBPI in more balanced samples. Methods of IRT or Rasch measurement theory should be used for careful examination and refinement of the EBPI.

To compare the EBPI to other instruments for measuring EBP performance of healthcare professionals, a systematic review following established methods [51] is needed. Since the EBPI and most other available measurement instruments rely on self-report rather than direct measurement of EBP competence [14, 15], an (additional) performance-based instrument for EPB of healthcare professionals is desired.

## Additional files

Additional file 1: Original English language version and final German language version of the “Evidence-based Practice Inventory” (EBPI) (PDF 229 kb)

Additional file 2: Complete online survey (in German language) (PDF 2537 kb)

Additional file 3: Media used to inform the community of healthcare professionals in Germany about the online survey (PDF 69 kb)

Additional file 4: Socio-demographic characteristics of the participants (*n* = 30) in stage V of the process of cross-cultural adaptation (evaluation of the pre-final German language version of the EBPI) (PDF 136 kb)

Additional file 5: Additional results (PDF 1251 kb)

## Data Availability

The datasets used and/or analysed is this study are available from the corresponding author upon reasonable request.

## Abbreviations

CHERRIES: Checklist for Reporting Results of Internet E-Surveys
CI: Confidence interval
COSMIN: COnsensusbased Standards for the selection of health Measurement Instruments
EBP: Evidence-based practice
EBPI: Evidence-based Practice Inventory
GRRAS: Guidelines for Reporting Reliability and Agreement Studies
HS-EBP: Health Sciences-Evidence Based Practice questionnaire
ICC: Intraclass correlation coefficient
IRT: Item response theory
MDC: Minimal detectable change
SEM: Standard error of measurement
STROBE: Strengthening the Reporting of Observational Studies in Epidemiology

## References

[1] Sackett DL, Rosenberg WM, Gray JA, Haynes RB, Richardson WS (1996). Evidence based medicine: what it is and what it isn't. BMJ..

[2] Albarqouni L, Hoffmann T, Straus S, Olsen NR, Young T, Ilic D (2018). Core competencies in evidence-based practice for health professionals. JAMA Netw Open.

[3] Haynes RB, Devereaux PJ, Guyatt GH (2002). Clinical expertise in the era of evidence-based medicine and patient choice. ACP J Club.

[4] Dawes M, Summerskill W, Glasziou P, Cartabellotta A, Martin J, Hopayian K (2005). Sicily statement on evidence-based practice. BMC Med Educ.

[5] Heiwe S, Kajermo KN, Tyni-Lenné R, Guidetti S, Samuelsson M, Andersson I-L, Wengström Y (2011). Evidence-based practice: attitudes, knowledge and behaviour among allied health care professionals. Int J Qual Health Care.

[6] Upton D, Stephens D, Williams B, Scurlock-Evans L (2014). Occupational Therapists' attitudes, knowledge, and implementation of evidence-based practice: a systematic review of published research. Br J Occup Ther.

[7] Smith CA, Coyle ME, de LS, Johnson NP (2014). Evidence-based research and practice: attitudes of reproduction nurses, counsellors and doctors. Reprod BioMed Online.

[8] Young JM, Ward JE (2001). Evidence-based medicine in general practice: beliefs and barriers among Australian GPs. J Eval Clin Pract.

[9] Jette D, Bacon K, Batty C, Carlson M, Ferland A, Hemingway R (2003). Evidence-based practice: beliefs, attitudes, knowledge, and behaviors of physical therapists. Phys Ther.

[10] Young T, Rohwer A, Volmink J, Clarke M (2014). What are the effects of teaching evidence-based health care (EBHC)? Overview of systematic reviews. PLoS One.

[11] Mokkink LB, Terwee CB, Patrick DL, Alonso J, Stratford PW, Knol DL (2010). The COSMIN study reached international consensus on taxonomy, terminology, and definitions of measurement properties for health-related patient-reported outcomes. J Clin Epidemiol.

[12] de Vet HCW, Terwee CB, Mokkink LB, Knol DL (2011). Measurement in medicine: a practical guide.

[13] Bland JM, Altman DG (1986). Statistical methods for assessing agreement between two methods of clinical measurement. Lancet..

[14] Leung K, Trevena L, Waters D (2014). Systematic review of instruments for measuring nurses' knowledge, skills and attitudes for evidence-based practice. J Adv Nurs.

[15] Shaneyfelt T, Baum KD, Bell D, Feldstein D, Houston TK, Kaatz S (2006). Instruments for evaluating education in evidence-based practice: a systematic review. JAMA..

[16] Upton D, Upton P (2006). Development of an evidence-based practice questionnaire for nurses. J Adv Nurs.

[17] Koehn ML, Lehman K (2008). Nurses' perceptions of evidence-based nursing practice. J Adv Nurs.

[18] Fernández-Domínguez JC, Sesé-Abad A, Morales-Asencio JM, Sastre-Fullana P, Pol-Castañeda S, de Pedro-Gómez JE (2016). Content validity of a health science evidence-based practice questionnaire (HS-EBP) with a web-based modified Delphi approach. Int J Qual Health Care.

[19] Fernández-Domínguez JC, de Pedro-Gómez JE, Morales-Asencio JM, Bennasar-Veny M, Sastre-Fullana P, Sesé-Abad A (2017). Health sciences-evidence based practice questionnaire (HS-EBP) for measuring transprofessional evidence-based practice: creation, development and psychometric validation. PLoS One.

[20] Kaper NM, Swennen MHJ, van Wijk AJ, Kalkman CJ, van Rheenen N, van der Graaf Y, van der Heijden GJMG (2015). The "evidence-based practice inventory": reliability and validity was demonstrated for a novel instrument to identify barriers and facilitators for evidence based practice in health care. J Clin Epidemiol.

[21] Epstein J, Santo RM, Guillemin F (2015). A review of guidelines for cross-cultural adaptation of questionnaires could not bring out a consensus. J Clin Epidemiol.

[22] von Elm E, Altman DG, Egger M, Pocock SJ, Gotzsche PC, Vandenbroucke JP (2008). The strengthening the reporting of observational studies in epidemiology (STROBE) statement: guidelines for reporting observational studies. J Clin Epidemiol.

[23] Eysenbach G (2004). Improving the quality of web surveys: the checklist for reporting results of internet E-surveys (CHERRIES). J Med Internet Res.

[24] Mokkink LB, de Vet HCW, Prinsen CAC, Patrick DL, Alonso J, Bouter LM, Terwee CB (2018). COSMIN risk of Bias checklist for systematic reviews of patient-reported outcome measures. Qual Life Res.

[25] Kottner J, Audige L, Brorson S, Donner A, Gajewski BJ, Hrobjartsson A (2011). Guidelines for reporting reliability and agreement studies (GRRAS) were proposed. J Clin Epidemiol.

[26] Beaton DE, Bombardier C, Guillemin F, Ferraz MB (2000). Guidelines for the process of cross-cultural adaptation of self-report measures. Spine..

[27] Pohontsch N, Meyer T (2015). Cognitive interviewing – a tool to develop and validate questionnaires. Rehabilitation (Stuttg).

[28] Willis GB, Artino AR (2013). What do our respondents think We're asking? Using cognitive interviewing to improve medical education surveys. J Grad Med Educ.

[29] Terwee CB, Mokkink LB, Knol DL, Ostelo RWJG, Bouter LM (2012). Vet HCW de. Rating the methodological quality in systematic reviews of studies on measurement properties: a scoring system for the COSMIN checklist. Qual Life Res.

[30] Hobart JC, Cano SJ, Warner TT, Thompson AJ (2012). What sample sizes for reliability and validity studies in neurology?. J Neurol.

[31] Feldt LS, Woodruff DJ, Salih FA (1987). Statistical inference for coefficient alpha. Appl Psychol Meas.

[32] Terwee CB, Bot SDM, de Boer MR, van der Windt DAWM, Knol DL, Dekker J (2007). Quality criteria were proposed for measurement properties of health status questionnaires. J Clin Epidemiol.

[33] Scientific Advisory Committee of the Medical Outcomes Trust (2002). Assessing health status and quality-of-life instruments: attributes and review criteria. Qual Life Res.

[34] Altman DG, Bland JM (1983). Measurement in medicine: the analysis of method comparison studies. The Statistician.

[35] Brehm MA, Scholtes VA, Dallmeijer AJ, Twisk JW, Harlaar J (2012). The importance of addressing heteroscedasticity in the reliability analysis of ratio-scaled variables: an example based on walking energy-cost measurements. Dev Med Child Neurol.

[36] Euser AM, Dekker FW, Le Cessie S (2008). A practical approach to Bland-Altman plots and variation coefficients for log transformed variables. J Clin Epidemiol.

[37] Stratford PW, Binkley JM, Riddle DL (1996). Health status measures: strategies and analytic methods for assessing change scores. Phys Ther.

[38] Pallant J (2011). SPSS survival manual: a step by step guide to data analysis using SPSS.

[39] van Baalen B, Odding E, van Woensel MPC, Roebroeck ME (2006). Reliability and sensitivity to change of measurement instruments used in a traumatic brain injury population. Clin Rehabil.

[40] Epstein J, Osborne RH, Elsworth GR, Beaton DE, Guillemin F (2015). Cross-cultural adaptation of the health education impact questionnaire: experimental study showed expert committee, not back-translation, added value. J Clin Epidemiol.

[41] STATISTA (2018). Gesamtzahl der Ärzte in Deutschland im Zeitraum von 1990 bis 2017 (in 1.000).

[42] STATISTA (2018). Anzahl der sozialversicherungspflichtig beschäftigten Krankenschwestern, −pfleger, Hebammen und Rettungsdienstler in Deutschland von 1999 bis 2018.

[43] PhysioDeutschland, Deutscher Verband für Physiotherapie (ZVK) e.V. Zahlen, Daten, Fakten aus berufsrelevanten Statistiken. 2018. https://www.physio-deutschland.de/fileadmin/data/bund/Dateien_oeffentlich/Beruf_und_Bildung/Zahlen__Daten__Fakten/Zahlen-Daten-Fakten_01.pdf.

[44] Dillman DA. Mail and internet surveys: the tailored design method--2007 update with new internet, visual, and mixed-mode guide. Hoboken: Wiley; 2011.

[45] Statistisches Bundesamt (2017). Zahl der Woche vom 27. Juni 2017: Erwerbstätige sind im Durchschnitt 43 Jahre alt.

[46] Dysart AM, Tomlin GS (2002). Factors related to evidence-based practice among US occupational therapy clinicians. Am J Occup Ther.

[47] Gerst T, Hibbeler B (2012). Gesundheitsfachberufe: Auf dem Weg in die Akademisierung. Deutsches Ärzteblatt.

[48] Terwee CB, Prinsen CAC, Chiarotto A, Westerman MJ, Patrick DL, Alonso J (2018). COSMIN methodology for evaluating the content validity of patient-reported outcome measures: a Delphi study. Qual Life Res.

[49] Watine J (2011). What sort of bioethical values are the evidence-based medicine and the GRADE approaches willing to deal with?. J Med Ethics.

[50] Watine J, Wils J, Augereau C (2014). Clinical practice guidelines: potential misconceptions of the GRADE approach. J Clin Epidemiol.

[51] Prinsen CAC, Mokkink LB, Bouter LM, Alonso J, Patrick DL, de Vet HCW, Terwee CB (2018). COSMIN guideline for systematic reviews of patient-reported outcome measures. Qual Life Res.

[52] Petrillo J, Cano SJ, McLeod LD, Coon CD (2015). Using classical test theory, item response theory, and Rasch measurement theory to evaluate patient-reported outcome measures: a comparison of worked examples. Value Health.

[53] Hobart JC, Cano SJ, Zajicek JP, Thompson AJ (2007). Rating scales as outcome measures for clinical trials in neurology: problems, solutions, and recommendations. Lancet Neurol.

