# Supplementary material for: Cross-cultural adaptation, internal consistency, test-retest reliability and feasibility of the German version of the evidence-based practice inventory
Source: BMC Health Serv Res. 2019 Jul 5;19:455. doi: 10.1186/s12913-019-4273-0 (PMC6612094; doi:10.1186/s12913-019-4273-0)
Supplement: Supplementary file 1 — Original English language version and final German language version of the “Evidence-based Practice Inventory” (EBPI) (PDF 229 kb) [file 12913_2019_4273_MOESM1_ESM.pdf]

Additional file 1: Original English language version and final German language version of the "Evidence-based Practice Inventory" (EBPI)

Original English language version	Final German language version
**Introduction and definitions**	
As a doctor, you make many clinical decisions for your patients each day. To clarify the process of how doctors make their decisions in daily practice, this survey was designed. Answers to the questions below should reflect the way you typically make your decisions. There are no right or wrong answers, we only ask you to provide your personal point of view.	Als Kliniker treffen Sie jeden Tag viele klinische Entscheidungen für Ihre Patienten. Dieser Fragebogen wurde entworfen, um den Prozess zu veranschaulichen, wie Kliniker zu ihren klinischen Entscheidungen in der täglichen Praxis kommen. Die Antworten zu den unten/anschließend gestellten Fragen sollten die Art, in der Sie üblicherweise Entscheidungen treffen, reflektieren. Wir bitten Sie im Rahmen dieses Fragebogens Ihre persönliche Ansicht darzulegen. Es gibt keine richtigen oder falschen Antworten.
Thank you for your participation. Your responses will be treated anonymously and confidentially.	Vielen Dank für Ihre Teilnahme. Ihre Antworten sind anonym und werden vertraulich behandelt.
Before proceeding to the survey, please carefully read our definitions on "clinical decision" and "evidence based practice". These terms will be used in the survey.	Bevor Sie jedoch zur Beantwortung der Fragen weitergehen, bitten wir Sie, die folgenden Definitionen sorgfältig zu lesen. Diese Definitionen sind für das Verständnis und die korrekte Beantwortung der folgenden Fragen wichtig.
**Definitions**	**Definitionen**
Not included in the original version. English translation by the authors.<br>***Clinician***	**Kliniker**

In this questionnaire, this term includes all people working in health care, such as medical doctors, therapists, nurses, psychologists or midwifes	Der Begriff „Kliniker“ schließt in diesem Fragebogen alle im Gesundheitswesen tätigen Personen ein, wie z. B. Ärzte, Therapeuten, Pflegende, Psychologen oder Hebammen.
Not included in the original version. English translation by the authors.<br>***Patient***<br>*The term “patient” includes all individuals receiving a health-related service. This includes healthy individuals too (e.g. expectant mothers, individuals in primary prevention) or individuals who are called “clients” or “customers” by some occupational groups.*	**Patient**<br>Der Begriff „Patient“ umfasst alle Personen, die eine gesundheitsbezogene Dienstleistung in Anspruch nehmen. Dies schließt auch gesunde Personen (z. B. werdende Mütter, Personen in der Primärprävention) oder Personen, die von bestimmten Berufsgruppen als Klienten oder Kunden bezeichnet werden, ein.
Not included in the original version. English translation by the authors.<br>***Evidence***<br>*Within the context of evidence-based medicine, the term “evidence” roots in the English term “evidence” and refers to information from scientific studies and systematically complied experiences, which conform or refute a situation or a fact.*	**Evidenz**<br>Im Kontext der evidenzbasierten Medizin leitet sich der Begriff „Evidenz“ vom englischen Wort "evidence" (= Aussage, Beweis, Ergebnis) ab und bezieht sich auf die Informationen aus wissenschaftlichen Studien und systematisch zusammengetragenen klinischen Erfahrungen, die einen Sachverhalt erhärten oder widerlegen.
**Evidence based practice (EBP)**<br>A problem solving approach used for making clinical decisions that integrates the current best research evidence with clinical experience and individual patients’ characteristics, preferences and values.	**Evidenzbasierte Praxis (EBP)**<br>Problemlösungsansatz zum Treffen klinischer Entscheidungen, unter Einbezug der aktuell besten Forschungsevidenz, klinischer Erfahrung und den Charakteristika, Präferenzen und Werten des/der individuellen Patienten.
**Clinical decision**<br>The choice made on what action to take in patient care after evaluation of information on alternative options.	**Klinische Entscheidung**

	Die Entscheidung darüber, welche Maßnahmen in der Patientenversorgung durchzuführen sind, nachdem Informationen über alternative Optionen abgewogen wurden.
Not included in the original version. English translation by the authors.<br>***Guideline***<br>*Guidelines are systematically developed, scientifically reasoned and clinically oriented decision-making tools. Guidelines aim to support healthcare professionals in making decisions on diagnosis, therapy or related clinical situations.*	 **Leitlinie**<br>Leitlinien sind systematisch entwickelte, wissenschaftlich begründete und praxisorientierte Entscheidungshilfen. Sie sollen in der Gesundheitsbranche tätige Personen dabei unterstützen, Entscheidungen bezüglich Diagnose, Therapie oder verwandten klinischen Sachverhalten zu treffen.
Not included in the original version. English translation by the authors.<br>***Quantitative information***<br>*"Quantitative" means "concerning the quantity", thus, the number, size or amount of something. Quantitative information are information with a quantitative and/or numerical character, expressed e.g. as a number, a sizes or and amount.*	 **Quantitative Informationen**<br>„Quantitativ" bedeutet „die Quantität betreffend", also die Anzahl, Größe oder Menge von etwas. Quantitative Informationen sind Informationen, die einen mengenmäßigen und/oder zahlenmäßigen Charakter haben, also z. B. als Anzahl/Größe/Menge dargestellt sind.
Now please read each question carefully and cross the number of your choice	Lesen Sie nun bitte jede Frage sorgfältig und kreuzen Sie die Zahl Ihrer Wahl an.
Not included in the original version.	Anmerkung**:** Aus Gründen der leichteren Lesbarkeit wird in diesem Fragebogen durchgehend die männliche Sprachform bei personenbezogenen Substantiven und Pronomen verwendet. Dies impliziert jedoch keine Benachteiligung des weiblichen Geschlechts, sondern soll im Sinne der sprachlichen Vereinfachung als geschlechtsneutral zu verstehen sein.
**EBP inventory dimensions and their definitions**	

**Dimension 1 – attitude**<br>Item numbers 1-8	**Dimension 1 – Einstellung**<br>Item 1-8
A clinician's individual evaluation of EBP	Die individuelle EBP Einschätzung eines Klinikers
Overall: ''Do I believe EBP to be good?''<br>Instrumental: ''Does my use of EBP achieve something?''<br>Experiential: ''How does it feel when I apply EBP?''	Insgesamt: „Bin ich der Ansicht, dass EBP gut ist?"<br>Instrumentell: „Bewirkt mein Einsatz von EBP etwas?"<br>Erfahrung: „Wie fühlt es sich an, wenn ich EBP anwende?
**Dimension 2 – subjective norm (including opinion leadership and acceptance of authority)**<br>Item numbers 9-15	**Dimension 2 – subjektive Norm (Einschließlich Meinungsführerschaft und Akzeptanz von Autorität)**<br>Item 9-15
A clinician's own estimate of the social pressure to perform or not to perform EBP behavior	Die eigene Einschätzung eines Klinikers in Bezug auf den sozialen Druck, EBP durchführen oder nicht durchführen zu müssen
Opinion leadership	Meinungsführerschaft
A doctor's expression (and thereby visibility) of his or her opinions and the perceived impact of these opinions on others	Der Ausdruck (und somit die Sichtbarkeit) von Meinungen eines Klinikers und der empfundene Einfluss dieser Meinungen auf Andere
Acceptance of authority	Akzeptanz von Autorität
A doctor's acknowledgment of the recommendations or instructions of others (related to ''motivation to comply'')	Die Bereitschaft eines Klinikers die Empfehlungen oder Anweisungen von Anderen anzuerkennen (bezogen auf die „Motivation sich zu fügen")
Opinions of important individuals or groups (''Do they want me to apply EBP?'')	Meinungen von wichtigen Individuen oder Gruppen („Wollen sie, dass ich EBP anwende?")
Motivation to comply (''Do I think it is important to comply with these opinions?'')	Die Motivation sich zu fügen („Denke ich, dass es wichtig ist, mich diesen Meinungen zu fügen?")

Pressure to comply (‘‘Do I feel pressured to comply with these opinions?’’)	Der Druck, sich zu fügen („Fühle ich mich unter Druck gesetzt, mich diesen Meinungen zu fügen?“)
**Dimension 3 – perceived behavioral control**<br>Item numbers 16-21	**Dimension 3 – empfundene Verhaltenskontrolle**<br>Item 16-21
The extent to which a clinician feels able to enact EBP behavior	Das Ausmaß, in dem sich ein Kliniker in der Lage sieht, EBP auszuführen
Self-efficacy	Selbstwirksamkeit
Knowledge and skills (‘‘Am I able to apply EBP?’’)	Wissen und Fertigkeiten („Bin ich in der Lage, EBP anzuwenden?“)
Self-confidence (‘‘Am I confident that I am able to apply EBP?’’)	Selbstvertrauen („Bin ich zuversichtlich, dass ich EBP anwenden kann?“)
Controllability	Kontrollierbarkeit
Autonomy on performance of behavior (‘‘Is my use of EBP all up to me?’’)	Unabhängigkeit in der Ausführung von EBP („Kann ich selbstbestimmt über den Einsatz von EBP entscheiden?“)
Factors in the clinical setting (‘‘Am I enabled to apply EBP?’’)	Faktoren des klinischen Settings („Werde ich befähigt, EBP anzuwenden?“)
**Dimension 4 – decision making (including information processing and decision making, change potential of behavior; action planning and coping planning)**<br>Item numbers 22-25	**Dimension 4 – Entscheidungsfindung (einschließlich Informationsverarbeitung und Entscheidungsfindung, Änderungspotential von Verhalten; Handlungs- und Bewältigungsplanung)**<br>Item 22-25
The extent to which new information reshapes the clinician’s current understanding and (habitual) behavior	Das Ausmaß, in dem neue Informationen das aktuelle Verständnis und (gewohnheitsmäßige) Verhalten des Klinikers verändern
A clinician’s balance between using intuition or reasoning when handling information to make clinical decisions	Das Gleichgewicht zwischen dem Gebrauch von Intuition und logischem Denken, mit dem ein Kliniker Informationen handhabt, um zu klinischen Entscheidungen zu gelangen

We made parallels between intuition and using clinical experience and between reasoning and using research evidence	Wir zogen Parallelen zwischen Intuition und dem Gebrauch von klinischer Erfahrung sowie zwischen logischem Denken und dem Gebrauch von Evidenz.
Intuition	Intuition
An implicit unconscious process, that is, fast, automatic and based on pattern recognition and habits. This process is very difficult to change or manipulate	Ein indirekter unbewusster Prozess, der schnell und automatisch ist und auf dem Erkennen von Mustern und Verhalten basiert. Dieser Prozess kann nur schwer verändert oder manipuliert werden.
Reasoning	Logisches Denken
An explicit conscious process, that is, slower and controlled. This process is much more volatile, being subject to conscious judgments and attitudes	Ein direkter bewusster Prozess, der langsamer und kontrollierter ist. Dieser Prozess ist viel unbeständiger, da er bewussten Urteilen und Einstellungen ausgesetzt ist.
The clinician's ability to plan, initiate, and maintain intended EBP behavior.	Die Fähigkeit eines Klinikers, beabsichtigte EBP zu planen, einzuleiten und beizubehalten.
Action planning	Handlungsplanung
The specification of when, where, and how to act. Action planning helps to make the leap from behavioral intention to actual behavior	Die genaue Angabe, wann, wo und wie zu handeln ist. Handlungsplanung hilft dabei, den Sprung von der Verhaltensabsicht zum tatsächlichen Verhalten zu machen.
Coping planning	Bewältigungsplanung
Detailed planning of responses to anticipate and cope with personal risk situations that endanger the performance of newly initiated behavior	Detailliertes Planen von Reaktionen um persönliche Risikosituationen, die die Ausführung von neuem Verhalten gefährden, vorauszusehen und zu bewältigen.
**Dimension 5 – intention and behavior**	**Dimension 5 – Absicht und Verhalten**

Item numbers 26-29	Item 26-29
The clinician's aim and actual response, respectively, to apply EBP	Das Ziel, beziehungsweise die Reaktion, eines Klinikers EBP anzuwenden.
Intention: the clinician's aim to enact EBP behavior and his expectation to actually do so	Absicht: das Ziel eines Klinikers, EBP zu veranlassen und seine Erwartung, dies tatsächlich zu tun
Aim (''Am I committed to apply EBP?'')	Ziel („Bin ich entschlossen, EBP anzuwenden?")
Expectation (''Do I expect that I will actually apply EBP?'')	Erwartung („Gehe ich davon aus, EBP tatsächlich anzuwenden?")
Behavior: the clinician's manifest, observable EBP behavior in clinical practice	Verhalten: das offensichtlich erkennbare EBP konforme Verhalten eines Klinikers in der klinischen Praxis.
EBP behavior: doctors who repeatedly compare and adopt their own clinical decisions to latest best standards. For making well-informed decisions, a clinician would need to translate both latest best research evidence and clinical experience to the preferences and clinical needs of individual patients	EBP Verhaltensweise: Kliniker, die wiederholt ihre eigenen klinischen Entscheidungen mit den neuesten Standards vergleichen und diese annehmen. Um gut informierte Entscheidungen zu treffen, müsste ein Kliniker sowohl aktuellste beste Forschungsevidenz als auch klinische Erfahrung auf die Präferenzen und die klinischen Bedürfnisse des individuellen Patienten übertragen.

Evidence-based practice inventory - the questionnaire	
**Attitude**	**Einstellung**
1. I feel that EBP is useless ①②③④⑤⑥ useful to improve my patients' outcomes.	1. Ich halte evidenzbasierte Praxis für nutzlos ①②③④⑤⑥ nützlich, um die Behandlungsergebnisse meiner Patienten zu verbessern.
2. I feel that EBP is an unimportant ①②③④⑤⑥ important feature of high-quality patient care.	2. Ich halte evidenzbasierte Praxis für ein unwichtiges ①②③④⑤⑥ wichtiges Merkmal einer qualitativ hochwertigen Patientenversorgung.
3. I feel that EBP worsens ①②③④⑤⑥ improves the quality of my clinical decisions.	3. Ich denke, evidenzbasierte Praxis verschlechtert ①②③④⑤⑥ verbessert die Qualität meiner klinischen Entscheidungen.
4. I feel that EBP disregards ①②③④⑤⑥ respects my clinical experience.	4. Ich denke, dass evidenzbasierte Praxis meine klinische Erfahrung nicht berücksichtigt ①②③④⑤⑥ berücksichtigt.
5. I feel that EBP disregards ①②③④⑤⑥ respects individual differences between my patients.	5. Ich denke, dass evidenzbasierte Praxis die individuellen Unterschiede meiner Patienten nicht berücksichtigt ①②③④⑤⑥ berücksichtigt.
6. EBP makes me feel constrained ①②③④⑤⑥ autonomous in my clinical decisions.	6. Evidenzbasierte Praxis gibt mir das Gefühl, eingeschränkt ①②③④⑤⑥ uneingeschränkt in meinen klinischen Entscheidungen zu sein.
7. EBP hinders ①②③④⑤⑥ helps me in making better clinical decisions.	7. Evidenzbasierte Praxis hindert mich daran ①②③④⑤⑥ hilft mir, bessere klinische Entscheidungen zu treffen.
8. I feel that clinical guidelines in my own discipline hinder ①②③④⑤⑥ help me in making decisions.	8. Ich denke, dass Leitlinien meiner Disziplin mich daran hindern ①②③④⑤⑥ mir helfen, Entscheidungen zu treffen
**Subjective norm**	**Subjektive Norm**
9. My colleagues discourage ①②③④⑤⑥ encourage me to apply EBP principles in my clinical decisions.	9. Meine Kollegen entmutigen ①②③④⑤⑥ ermutigen mich, Prinzipien der evidenzbasierten Praxis in meinen klinischen Entscheidungen anzuwenden.

10. In my department, we pay no ①②③④⑤⑥ a lot of attention to applying EBP principles in our clinical decisions.	10. In meiner Abteilung wird nicht ①②③④⑤⑥ sehr darauf geachtet, Prinzipien der evidenzbasierten Praxis bei unseren klinischen Entscheidungen anzuwenden.
11. Managers in my department hinder ①②③④⑤⑥ support me to apply EBP principles in my clinical decisions.	11. Die leitenden Personen in meiner Abteilung hindern mich an ①②③④⑤⑥ unterstützen mich in der Anwendung von Prinzipien der evidenzbasierten Praxis in meinen klinischen Entscheidungen.
12. My colleagues and I rarely ①②③④⑤⑥ frequently discuss and challenge how we make our clinical decisions.	12. Meine Kollegen und ich diskutieren und hinterfragen selten ①②③④⑤⑥ häufig, wie wir unsere klinischen Entscheidungen treffen.
13. My colleagues and I rarely ①②③④⑤⑥ frequently discuss research evidence from literature.	13. Meine Kollegen und ich diskutieren selten ①②③④⑤⑥ häufig Evidenz aus der Literatur.
**Perceived behavioral control**	**Empfundene Verhaltenskontrolle**
14. I feel that I am incapable ①②③④⑤⑥ capable of applying EBP principles in my clinical decisions.	14. Ich fühle mich nicht in der Lage ①②③④⑤⑥ in der Lage, Prinzipien der evidenzbasierten Praxis in meinen klinischen Entscheidungen anzuwenden.
15. I feel that I am incapable ①②③④⑤⑥ capable of translating my information needs into relevant and feasible clinical questions.	15. Ich fühle mich nicht in der Lage ①②③④⑤⑥ in der Lage, meinen Informationsbedarf in treffende und plausible klinische Fragen zu übersetzen.
16. I feel that I am incapable ①②③④⑤⑥ capable of searching for research evidence in literature.	16. Ich fühle mich nicht in der Lage ①②③④⑤⑥ in der Lage, nach Evidenz in der Literatur zu suchen.
17. I feel that I am incapable ①②③④⑤⑥ capable of critically appraising research evidence from literature.	17. Ich fühle mich nicht in der Lage ①②③④⑤⑥ in der Lage, Evidenz aus der Literatur kritisch zu bewerten.
18. I feel that I am incapable ①②③④⑤⑥ capable of translating research evidence to the care of my individual patients.	18. Ich fühle mich nicht in der Lage ①②③④⑤⑥ in der Lage, Evidenz auf die Versorgung meiner individuellen Patienten zu übertragen.

19. I feel incapable ①②③④⑤⑥ capable of regularly keeping up with latest research evidence from literature.	19. Ich fühle mich nicht in der Lage ①②③④⑤⑥ in der Lage, mich bezüglich der neuesten Evidenz auf dem Laufenden zu halten.
**Decision making**	**Entscheidungsfindung**
20. I give low ①②③④⑤⑥ high priority to a thorough understanding of the background of the answers to my clinical questions.	20. Bei der Beantwortung klinischer Fragen schreibe ich dem umfassenden Verständnis des Hintergrunds* eine niedrige ①②③④⑤⑥ hohe Priorität zu.<br>* Mit Hintergrund sind alle bekannten und verfügbaren Informationen gemeint, die bei der Beantwortung der klinischen Fragen hilfreich sein können.
21. I dislike ①②③④⑤⑥ like using numbers, tables, and other quantitative information for supporting my clinical decisions.	21. Ich nutze ungern ①②③④⑤⑥ gerne Zahlen, Tabellen und andere quantitative Informationen zur Unterstützung meiner klinischen Entscheidungen.
22. When making clinical decisions, I prefer to use my intuition and experience ①②③④⑤⑥ facts and arguments.	22. Wenn ich klinische Entscheidungen treffe, bevorzuge ich meine Intuition und Erfahrung ①②③④⑤⑥ Fakten und Argumente.
**Intention and behavior**	**Absicht und Verhalten**
23. I rarely ①②③④⑤⑥ frequently use research evidence to support my clinical decisions.	23. Ich nutze selten ①②③④⑤⑥ häufig Evidenz zur Unterstützung meiner klinischen Entscheidungen.
24. I prefer to use my own experience ①②③④⑤⑥ research evidence for making my clinical decisions.	24. Ich bevorzuge meine eigene Erfahrung ①②③④⑤⑥ Forschunsevidenz zum Treffen meiner klinischen Entscheidungen.
25. I tend to ask colleagues ①②③④⑤⑥ search the literature to find answers to my clinical questions.	25. Ich neige dazu, Kollegen zu fragen ①②③④⑤⑥ die Literatur zu durchsuchen, um Antworten auf meine klinischen Fragen zu finden.
26. I rarely ①②③④⑤⑥ frequently seek out available research evidence to answer my daily clinical question.	26. Ich suche selten ①②③④⑤⑥ häufig verfügbare Evidenz um meine täglichen klinischen Fragen zu beantworten.
