# Supplementary material for: Cross-cultural adaptation, internal consistency, test-retest reliability and feasibility of the German version of the evidence-based practice inventory
Source: BMC Health Serv Res. 2019 Jul 5;19:455. doi: 10.1186/s12913-019-4273-0 (PMC6612094; doi:10.1186/s12913-019-4273-0)
Supplement: Supplementary file 2 — Complete online survey (in German language) (PDF 2537 kb) [file 12913_2019_4273_MOESM2_ESM.pdf]

0% ausgefüllt

Deutschlandweite Umfrage zu Förderfaktoren und Barrieren in Bezug auf Evidenzbasierte Praxis

Sehr geehrte Damen und Herren,

wie treffen Personen im Gesundheitswesen in Deutschland Entscheidungen und was beeinflusst diese Entscheidungen?

Die Hochschule für Gesundheit führt in Kooperation mit der Deutschen Gesellschaft für Physiotherapiewissenschaft (DGPTW) e. V. eine Studie zur Untersuchung von Barrieren und Förderfaktoren in der Umsetzung der Evidenzbasierten Praxis durch. Der Begriff Evidenzbasierte Praxis beschreibt einen Problemlösungsansatz zum Treffen klinischer Entscheidungen, unter Einbezug der aktuell besten Forschungsevidenz, klinischer Erfahrung und den Charakteristika, Präferenzen und Werten der/des individuellen Patient/in/en.

Zu diesem Thema führen wir eine deutschlandweite, anonyme und freiwillige Befragung durch. Befragt werden Angehörige verschiedener Gesundheitsprofessionen (z. B. Ergotherapie, Hebammenkunde, Medizin, Pflege, Physiotherapie, Psychologie, Sporttherapie, Sprachtherapie).

Die Ergebnisse können dabei helfen zu verstehen, wie und warum Entscheidungen in der Patientenversorgung getroffen werden. Gleichzeitig kann auf Basis dieser Umfrage möglicherweise ein bestehender Bedarf bezüglich einer Stärkung förderlicher Faktoren sowie des Abbaus existierender Barrieren abgeleitet werden.

Unser Ziel mit dieser Umfrage ist es, einen Beitrag zur Verbesserung der Versorgung von Patientinnen und Patienten zu leisten und wir hoffen, dass Sie ca. 10 Minuten Zeit haben um an dieser Umfrage teilzunehmen.

Bitte nehmen Sie an der Umfrage nur einmal teil, und leiten Sie den Link zur Umfrage bitte auch an Ihre Kolleginnen und Kollegen weiter.

Mit kollegialen Grüßen,

Prof. Dr. Christian Kopkow, MPH	Prof. Dr. Kerstin Lüdtke
Studienleitung	1. Vorsitzende der DGPTW e. V.

Weiter

7% ausgefüllt

Teilnehmerinformation

Was ist das Ziel der Studie?

Ziel der Studie ist die Erfassung von Förderfaktoren und Barrieren in Bezug auf Evidenzbasierte Praxis bei Angehörigen verschiedener Gesundheitsprofessionen (z. B. Ergotherapie, Hebammenkunde, Medizin, Pflege, Physiotherapie, Psychologie, Sporttherapie, Sprachtherapie). Ein weiteres Ziel dieser Studie ist die Prüfung, ob der eingesetzte Fragebogen zuverlässig ist, d.h. ob der Fragebogen zu unterschiedlichen Zeitpunkten das gleiche Ergebnis liefert.

Was wird untersucht?

Um das Studienziel zu erreichen, würden wir Ihnen gerne einige Fragen zu den folgenden Themenbereichen stellen:

Förderfaktoren und Barrieren in der Anwendung von evidenzbasierter Praxis

Demografische Angaben

Wie zeitaufwändig ist die Teilnahme?

Die Teilnahme an der Studie (Online-Befragung) nimmt ca. 10 Minuten in Anspruch.

Sofern Sie einverstanden sind, laden wir Sie 2 Wochen nach erstmaliger Teilnahme ein zweites Mal ein, um an der Befragung teilzunehmen (um die Zuverlässigkeit des Fragebogens zu prüfen). Dies jedoch nur, wenn Sie wollen – gerne können Sie auch nur einmalig an der Befragung teilnehmen.

Was passiert mit meinen Daten?

Die erhobenen Daten werden unter Verschluss gehalten und nur in anonymisierter Form zu wissenschaftlichen Zwecken veröffentlicht. Anonymisieren ist das Verändern personenbezogener Daten derart, dass die Einzelangaben über persönliche oder sachliche Verhältnisse nicht mehr oder nur mit einem unverhältnismäßig großen Aufwand an Zeit, Kosten und Arbeitskraft einer bestimmten oder bestimmbaren natürlichen Person zugeordnet werden können (§ 3 Abs. 6 BDSG). Im Falle einer Veröffentlichung der Studienergebnisse ist eine persönliche Identifikation nicht möglich. Primärdaten als Grundlagen für Veröffentlichungen sollen auf haltbaren und gesicherten Trägern in der Institution, wo sie entstanden sind, für zehn Jahre aufbewahrt werden. Danach werden diese Daten gelöscht, soweit nicht gesetzliche und satzungsmäßige Aufbewahrungsfristen entgegenstehen.

Welche Risiken gibt es?

Die Befragung ist mit keinen Risiken verbunden. Ihre Teilnahme an dieser Studie erfolgt freiwillig. Sie können jederzeit ohne Angabe von Gründen Ihre Teilnahme zurückziehen, ohne dass Ihnen dadurch Nachteile entstehen. Sie sind nicht dazu verpflichtet jede Frage zu beantworten und können die Befragung jederzeit beenden.

Bitte wenden Sie sich an den Studienleiter, Herrn Prof. Dr. Christian Kopkow, falls Sie weitere Fragen zur Studie haben oder etwas an dieser Teilnehmerinformation nicht verstanden haben.

Prof. Dr. Christian Kopkow können Sie erreichen unter:

Telefon: 0234 – 777 27 624

E-Mail: christian.kopkow@hs-gesundheit.de

Vielen Dank für Ihre Teilnahme,

das Studienteam der Hochschule für Gesundheit

Zurück Weiter

14% ausgefüllt

Datenschutzerklärung

- Ich willige ein, dass im Rahmen der Studie erhobene Daten auf elektronischen Datenträgern aufgezeichnet und in anonymisierter* Form bei dem Erhebungs- und Auswertungszentrum** der Studie zur wissenschaftlichen Auswertung gespeichert werden.
- Außerdem willige ich ein, dass ein autorisierter und zur Verschwiegenheit verpflichteter Beauftragter der Ethik-Kommission in meine beim Erhebungs- und Auswertungszentrum** vorhandenen personenbezogenen Daten Einsicht nehmen darf, soweit dies für die Überprüfung der Studie notwendig ist. Für diese Maßnahme entbinde ich das Erhebungs- und Auswertungszentrum von der Schweigepflicht.
- Ich weiß, dass ich meine Zustimmung jederzeit widerrufen kann, ohne dass mir daraus Nachteile entstehen.

* Anonymisieren ist das Verändern personenbezogener Daten derart, dass die Einzelangaben über persönliche oder sachliche Verhältnisse nicht mehr oder nur mit einem unverhältnismäßig großen Aufwand an Zeit, Kosten und Arbeitskraft einer bestimmten oder bestimmbaren natürlichen Person zugeordnet werden können (§ 3 Abs. 6 BDSG).

** Prof. Dr.Christian Kopkow, Hochschule für Gesundheit Bochum, Department für Angewandte Gesundheitswissenschaften, Gesundheitscampus 6-8, 44801 Bochum

Ich habe die Teilnehmerinformation zur Studie „Interkulturelle Adaption und psychometrische Evaluation der deutschen Version des „Evidence-based practice inventory"" gelesen und verstanden. Ich erkläre hiermit meine Einwilligung zur Teilnahme an der genannten Studie und bin mit der Aufzeichnung meiner Daten und deren Weitergabe sowie der Einsichtnahme in meine personenbezogenen Daten in der oben beschriebenen Form einverstanden.

Sie müssen auf **„Weiter"** klicken, um an der Umfrage teilnehmen zu können.

Zurück Weiter

21% ausgefüllt

Wichtiger Hinweis zur Beantwortung der Fragen

Als Kliniker treffen Sie jeden Tag viele klinische Entscheidugen für Ihre Patienten. Dieser Fragebogen wurde entworfen, um den Prozess zu veranschaulichen, wie Kliniker zu ihren klinischen Entscheidungen in der täglichen Praxis kommen. Die Antworten zu den anschließend gestellten Fragen sollten die Art, in der Sie üblicherweise Entscheidungen treffen, reflektieren. Wir bitten Sie im Rahmen dieses Fragebogens Ihre persönliche Ansicht darzulegen. Es gibt keine richtigen oder falschen Antworten.

Bevor Sie jedoch zur Beantwortung der Fragen weitergehen, bitten wir Sie, die folgenden Definitionen sorgfältig zu lesen. Diese Definitionen sind für das Verständnis und die korrekte Beantwortung der folgenden Fragen wichtig.

Anmerkung: Aus Gründen der leichteren Lesbarkeit wird in diesem Fragebogen durchgehend die männliche Sprachform bei personenbezogenen Substantiven und Pronomen verwendet. Dies impliziert jedoch keine Benachteiligung des weiblichen Geschlechts, sondern soll im Sinne der sprachlichen Vereinfachung als geschlechtsneutral zu verstehen sein.

**Kliniker**	Der Begriff „Kliniker“ schließt in diesem Fragebogen alle im Gesundheitswesen tätigen Personen ein, wie z.B. Ärzte, Therapeuten, Pflegende, Psychologen oder Hebammen.
**Patient**	Der Begriff „Patient“ umfasst alle Personen, die eine gesundheitsbezogene Dienstleistung in Anspruch nehmen. Dies schließt auch gesunde Personen (z. B. werdende Mütter, Personen in der Primärprävention) oder Personen, die von bestimmten Berufsgruppen als Klienten oder Kunden bezeichnet werden, ein.
**Evidenz**	Im Kontext der evidenzbasierten Medizin leitet sich der Begriff „Evidenz“ vom englischen Wort "evidence" (= Aussage, Beweis, Ergebnis) ab und bezieht sich auf die Informationen aus wissenschaftlichen Studien und systematisch zusammengetragenen klinischen Erfahrungen, die einen Sachverhalt erhärten oder widerlegen.
**Evidenzbasierte Praxis (EBP)**	Problemlösungsansatz zum Treffen klinischer Entscheidungen, unter Einbezug der aktuell besten Forschungsevidenz, klinischer Erfahrung und den Charakteristika, Präferenzen und Werten des/der individuellen Patienten.
**Klinische Entscheidung**	Die Entscheidung darüber, welche Maßnahmen in der Patientenversorgung durchzuführen sind, nachdem Informationen über alternative Optionen abgewogen wurden.
**Leitlinie**	Leitlinien sind systematisch entwickelte, wissenschaftlich begründete und praxisorientierte Entscheidungshilfen. Sie sollen in der Gesundheitsbranchetätige Personen dabei unterstützen, Entscheidungen bezüglich Diagnose, Therapie oder verwandten klinischen Sachverhalten zu treffen.
**Quantitative Informationen**	„Quantitativ“ bedeutet „die Quantität betreffend“, also die Anzahl, Größe oder Menge von etwas. Quantitative Informationen sind Informationen, die einen mengenmäßigen und/oder zahlenmäßigen Charakter haben, also z. B. als Anzahl/Größe/Menge dargestellt sind.

Hinweis: Fahren Sie im Verlauf des Fragebogens mit dem Mauszeiger über die markierten Wörter, um die Definition erneut anzuzeigen.

Zurück Weiter

Prof. Dr. Christian Kopkow, Department für angewandte Gesundheitswissenschaften, Hochschule für Gesundheit Bochum – 2018

29% ausgefüllt

I. Demografische Angaben

1. Wie alt sind Sie?

- Bitte eingeben Alter
- ☐ keine Angabe

2. Welches Geschlecht haben Sie?

- ○ männlich
- ○ weiblich
- ○ andere
- ○ keine Angabe

3. Welcher Gesundheitsprofession gehören Sie an?
(Mehrfachnennung möglich)

- ☐ Ergotherapie
- ☐ Hebammenkunde
- ☐ Logopädie
- ☐ Medizin
- ☐ Pflege
- ☐ Physiotherapie
- ☐ Psychologie
- ☐ Sportwissenschaften
- ☐ Andere:

4. Welche/n der folgenden Abschlüsse haben Sie bereits im gesundheitlichen Fachbereich erworben?
(Mehrfachnennung möglich)

- ☐ im Studium/in Ausbildung
- ☐ Staatsexamen
- ☐ Diplom
- ☐ Bachelor
- ☐ Master
- ☐ Promotion
- ☐ Andere:
- ☐ keine Angabe

5. Wenn Sie an eine typische Arbeitswoche denken, wie viel Zeit verbringen Sie in direktem Patientenkontakt?

Bitte eingeben Stunden ☐ keine Angabe

6. Was ist Ihr derzeitiges Arbeitsumfeld?

Wenn Sie mehrere Arbeitsplätze haben, dann beantworten Sie bitte diese Frage in Bezug auf den Arbeitsplatz, an welchem Sie am meisten tätig sind.

- ○ Krankenhaus
- ○ Universitätsklinikum
- ○ Rehabilitationseinrichtung
- ○ freie Praxis
- ○ Andere:

- ○ keine Angabe

7. In welchem Bundesland arbeiten Sie?

[Bitte auswählen] ▼

8. Wie viele Jahre an Berufserfahrung haben Sie?

Bitte eingeben Jahre (gerundet auf volle Jahre nach „Berufsausbildung") ☐ keine Angabe

9. Sind Sie:

(Wenn Sie mehrere Arbeitverhältnisse haben, dann beantworten Sie diese Frage bitte in Bezug auf das Arbeitsverhältnis mit dem höchsten Umfang)

- ○ Arbeitnehmer
- ○ selbstständig
- ○ freiberuflich
- ○ in Ausbildung/ im Studium/ im praktischen Jahr oder ähnlichem

- ○ keine Angabe

Zurück Weiter

HSG Hochschule für Gesundheit | DGPTW

36% ausgefüllt

10. Sind Sie in leitender Funktion tätig?

(Definiert als Person mit Personalverantwortung)

- ◯ Ja
- ◯ Nein

- ◯ keine Angabe

11. Befinden Sie sich in Ihrer Einrichtung bzw. Ihrem beruflichen Umfeld/Tätigkeitsfeld im Austausch mit anderen Gesundheitsberufen (z.B. Ärzten, Psychologen, Ergotherapeuten, ...)?

- ◯ Ja
- ◯ Nein

- ◯ keine Angabe

Zurück | Weiter

43% ausgefüllt

12. Wie viele Einwohner leben in der Stadt (oder der Gemeinde), in welcher sich Ihr Arbeitsplatz befindet?

Wenn Sie mehrere Arbeitsplätze haben, dann beantworten Sie diese Frage bitte in Bezug auf den Arbeitsplatz, an welchem Sie die meiste Arbeitszeit tätig sind.

- ◯ <5.000 Einwohner (Landgemeinde)
- ◯ 5.000 - 20.000 Einwohner (Kleinstadt)
- ◯ 20.000 - 100.000 Einwohner (Mittelstadt)
- ◯ >100.000 Einwohner (Großstadt)

- ◯ keine Angabe

13. Wie viel Zeit steht Ihnen in einer typischen Arbeitswoche am Arbeitsplatz zur Verfügung, um sich mit Informationen aus wissenschaftlichen Veröffentlichungen auseinanderzusetzen?

Bitte eingeben Minuten ☐ keine Angabe

14. Haben Sie an Ihrem Arbeitsplatz die Möglichkeit, auf Veröffentlichungen zu evidenzbasierter Praxis zuzugreifen?

- ◯ Ja
- ◯ Nein

- ◯ keine Angabe

15. Haben Sie bereits eine oder mehrere wissenschaftliche Veröffentlichungen geschrieben oder daran mitgearbeitet?

- ◯ Ja
- ◯ Nein

- ◯ keine Angabe

16. Halten Sie Vorträge über, bzw. geben Sie Workshops zum Thema „Evidenzbasierte Praxis"?

- ◯ Ja
- ◯ Nein

- ◯ keine Angabe

Zurück Weiter

hsg Hochschule für Gesundheit   DGPTW

50% ausgefüllt

II. Evidence-based practice inventory

Lesen Sie nun bitte jede Aussage sorgfältig und wählen Sie bei jeder Aussage einen Zahlenwert aus, der am ehesten Ihre Erfahrung/Einstellung widerspiegelt.

Hinweis: Fahren Sie im Verlauf des Fragebogens mit dem Mauszeiger über die markierten Wörter, um die Definition erneut anzuzeigen.

	1 2 3 4 5 6	
1. Ich halte evidenzbasierte Praxis für **nutzlos**	○ ○ ○ ○ ○ ○	**nützlich**, um die Behandlungsergebnisse meiner Patienten zu verbessern.
2. Ich halte evidenzbasierte Praxis für ein **unwichtiges**	○ ○ ○ ○ ○ ○	**wichtiges** Merkmal einer qualitativ hochwertigen Patientenversorgung.
3. Ich denke, evidenzbasierte Praxis **verschlechtert**	○ ○ ○ ○ ○ ○	**verbessert** die Qualität meiner klinischen Entscheidung.
4. Ich denke, dass evidenzbasierte Praxis meine klinische Erfahrung **nicht berücksichtigt**	○ ○ ○ ○ ○ ○	**berücksichtigt.**
5. Ich denke, dass evidenzbasierte Praxis die individuellen Unterschiede meiner Patienten **nicht berücksichtigt**	○ ○ ○ ○ ○ ○	**berücksichtigt**.
6. Evidenzbasierte Praxis gibt mir das Gefühl, **eingeschränkt**	○ ○ ○ ○ ○ ○	**uneingeschränkt** in meinen klinischen Entscheidungen zu sein.
7. Evidenzbasierte Praxis **hindert mich daran**	○ ○ ○ ○ ○ ○	**hilft mir**, bessere klinische Entscheidungen zu treffen.
8. Ich denke, dass Leitlinien meiner Disziplin mich **daran hindern**	○ ○ ○ ○ ○ ○	**mir helfen**, Entscheidungen zu treffen.

Zurück   Weiter

hsg Hochschule für Gesundheit

DGPTW

57% ausgefüllt

Hinweis: Fahren Sie im Verlauf des Fragebogens mit dem Mauszeiger über die markierten Wörter, um die Definition erneut anzuzeigen.

	1 2 3 4 5 6	
9. Meine Kollegen **entmutigen**	○○○○○○	**ermutigen** mich, Prinzipien der evidenzbasierten Praxis in meinen klinischen Entscheidungen anzuwenden.
10. In meiner Abteilung wird **nicht**	○○○○○○	**sehr** darauf geachtet, Prinzipien der evidenzbasierten Praxis bei unseren klinischen Entscheidungen anzuwenden.
11. Die leitenden Personen in meiner Abteilung **hindern mich an**	○○○○○○	**unterstützen** mich in der Anwendung von Prinzipien der evidenzbasierten Praxis in meinen klinischen Entscheidungen.
12. Meine Kollegen und ich diskutieren und hinterfragen **selten**	○○○○○○	**häufig**, wie wir unsere klinischen Entscheidungen treffen.
13. Meine Kollegen und ich diskutieren **selten**	○○○○○○	**häufig** Evidenz aus der Literatur.

Zurück Weiter

64% ausgefüllt

Hinweis: Fahren Sie im Verlauf des Fragebogens mit dem Mauszeiger über die markierten Wörter, um die Definition erneut anzuzeigen.

	1	2	3	4	5	6	
14. Ich fühle mich **nicht in der Lage**	◯	◯	◯	◯	◯	◯	**in der Lage**, Prinzipien der evidenzbasierten Praxis in meinen klinischen Entscheidungen anzuwenden.
15. Ich fühle mich **nicht in der Lage**	◯	◯	◯	◯	◯	◯	**in der Lage**, meinen Informationsbedarf in treffende und plausible klinische Fragen zu übersetzen.
16. Ich fühle mich **nicht in der Lage**	◯	◯	◯	◯	◯	◯	**in der Lage**, nach Evidenz in der Literatur zu suchen.
17. Ich fühle mich **nicht in der Lage**	◯	◯	◯	◯	◯	◯	**in der Lage**, Evidenz aus der Literatur kritisch zu bewerten.
18. Ich fühle mich **nicht in der Lage**	◯	◯	◯	◯	◯	◯	**in der Lage**, Evidenz auf die Versorgung meiner individuellen Patienten zu übertragen.
19. Ich fühle mich **nicht in der Lage**	◯	◯	◯	◯	◯	◯	**in der Lage**, mich bezüglich der neuesten Evidenz auf dem Laufenden zu halten.

Zurück Weiter

71% ausgefüllt

Hinweis: Fahren Sie im Verlauf des Fragebogens mit dem Mauszeiger über die markierten Wörter, um die Definition erneut anzuzeigen.

	1	2	3	4	5	6	
20. Bei der Beantwortung klinischer Fragen schreibe ich dem umfassenden Verständnis des Hintergrunds* eine **niedrige**	◯	◯	◯	◯	◯	◯	**hohe** Priorität zu.
21. Ich nutze **ungern**	◯	◯	◯	◯	◯	◯	**gerne** Zahlen, Tabellen und andere quantitative Informationen zur Unterstützung meiner klinischen Entscheidungen.
22. Wenn ich klinische Entscheidungen treffe, bevorzuge ich meine **Intuition und Erfahrung**	◯	◯	◯	◯	◯	◯	**Fakten und Argumente**.

* Mit Hintergrund sind alle bekannten und verfügbaren Informationen gemeint, die bei der Beantwortung der klinischen Fragen hilfreich sein können.

Zurück | Weiter

79% ausgefüllt

Hinweis: Fahren Sie im Verlauf des Fragebogens mit dem Mauszeiger über die markierten Wörter, um die Definition erneut anzuzeigen.

	1	2	3	4	5	6	
23. Ich nutze **selten**	○	○	○	○	○	○	**häufig** Evidenz zur Unterstützung meiner klinischen Entscheidungen.
24. Ich bevorzuge **meine eigene Erfahrung**	○	○	○	○	○	○	**Forschungsevidenz** zum Treffen meiner klinischen Entscheidungen.
25. Ich neige dazu, **Kollegen zu fragen**	○	○	○	○	○	○	**die Literatur zu durchsuchen**, um Antworten auf meine klinischen Fragen zu finden.
26. Ich suche **selten**	○	○	○	○	○	○	**häufig** verfügbare Evidenz um meine täglichen klinischen Fragen zu beantworten.

Zurück Weiter

86% ausgefüllt

Abschließend bitten wir Sie um Angabe Ihrer Emailadresse, damit wir Sie in 2 Wochen per Email zur Teilnahme an der zweiten Befragung einladen können, um die Zuverlässigkeit des Fragebogens zu prüfen. Ihre Angaben werden selbstverständlich nicht an Dritte weitergegeben und ausschließlich zum Zweck dieser Studie verwendet.

E-Mail-Adresse:

Zurück Weiter

Vielen Dank für Ihre Teilnahme!

Wir möchten uns ganz herzlich für Ihre Mithilfe bedanken.

Ihre Antworten wurden gespeichert, Sie können das Browser-Fenster nun schließen.

Prof. Dr. Christian Kopkow, Department für angewandte Gesundheitswissenschaften, Hochschule für Gesundheit Bochum – 2018
