# Supplementary material for: Cross-cultural adaptation, internal consistency, test-retest reliability and feasibility of the German version of the evidence-based practice inventory
Source: BMC Health Serv Res. 2019 Jul 5;19:455. doi: 10.1186/s12913-019-4273-0 (PMC6612094; doi:10.1186/s12913-019-4273-0)
Supplement: Supplementary file 4 — Socio-demographic characteristics of the participants (n = 30) in stage V of the process of cross-cultural adaptation (evaluation of the pre-final German language version of the EBPI) (PDF 136 kb) [file 12913_2019_4273_MOESM4_ESM.pdf]

Additional file 4: Detailed results for stages I to V of the process of cross-cultural adaptation (evaluation of the pre-final German language version of the EBPI)

The translation stages (I to IV) of the EBPI were conducted as planned. During the expert committee stage, some minor and major ambiguities evolved and some terms and items lead to intensive discussions. The major issues are described in the following:

(1) In the original English language EBPI version, the respondent is usually addressed as “doctor”. We intended to use the EBPI in a broader sample of various healthcare professionals/clinicians. Thus, the committee decided to use the term “Kliniker” (English: “clinician”). “Clinician” includes all healthcare professionals working with patients/clients.

(2) A note was added, indicating that for the sake of better readability, the male pronoun was used throughout the document.

(3) The committee identified some terms that deemed ambiguous or might be unclear to some healthcare professionals: “clinician”, “patient”, “evidence”, “guideline”, “quantitative information”. These terms are not defined in the English language EBPI version, but the committee decided to add definitions of these terms into the introduction section of the German language EBPI version (see Additional file 1). We used standardised definitions of these terms if available. For example, the definition of “evidence” was taken from [1].

All minor and major discrepancies and ambiguities were solved in the committee with additional consultation of the instrument developer, so that the expert committee produced and agreed on a pre-final version of the German language version of the EBPI.

A convenient sample of 30 healthcare professionals completed the pre-final German language EBPI. Eighty percent of participants were female, the mean age was 31.5 years and most participants were

PTs (43%) or nurses (33%), and 77% had a university-based professional degree (socio-demographic characteristics of the participants are summarised in the table below).

**Characteristic**	**Value**
Age in years	31.5 ± 10.4 (22-56)
Gender (male/female), n (%)	6/24 (20/80)
Profession, n (%)	
Medicine	2 (7)
Physiotherapy	13 (43)
Nursing	10 (33)
Occupational therapy	3 (10)
Midwifery	2 (7)
Professional/academic education (highest), n (%)	
Diploma (vocational school)	7 (23)
Bachelor/diploma (university)	17 (57)
Master	3 (10)
Higher academic degree	3 (10)
Setting of work, n (%)	
Hospital	10 (33)
Rehabilitation clinic	4 (13)
Outpatient clinic/private practice	10 (33)
Nursing home/hospice	4 (13)
Other (research institute, nursing service)	2 (7)
Number of patients treated per week (n = 28)	31.4 ± 38.4 (0 – 200)
Work experience in years (n = 28)	7.1 ± 9.2 (0-35)
Number of colleagues in the team/division/department (n = 20)	11.9 ± 5.6 (3-24)
Regular inter-professional communication: yes/no, n (%)	28/2 (93/7)
Number of professions to communicate (n = 27)	3 ± 1.6 (1-6)
Values are mean ± standard deviation (range) or indicated otherwise.	

The 30 participants completed the pre-final German language EBPI in less than 10 minutes and provided feedback on comprehensibility, semantics, concept and layout. Concerning the introduction and definitions, the participants gave valuable recommendations to improve comprehensibility for

clinicians. For example, the order of definitions and wording was finally altered with respect to the pre-final version. Concerning the 26 EBPI items, there were 70 statements (questions, notes, recommendations) in total. Most were of minor concern and lead to slight modifications of single items. For example, the wording of the translation of the items 3 and 4 was changed. Furthermore, there were some conceptual concerns about some items, such as item #8 “I feel that clinical guidelines in my own discipline *hinder/help* me in making decisions.” One respondent noted that guidelines cannot “hinder” in decision making, since guidelines are not mandatory. Items that included the wording “incapable/capable” (items #14-19, e.g. item #19: “I feel *incapable/capable* of regularly keeping up with latest research evidence from literature.”) raised some concerns in that participants argued that they indeed feel capable to perform EBP, but often do not have the time or motivation to do so. Nine participants made a note on item #25 (“I tend to *ask colleagues/search the literature* to find answers to my clinical questions”). The major concern was that this question was hard to answer, that there is no “wrong or right” and that one option does not exclude the other. One participant reported that the character of the clinical question depended which way to go. The committee discussed these issues and decided not to change or exclude any of those items since this would have been a substantial deviation from the English language EBPI version.

Item #20 turned out to be of major concern, because 10 participants described it as too long and/or did not understand the meaning (“I give *low/high* priority to a thorough understanding of the background of the answers to my clinical questions”). Thus, the item was reworded for more clarity. We also added a contextual definition of the term “background”, based on the requested explanation given by the questionnaire developer (Additional file 2).

References Additional file 4:

1. Deutsches Netzwerk Evidenzbasierte Medizin e. V. Was ist EbM. 2017. www.ebm-netzwerk.de. Accessed 11 Dec 2017.
