# Supplementary material for: Cross-cultural adaptation, internal consistency, test-retest reliability and feasibility of the German version of the evidence-based practice inventory
Source: BMC Health Serv Res. 2019 Jul 5;19:455. doi: 10.1186/s12913-019-4273-0 (PMC6612094; doi:10.1186/s12913-019-4273-0)

Additional file 5: Additional results

Figure A1: Distribution of the respondents across the 16 German federal states (baseline sample)

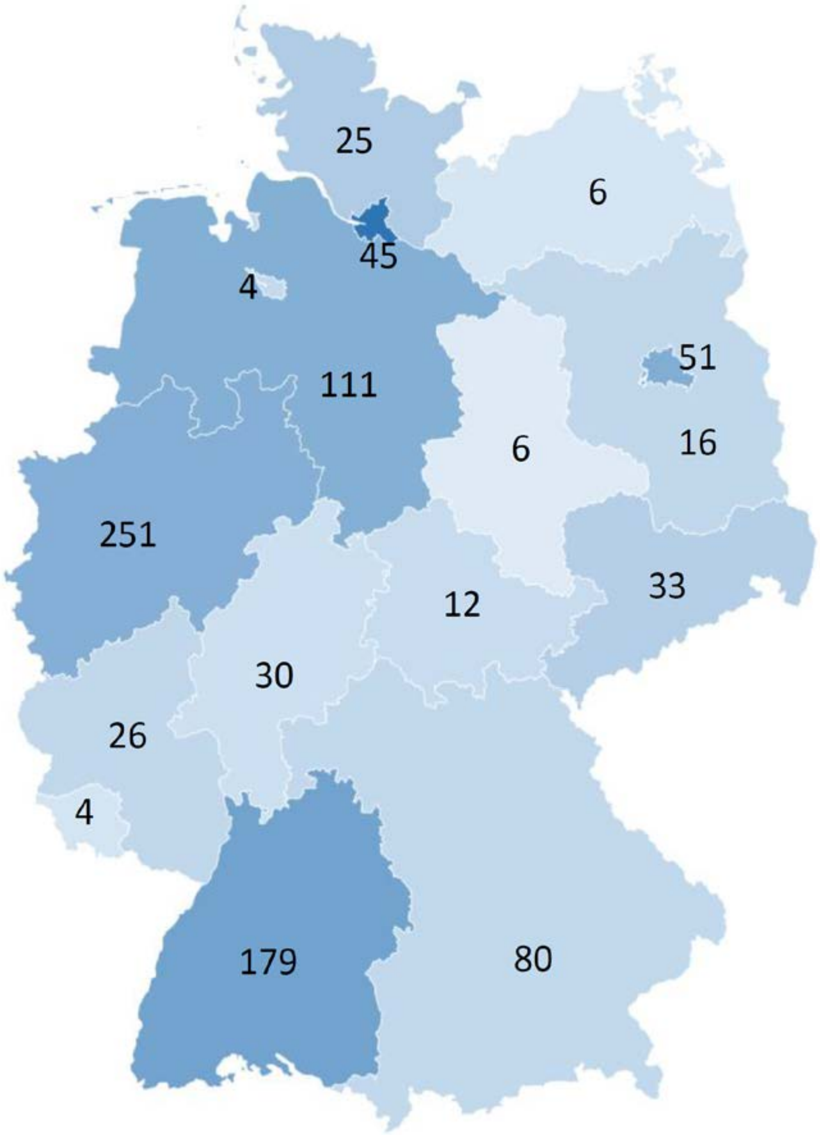

German federal state	Inhabitants	n	Percent
Hamburg	1787408	45	0,00252
Baden-Württemberg	10879618	179	0,00165
Berlin	3520031	51	0,00145
North Rhine-Westphalia (Nordrhein-Westfalen)	17865516	251	0,00140
Lower Saxony (Niedersachsen)	7926599	111	0,00140
Schleswig-Holstein	2858714	25	0,00087
Saxony (Sachsen)	4084851	33	0,00081
Brandenburg	2484826	16	0,00064
Rhineland-Palatinate (Rheinland-Pfalz)	4052803	26	0,00064
Bavaria (Bayern)	12843514	80	0,00062
Bremen	671489	4	0,00060
Thuringia (Thüringen)	2170714	12	0,00055
Hesse (Hessen)	6176172	30	0,00049
Saarland	995597	4	0,00040
Mecklenburg-Vorpommern	1612362	6	0,00037
Saxony-Anhalt (Sachsen-Anhalt)	2245470	6	0,00027

Number of respondents in the baseline sample (n = 879; missing = 10) according to regional states (n = 16) across Germany. Colors indicate the rate between the number of responding physiotherapist and the number of inhabitants in each regional state, with dark and bright blue shades indicating higher and lower rates, respectively. For example, Hamburg has the highest rate (0.00252%) with 45 respondents and 1.787 million inhabitants, and Saxony-Anhalt has the lowest rate (0,00027%) with 6 respondents and 2.245 million inhabitants (Data source: Statistisches Bundesamt; www.destatis.de; “Bundesländer mit Hauptstädten nach Fläche, Bevölkerung und Bevölkerungsdichte am 31.12.2016”).

Figure A2: Distribution of the respondents across the 16 German federal states (follow-up sample)

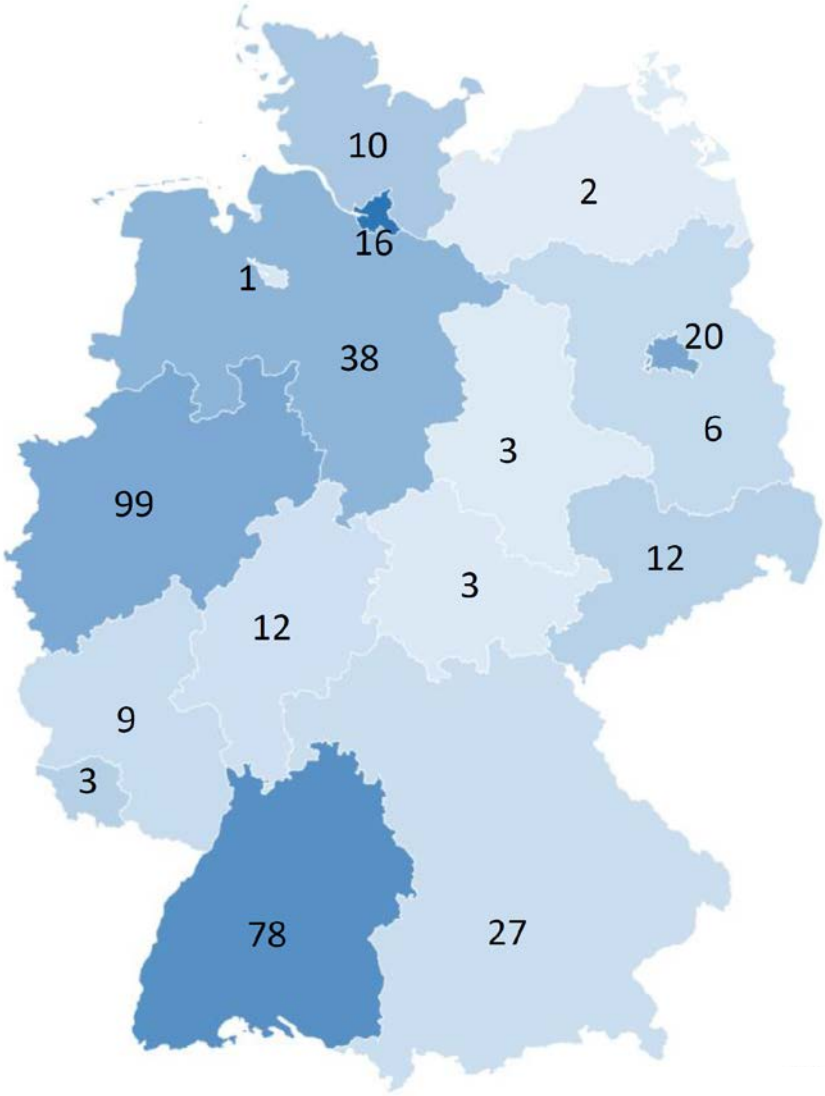

German federal state	Inhabitants	n	Percent
Hamburg	1787408	16	0,00090
Baden-Württemberg	10879618	78	0,00072
Berlin	3520031	20	0,00057
North Rhine-Westphalia (Nordrhein-Westfalen)	17865516	99	0,00055
Lower Saxony (Niedersachsen)	7926599	38	0,00048
Schleswig-Holstein	2858714	10	0,00035
Saarland	995597	3	0,00030
Saxony (Sachsen)	4084851	12	0,00029
Brandenburg	2484826	6	0,00024
Rhineland-Palatinate (Rheinland-Pfalz)	4052803	9	0,00022
Bavaria (Bayern)	12843514	27	0,00021
Hesse (Hessen)	6176172	12	0,00019
Bremen	671489	1	0,00015
Thuringia (Thüringen)	2170714	3	0,00014
Saxony-Anhalt (Sachsen-Anhalt)	2245470	3	0,00013
Mecklenburg-Vorpommern	1612362	2	0,00012

Number of respondents in the follow-up sample (n = 339; missing = 5) according to regional states (n = 16) across Germany. Colors indicate the rate between the number of responding physiotherapist and the number of inhabitants in each regional state, with dark and bright blue shades indicating higher and lower rates, respectively. For example, Hamburg has the highest rate (0.00090%) with 16 respondents and 1.787 million inhabitants, and Mecklenburg-Vorpommern has the lowest rate (0,00012%) with 2 respondents and 1.612 million inhabitants (Data source: Statistisches Bundesamt; www.destatis.de; "Bundesländer mit Hauptstädten nach Fläche, Bevölkerung und Bevölkerungsdichte am 31.12.2016").

Figure B1: Baseline sample composition (n = 889)

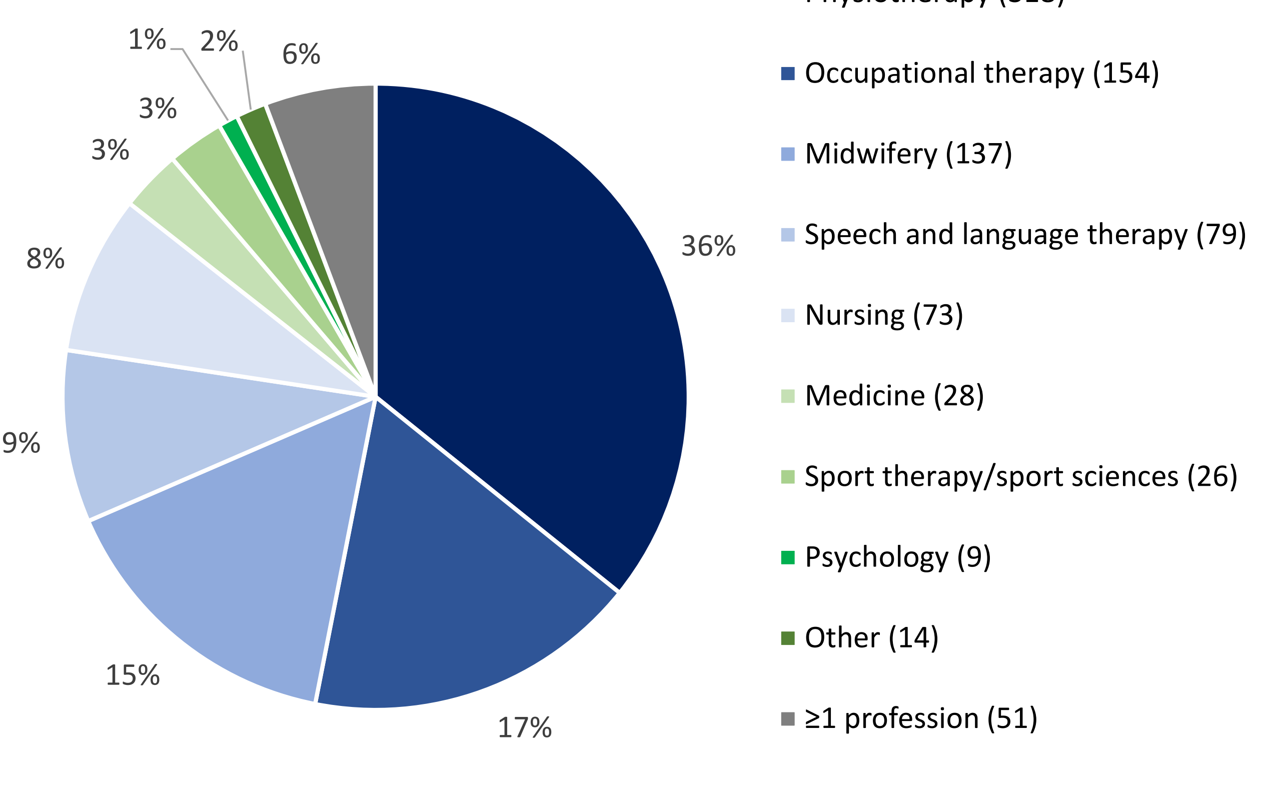

Figure B2 Follow-up sample composition (n = 344)

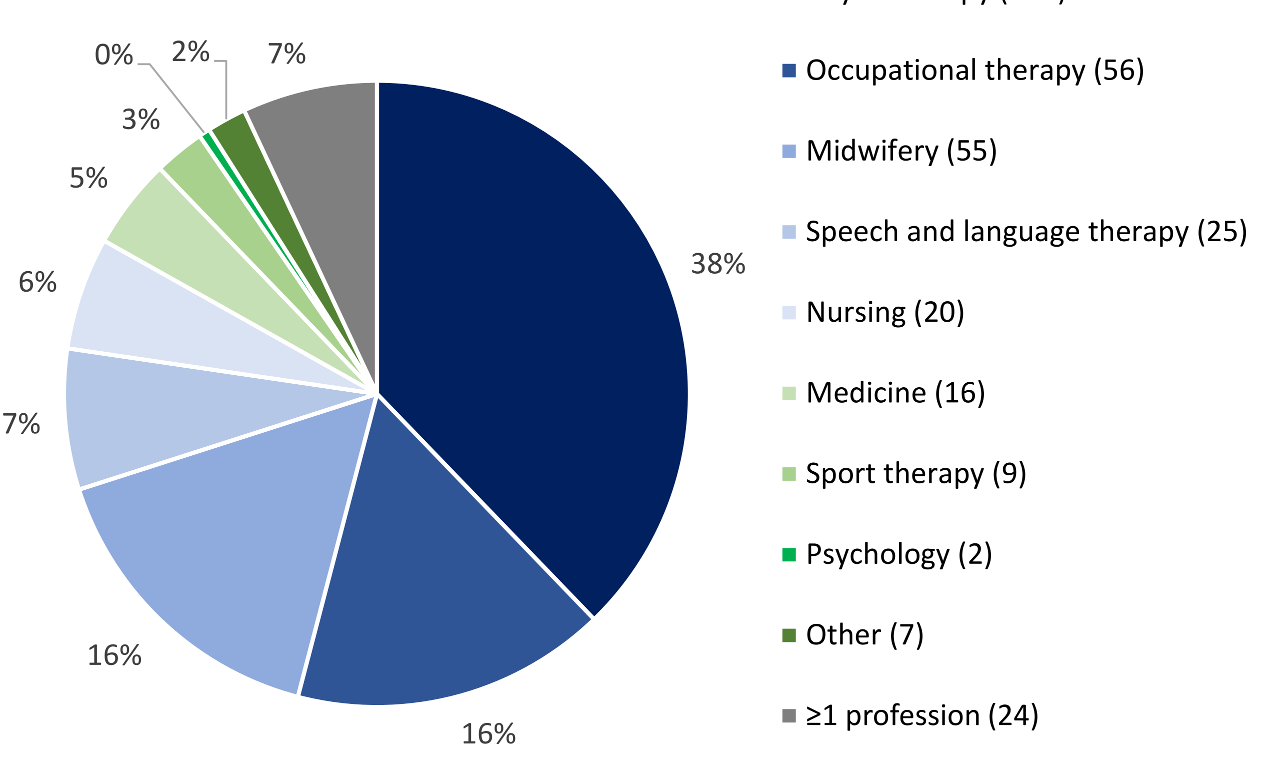

Table A: Socio-demographic characteristics of the follow-up participants according to sub-samples by healthcare profession

Characteristic	Physiotherapy		Occupational therapy		Midwifery	
	Baseline (n = 318)	Follow-up (n = 130)	Baseline (n = 154)	Follow-up (n = 56)	Baseline (n = 137)	Follow-up (n = 55)
Age in years, mean	37.0 ± 11.7 (20 – 69)	37.5 ± 11.7 (22 – 69)	34.5 ± 11.3 (20 – 66)	33.5 ± 10.5 (22 – 60)	41.3 ± 11.7 (21 – 64)	41.0 ± 11.2 (24 – 64)
Gender: male/female/other, n (%)	128/188/0 (40.5/59.5) (n = 316)	48/82/0 (36.9/63.1) (n = 130)	12/136/1 (11.0/88.3/0.6) (n = 154)	3/52/1 (5.4/92.9/1.8) (n = 56)	0/136/1 (0/99.3/0.7) (n = 137)	0/54/1 (0/98.2/1.8) (n = 55)
Highest professional degree, n (%)						
Undergraduate	25 (7.9)	8 (6.2)	13 (8.4)	4 (7.1)	22 (16.1)	7 (12.7)
Diploma (vocational school)	142 (44.7)	53 (40.8)	75 (48.7)	26 (46.4)	57 (41.6)	22 (40.0)
Bachelor/diploma (university)	90 (28.3)	35 (26.9)	56 (36.4)	22 (39.3)	46 (33.6)	20 (36.4)
Master	50 (15.7)	30 (23.1)	8 (5.2)	4 (7.1)	8 (5.8)	5 (9.1)
Higher academic degree	7 (2.2)	3 (2.3)	0 (0.0)	0 (0.0)	0 (0)	0 (0.0)
Missing	4 (1.3)	1 (0.8)	2 (1.3)	0 (0.0)	4 (2.9)	1 (1.8)
Contact time with patients, hours per week, mean	28.8 ± 11.8 (1 – 60) (n = 310)	28.1 ± 12.3 (1 – 55) (n = 129)	27.1 ± 10.4 (3 – 50) (n = 150)	28.0 ± 9.9 (6 – 50) (n = 56)	26.8 ± 11.2 (1 – 60) (n = 131)	24.1 ± 12.3 (1 – 50) (n = 55)
Primary setting of work, n (%)						
Hospital	32 (10.6)	11 (8.5)	37 (24.0)	17 (30.4)	55 (40.1)	22 (40.0)
University clinic	19 (6.0)	5 (3.8)	3 (1.9)	2 (3.6)	8 (5.8)	2 (3.6)
Rehabilitation clinic	27 (8.5)	12 (9.2)	10 (6.5)	4 (7.1)	0 (0.0)	0 (0.0)
Outpatient clinic/private practice	215 (67.6)	92 (70.8)	86 (55.8)	28 (50.0)	62 (45.3)	28 (50.9)
Other	19 (6.0)	9 (6.9)	16 (10.4)	5 (8.9)	12 (8.8)	3 (5.5)
Missing	6 (1.9)	1 (0.8)	2 (1.3)	0 (0.0)	0 (0)	0 (0.0)
Work experience in years, mean	13.8 ± 11.3 (0 – 42) (n = 314)	14.1 ± 11.6 (0 – 42) (n = 130)	9.6 ± 8.5 (0 – 44) (n = 151)	8.7 ± 7.9 (0 – 33) (n = 55)	17.3 ± 11.5 (0 – 42) (n = 132)	16.0 ± 10.9 (0 – 40) (n = 54)
Employment situation, n (%)						
Employee/worker	222 (69.8)	95 (73.1)	115 (74.7)	46 (82.1)	53 (38.7)	22 (40.0)
Self-employed	64 (20.1)	25 (19.2)	25 (16.2)	5 (8.9)	21 (15.3)	9 (16.4)
Freelancer	9 (2.8)	5 (3.8)	6 (3.9)	2 (3.6)	52 (38.0)	22 (40.0)
Undergraduate/in training/practical year	20 (6.3)	5 (3.8)	8 (5.2)	3 (5.4)	10 (7.3)	2 (3.6)
Missing	3 (0.9)	0 (0.0)	0 (0.0)	0 (0.0)	1 (0.7)	0 (0.0)
Leading/leadership position: yes/no, n (%)	103/209 (33.0/67.0)	42/88 (32.3/67.7)	57/96 (37.0/62.3)	17/39 (30.4/69.6)	24/111 (17.8/82.2)	8 (14.5)
Having inter-professional communication, n (%)	239 (75.6)	95 (73.1)	145 (94.2)	54 (96.4)	120 (87.6)	51 (92.7)
Size of the city/municipality of employment, n (%)						
< 5.000 (rural community)	19 (6.0)	7 (5.4)	13 (8.4)	4 (7.1)	11 (8.0)	3 (5.5)
5.000 – 20.000 (small town)	55 (17.3)	16 (12.3)	37 (24.0)	12 (21.4)	21 (15.3)	12 (21.8)
20.000 – 100.000 (mean sized city)	78 (24.5)	37 (28.5)	43 (27.9)	15 (26.8)	51 (37.2)	18 (32.7)
> 100.000 (large city)	163 (51.3)	70 (53.8)	61 (39.6)	25 (44.6)	53 (38.7)	22 (40.0)
Missing	3 (0.9)	0 (0.0)	0 (0.0)	0 (0.0)	1 (0.7)	0 (0.0)
Available time for scientific literature studies at work within a typical week in minutes per week, mean	42.3 ± 88.9 (0 – 600) (n = 274)	45.1 ± 103.4 (0 – 600) (n = 122)	41.7 ± 54.9 (0 – 300) (n = 127)	37.5 ± 42.8 (0 – 180) (n = 49)	48.3 ± 95.1 (0 – 900) (n = 107)	60.8 ± 139.5 (0 – 900) (n = 43)
Availability of scientific literature at work place, n (%)	193 (61.7)	82 (63.6)	94 (61.4)	36 (64.3)	102 (79.1)	42 (82.4)
Drafting of or involvement in ≥1 scientific publication, n (%)	112 (35.3)	52 (40.0)	34 (22.1)	11 (19.6)	28 (20.4)	16 (29.1)
Hosting of lectures or workshops on evidence-based practice, n (%)	55 (17.3)	33 (25.4)	9 (5.9)	3 (5.4)	9 (6.6)	6 (10.9)

**Table B** Test-retest reliability of the German language version of the Evidence-based Practice Inventory (EBPI) for the sub-sample of physiotherapists (n = 130)					
Dimension	Dimension 1	Dimension 2	Dimension 3	Dimension 4	Dimension 5
Description of dimension	Attitude	Subjective norm	Perceived behavioural control	Decision making	Intention and behaviour
Items included in dimension	Item 1-8	Item 9-13	Item 14-19	Item 20-22	Item 23-26
Scale range	8-48	5-30	6-36	3-18	4-24
Number completed dimension	130	126	130	130	130
Mean ± SD score 1st measure	39.2 ± 7.7	17.4 ± 6.1	28.8 ± 5.1	13.0 ± 2.9	15.9 ± 4.7
Mean ± SD score 2nd measure	39.5 ± 6.8	18.2 ± 5.9	29.0 ± 5.3	13.1 ± 2.8	16.0 ± 4.4
Mean difference absolute (95% CI)	0.3 (-0.3 to 0.9)	0.5 (0.0 to 1.0)	0.2 (-0.3 to 0.7)	0.2 (-0.2 to 0.5)	0.1 (-0.2 to 0.5)
Mean difference relative to score of 1st measure	0.8%	2.9%	0.7%	1.5%	0.6%
P value for mean difference	0.33	0.05	0.36	0.31	0.50
σ^2_p	46.1	32.2	23.1	6.4	18.0
σ^2_o	0.0	0.1	0.0	0.0	0.0
$\sigma^2_{residual}$	6.3	3.9	3.8	1.6	2.4
$ICC_{AGREEMENT}$	0.88	0.89	0.86	0.80	0.88
95% CI for ICC	0.83 to 0.91	0.85 to 0.92	0.81 to 0.90	0.73 to 0.85	0.84 to 0.92
P value for ICC	<0.01	<0.01	<0.01	<0.01	<0.01
$SEM_{AGREEMENT}$ (absolute value)	2.5	2.0	2.0	1.3	1.6
$SEM_{AGREEMENT}$ (relative to scale range)	6.3%	8.0%	6.7%	8.7%	8.0%
τ -correlation [a]	-0.33	-0.11	-0.24	-0.06	-0.15
Normal distribution of differences [b]	p < 0.01	p < 0.01	p < 0.01	p < 0.01	p < 0.01
95% LoA (log as function of X)	-0.22X + 0.3 to 0.22X + 0.3	-0.35X + 0.5 to 0.35X + 0.5	-0.21X + 0.2 to 0.21X + 0.2	-0.29X + 0.2 to 0.29X + 0.2	-0.33X + 0.1 to 0.33X + 0.5
MDC_{90}	5.8	4.6	4.5	3.0	3.6
MDC_{95}	7.0	5.5	5.4	3.5	4.3

SD = standard deviation; CI = confidence interval; σ^2_p = variance between participants; σ^2_o = variance due to systematic differences between questionnaire administrations; $\sigma^2_{residual}$ = residual variance; ICC = intraclass correlation coefficient ; SEM = standard error of measurement; LoA = absolute limites of agreement with 95% confidence; X = test score; MDC_{90} = minimal detectable change with 90% confidence; MDC_{95} = minimal detectable change with 95% confidence

[a] Kendall's Tau correlation between absolute difference and mean scores of two measures; [b] Shapirow Wilk test of Normality

**Table C** Test-retest reliability of the German language version of the Evidence-based Practice Inventory (EBPI) for the sub-sample of occupational therapists (n = 56)					
Dimension	Dimension 1	Dimension 2	Dimension 3	Dimension 4	Dimension 5
Description of dimension	Attitude	Subjective norm	Perceived behavioural control	Decision making	Intention and behaviour
Items included in dimension	Item 1-8	Item 9-13	Item 14-19	Item 20-22	Item 23-26
Scale range	8-48	5-30	6-36	3-18	4-24
Number completed dimension	56	56	56	55	56
Mean ± SD score 1st measure	36.9 ± 5.0	16.5 ± 4.9	25.3 ± 6.0	11.8 ± 2.2	13.6 ± 2.8
Mean ± SD score 2nd measure	37.4 ± 6.1	17.2 ± 5.3	25.8 ± 6.4	12.4 ± 2.3	14.0 ± 3.0
Mean difference absolute (95% CI)	0.5 (-0.6 to 1.6)	0.7 (-0.1 to 1.6)	0.5 (-1.0 to 2.0)	0.6 (0.1 to 1.0)	0.4 (-0.2 to 0.9)
Mean difference relative to score of 1st measure	1.4%	4.2%	2.0%	5.1%	2.9%
P value for mean difference	0.34	0.10	0.49	0.01	0.21
σ^2_p	22.7	20.9	22.9	3.8	6.2
σ^2_o	0.0	0.2	0.0	0.1	0.0
$\sigma^2_{residual}$	7.9	5.1	15.2	1.4	2.2
$ICC_{AGREEMENT}$	0.74	0.80	0.60	0.71	0.73
95% CI for ICC	0.60 to 0.84	0.68 to 0.88	0.41 to 0.75	0.54 to 0.83	0.58 to 0.83
P value for ICC	<0.01	<0.01	<0.01	<0.01	<0.01
$SEM_{AGREEMENT}$ (absolute value)	2.8	2.3	3.9	1.2	1.5
$SEM_{AGREEMENT}$ (relative to scale range)	7.0%	9.2%	13.0%	8.0%	7.5%
τ -correlation [a]	-0.12	0.16	-0.26	-0.16	-0.05
Normal distribution of differences [b]	p < 0.01	P = 0.36	p < 0.01	0.03	0.03
95% LoA (log as function of X)	-0.36X + 0.5 to 0.36X + 0.5	-0.38X + 0.7 to 0.38X + 0.7	-0.62X + 0.5 to 0.62X + 0.5	-0.30X + 0.6 to 0.30X + 0.6	-0.32X + 0.4 to 0.32X + 0.4
MDC_{90}	6.5	5.3	9.0	2.9	3.5
MDC_{95}	7.8	6.4	10.8	3.4	4.2

SD = standard deviation; CI = confidence interval; σ^2_p = variance between participants; σ^2_o = variance due to systematic differences between questionnaire administrations; $\sigma^2_{residual}$ = residual variance; ICC = intraclass correlation coefficient ; SEM = standard error of measurement; LoA = absolute limites of agreement with 95% confidence; X = test score; MDC_{90} = minimal detectable change with 90% confidence; MDC_{95} = minimal detectable change with 95% confidence

[a] Kendall's Tau correlation between absolute difference and mean scores of two measures; [b] Shapirow Wilk test of Normality

**Table D** Test-retest reliability of the German language version of the Evidence-based Practice Inventory (EBPI) for the sub-sample of Midwifes (n = 55)					
Dimension	Dimension 1	Dimension 2	Dimension 3	Dimension 4	Dimension 5
Description of dimension	Attitude	Subjective norm	Perceived behavioural control	Decision making	Intention and behaviour
Items included in dimension	Item 1-8	Item 9-13	Item 14-19	Item 20-22	Item 23-26
Scale range	8-48	5-30	6-36	3-18	4-24
Number completed dimension	55	44	55	54	55
Mean ± SD score 1st measure	39.6 ± 5.1	19.3 ± 5.7	27.9 ± 5.4	13.0 ± 2.0	15.4 ± 3.5
Mean ± SD score 2nd measure	39.7 ± 5.0	20.2 ± 5.3	28.6 ± 5.0	13.2 ± 2.2	16.0 ± 3.5
Mean difference absolute (95% CI)	0.1 (-0.8 to 1.0)	1.0 (0.2 to 1.7)	0.7 (-0.2 to 1.6)	0.3 (-0.2 to 0.8)	0.5 (-0.2 to 1.2)
Mean difference relative to score of 1st measure	0.3%	5.2%	2.5%	2.3%	3.2%
P value for mean difference	0.81	0.02	0.11	0.30	0.13
σ^2_p	20.2	26.8	21.8	2.8	8.9
σ^2_o	0.0	0.4	0.2	0.0	0.1
$\sigma^2_{residual}$	5.6	3.4	5.5	1.6	3.2
$ICC_{AGREEMENT}$	0.79	0.88	0.79	0.63	0.73
95% CI for ICC	0.66 to 0.87	0.77 to 0.93	0.67 to 0.87	0.44 to 0.77	0.58 to 0.84
P value for ICC	<0.01	<0.01	<0.01	<0.01	<0.01
$SEM_{AGREEMENT}$ (absolute value)	2.4	1.9	2.4	1.3	1.8
$SEM_{AGREEMENT}$ (relative to scale range)	6.0%	7.6%	8.0%	8.7%	9.0%
τ -correlation [a]	-0.08	-0.14	-0.22	-0.18	-0.09
Normal distribution of differences [b]	p = 0.02	P < 0.01	P < 0.01	0.014	0.28
95% LoA (log as function of X)	-0.17X + 0.1 to 0.17X + 0.1	-0.33X + 1.0 to 0.33X + 1.0	-0.27X + 0.7 to 0.27X + 0.7	-0.28X + 0.3 to 0.28X + 0.3	-4.5 to 5.5
MDC_{90}	5.5	4.5	5.5	3.0	4.2
MDC_{95}	6.5	5.4	6.6	3.5	5.0

SD = standard deviation; CI = confidence interval; σ^2_p = variance between participants; σ^2_o = variance due to systematic differences between questionnaire administrations; $\sigma^2_{residual}$ = residual variance; ICC = intraclass correlation coefficient ; SEM = standard error of measurement; LoA = absolute limites of agreement with 95% confidence; X = test score; MDC_{90} = minimal detectable change with 90% confidence; MDC_{95} = minimal detectable change with 95% confidence

[a] Kendall's Tau correlation between absolute difference and mean scores of two measures; [b] Shapirow Wilk test of Normality

Figure C1: Bland & Altman plot for dimension 1 of the Evidence-based Practice Inventory (EBPI) for the sample of physiotherapists

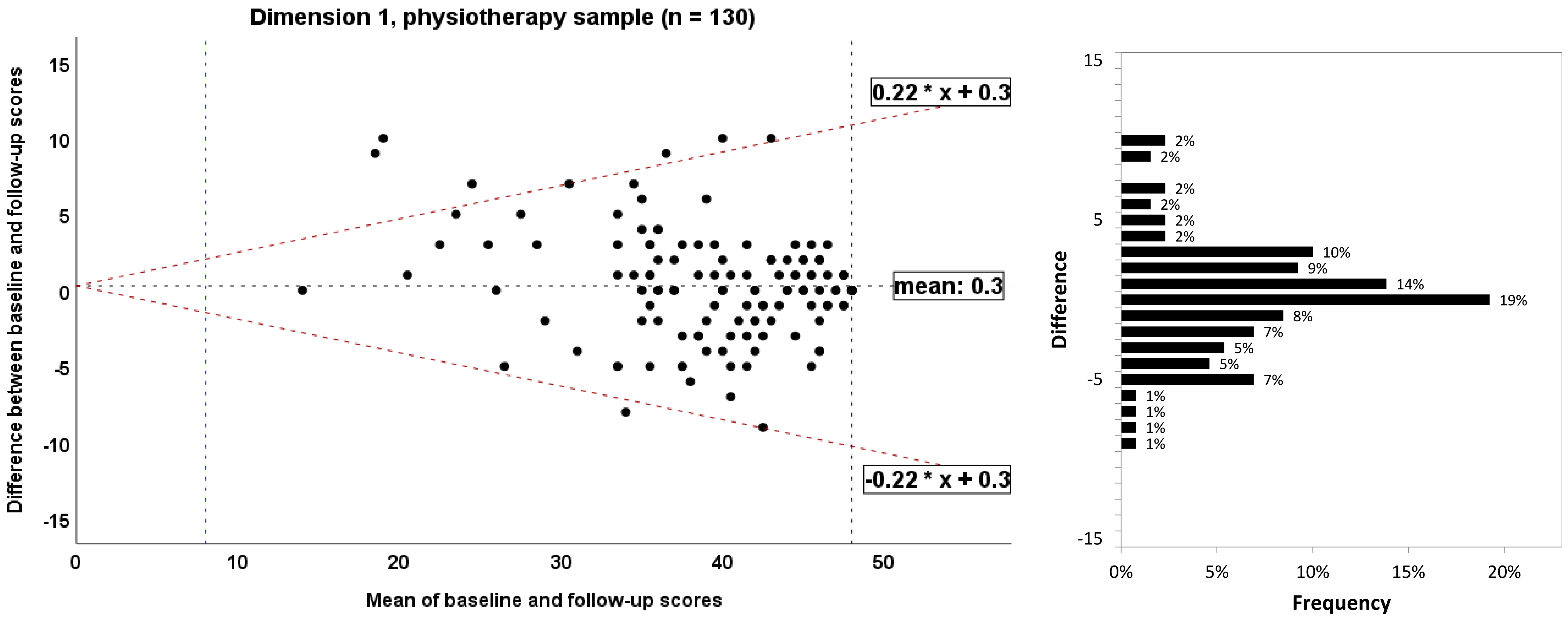

Figure C2: Bland & Altman plot for dimension 2 of the Evidence-based Practice Inventory (EBPI) for the sample of physiotherapists

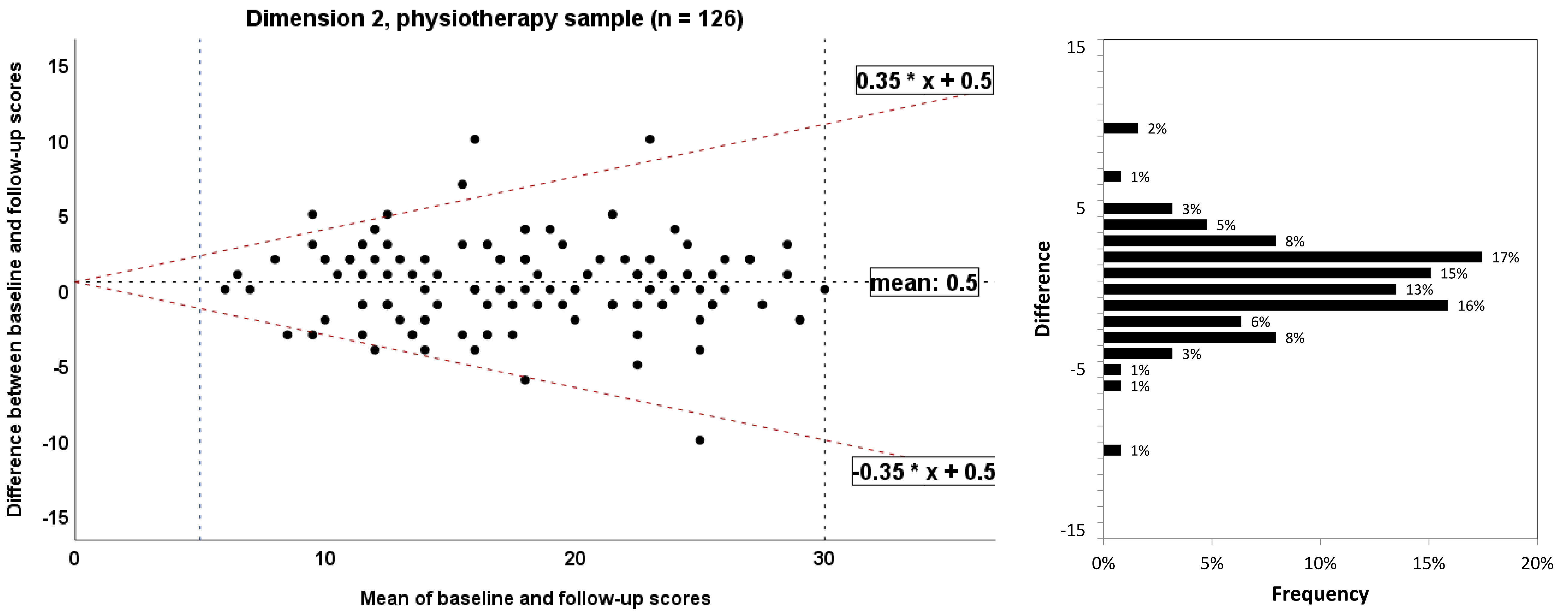

Figure C3: Bland & Altman plot for dimension 3 of the Evidence-based Practice Inventory (EBPI) for the sample of physiotherapists

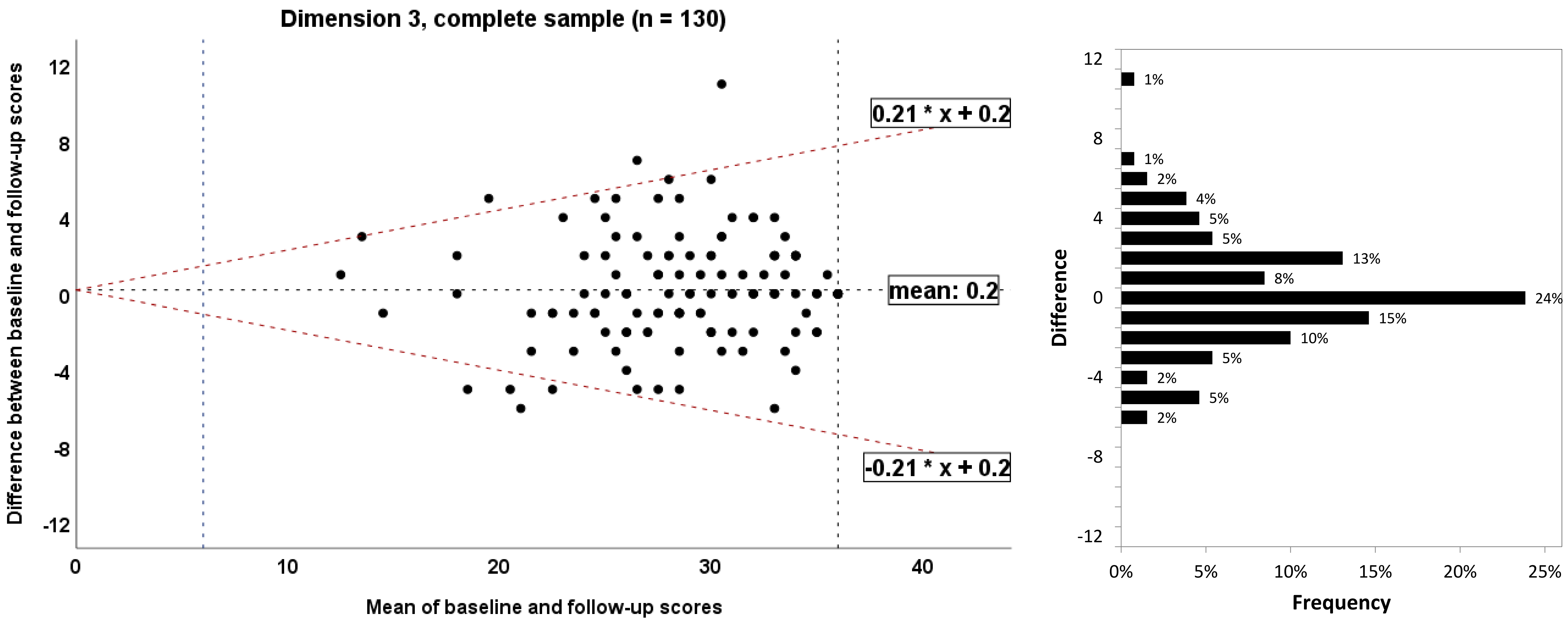

Figure C4: Bland & Altman plot for dimension 4 of the Evidence-based Practice Inventory (EBPI) for the sample of physiotherapists

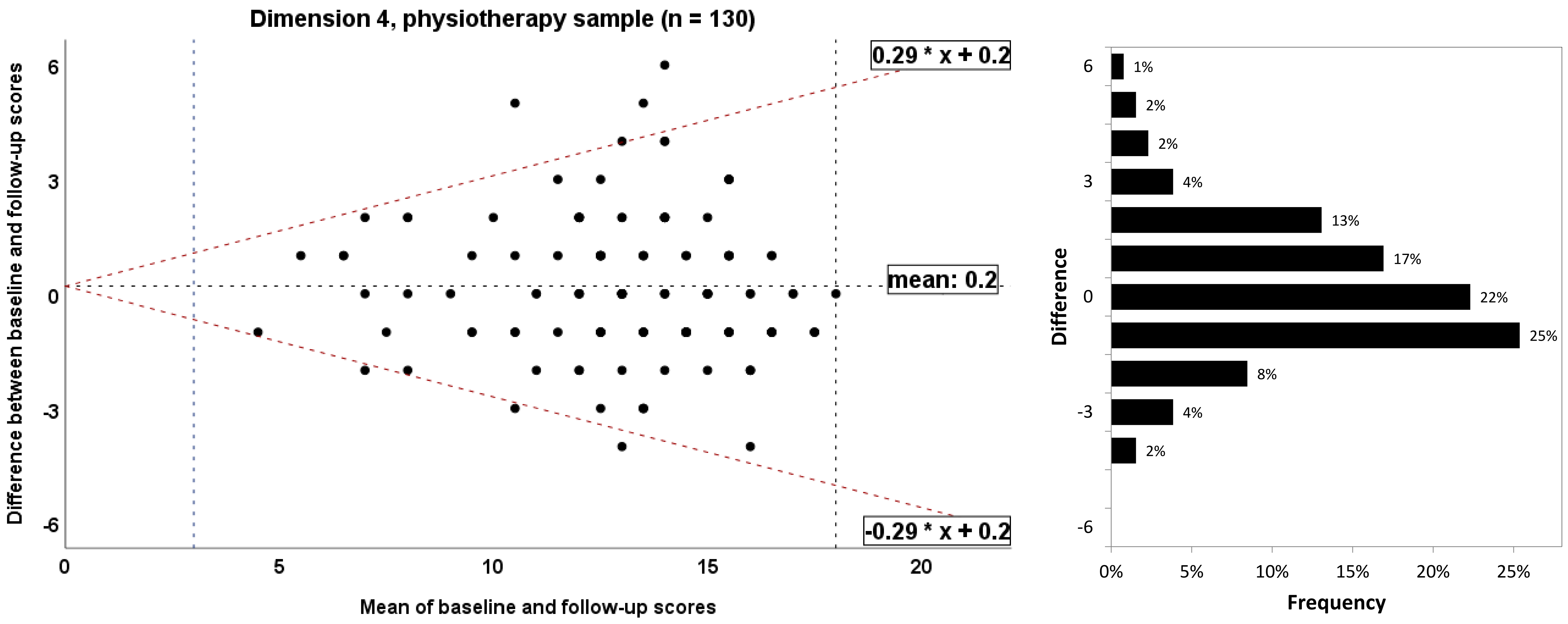

Figure C5: Bland & Altman plot for dimension 5 of the Evidence-based Practice Inventory (EBPI) for the sample of physiotherapists

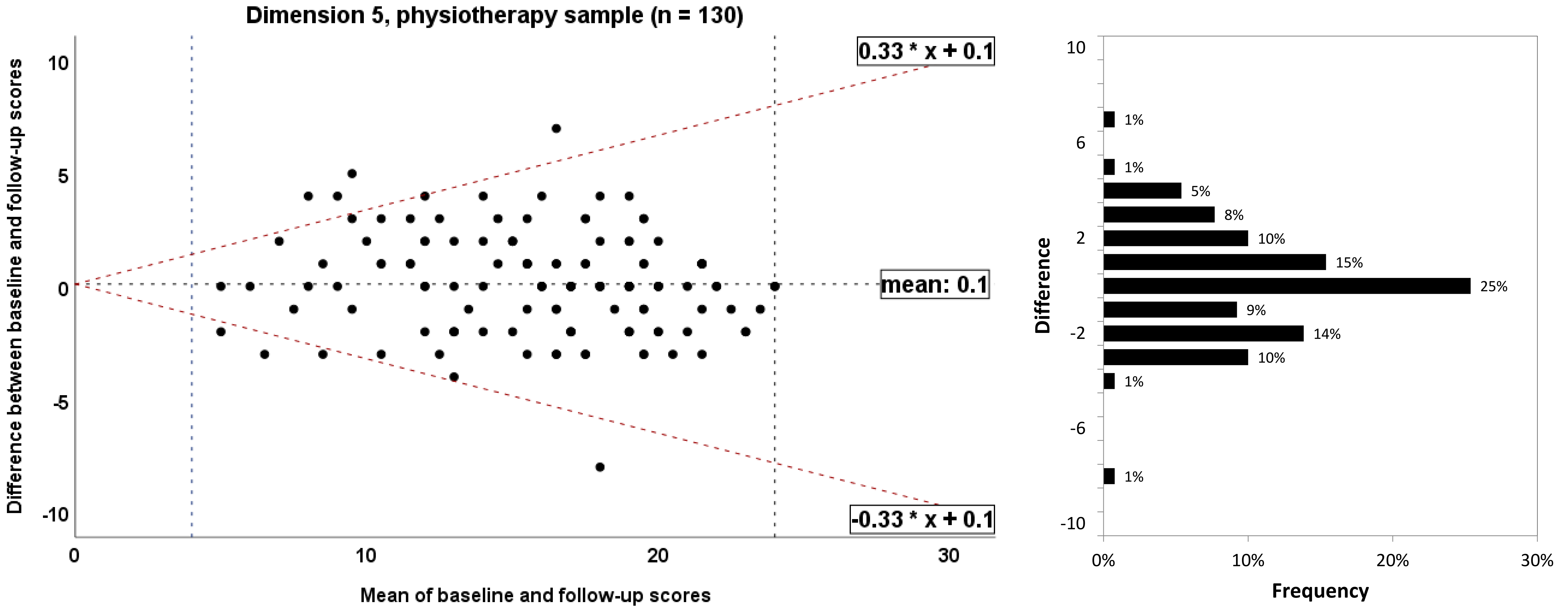

Figure D1: Bland & Altman plot for dimension 1 of the Evidence-based Practice Inventory (EBPI) for the sample of occupational therapists

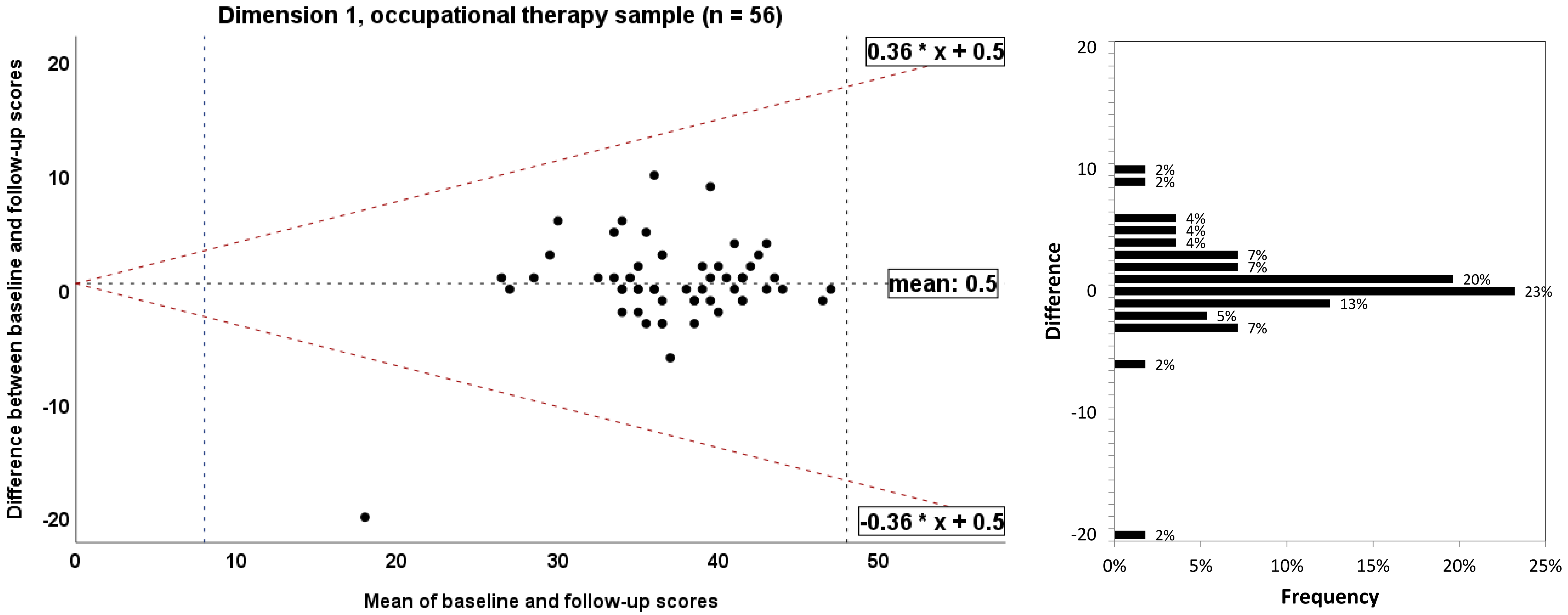

Figure D2: Bland & Altman plot for dimension 2 of the Evidence-based Practice Inventory (EBPI) for the sample of occupational therapists

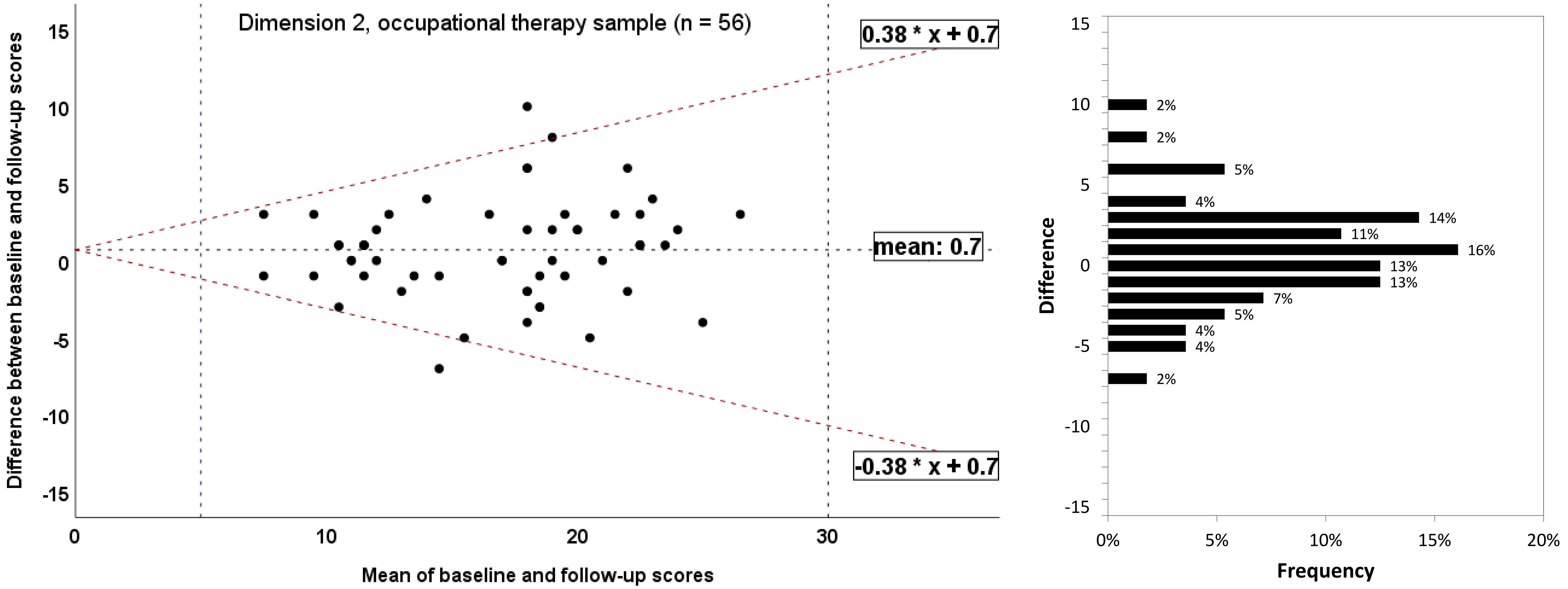

Figure D3: Bland & Altman plot for dimension 3 of the Evidence-based Practice Inventory (EBPI) for the sample of occupational therapists

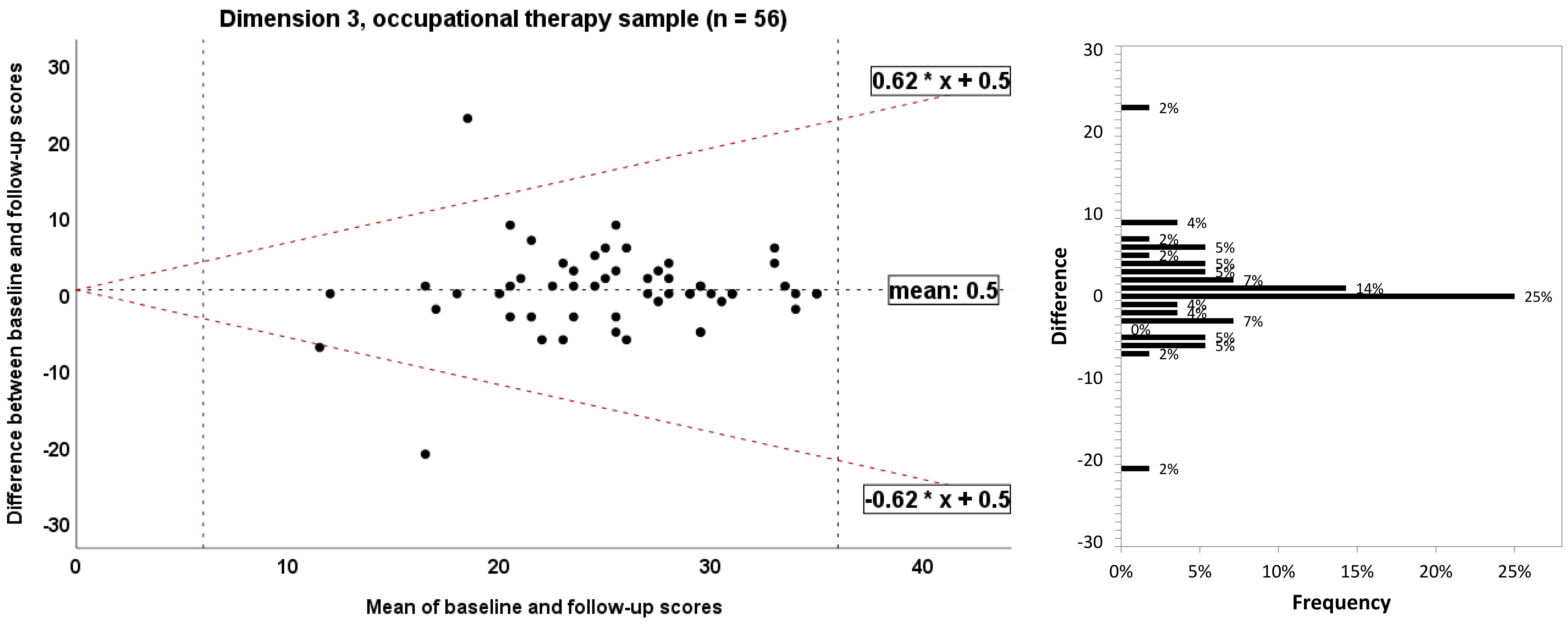

Figure D4: Bland & Altman plot for dimension 4 of the Evidence-based Practice Inventory (EBPI) for the sample of occupational therapists

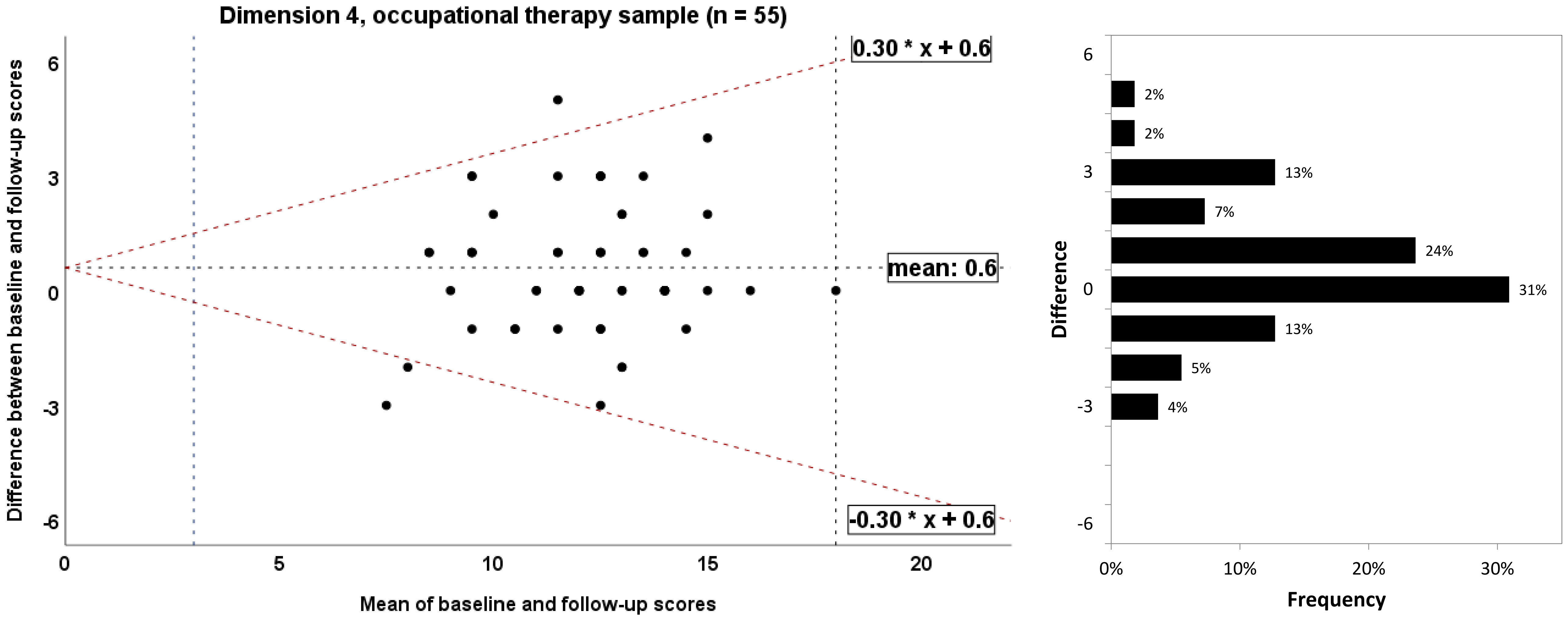

Figure D5: Bland & Altman plot for dimension 5 of the Evidence-based Practice Inventory (EBPI) for the sample of occupational therapists

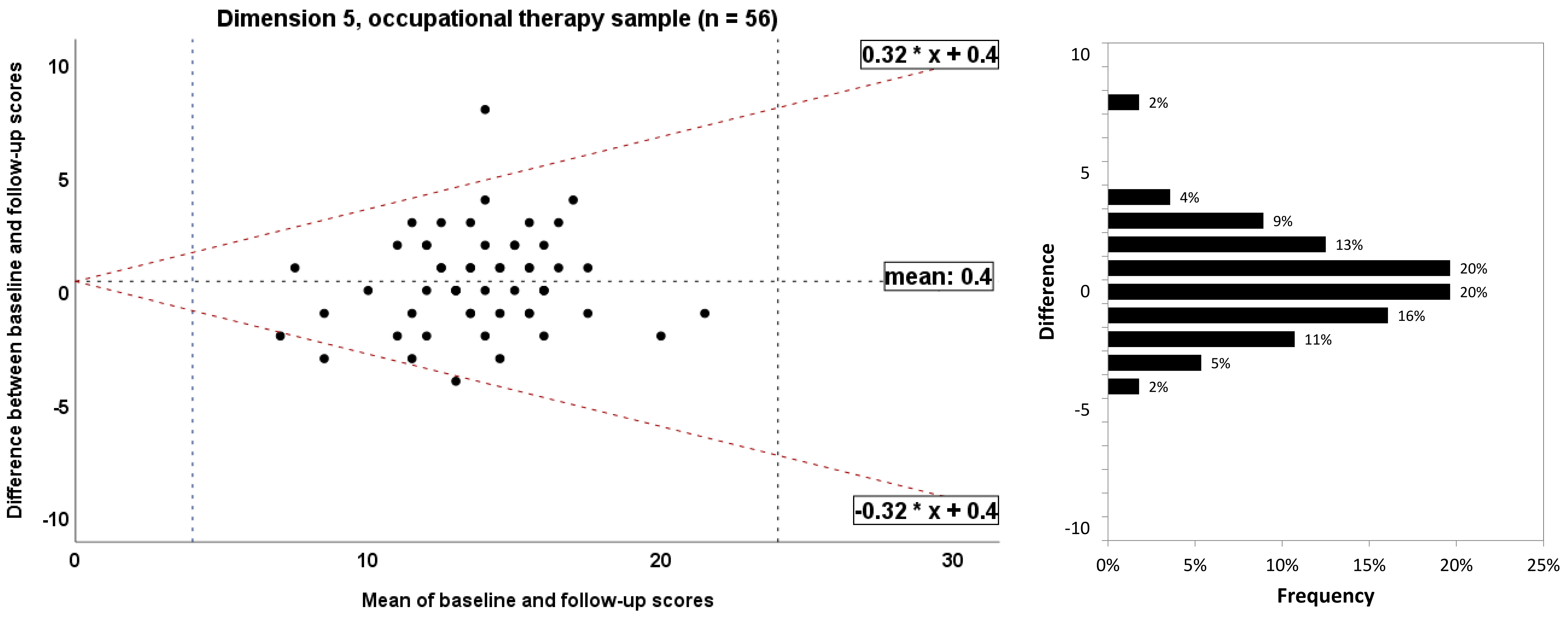

Figure E1: Bland & Altman plot for dimension 1 of the Evidence-based Practice Inventory (EBPI) for the sample of midwifes

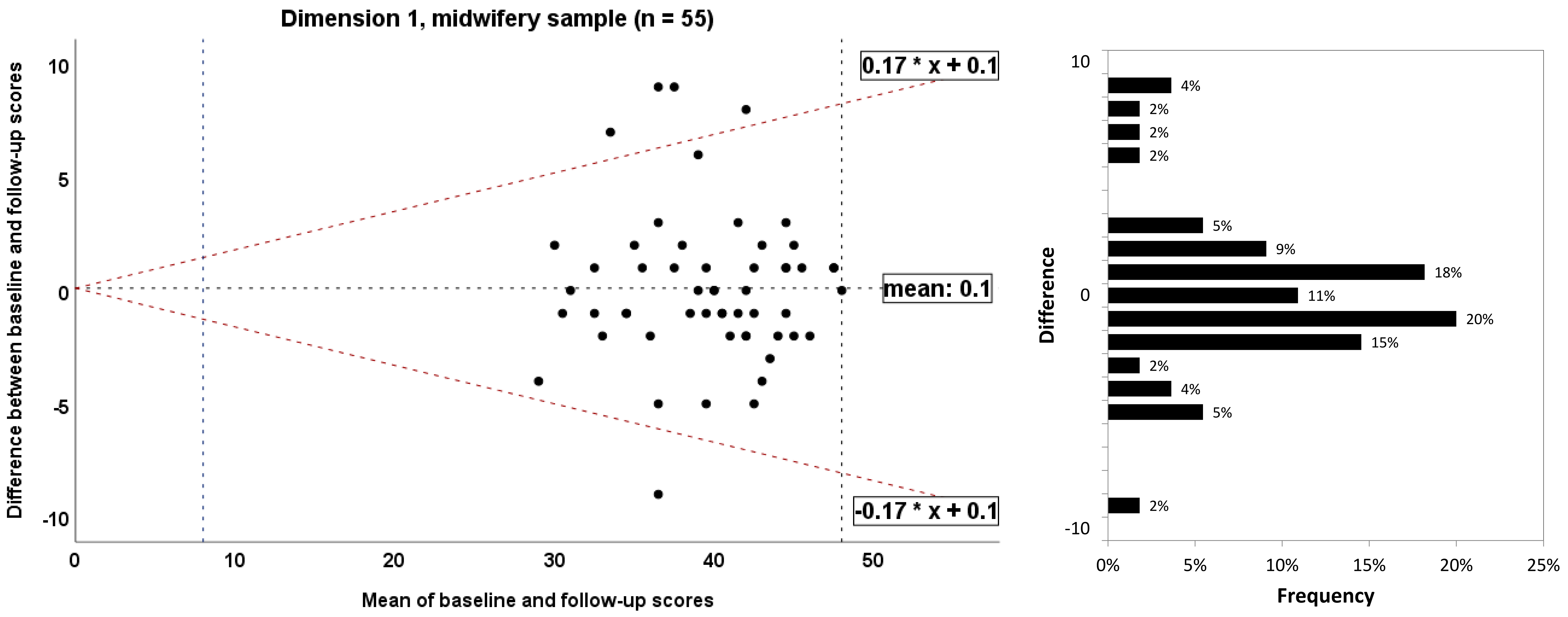

Figure E2: Bland & Altman plot for dimension 2 of the Evidence-based Practice Inventory (EBPI) for the sample of midwifes

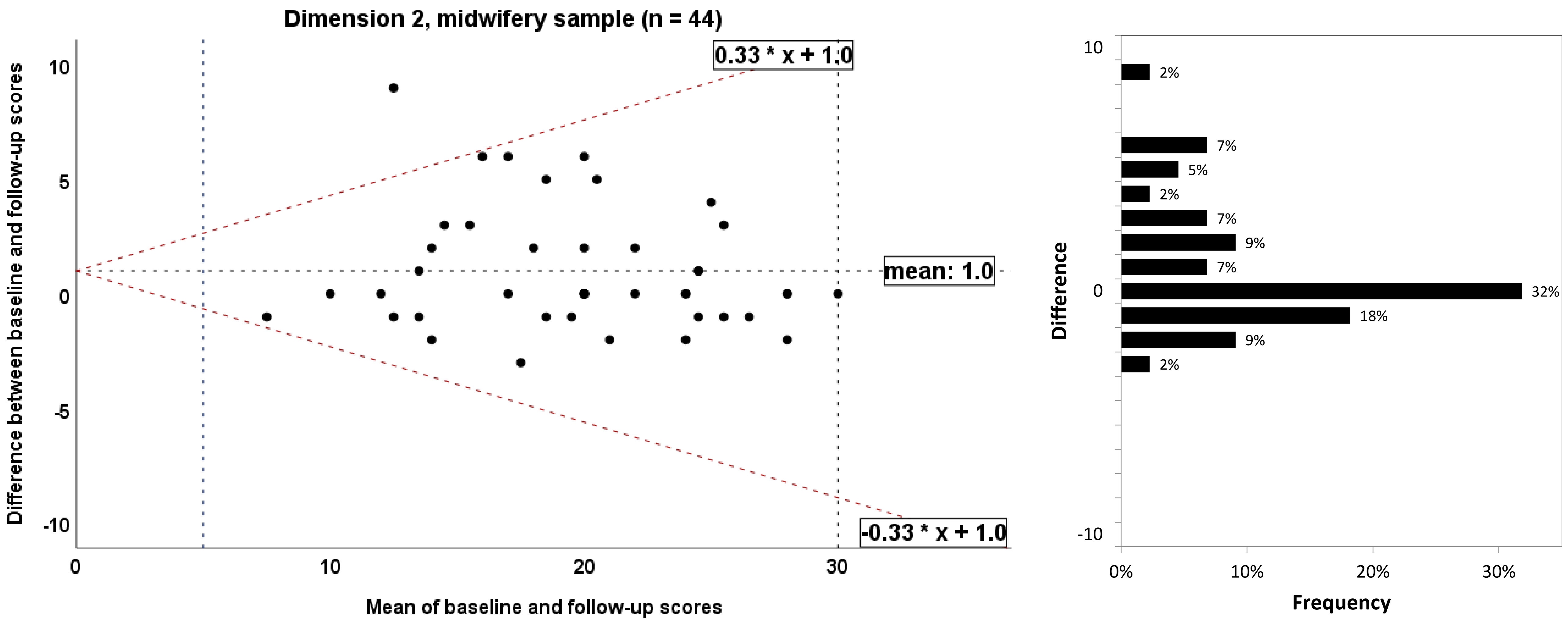

Figure E3: Bland & Altman plot for dimension 3 of the Evidence-based Practice Inventory (EBPI) for the sample of midwifes

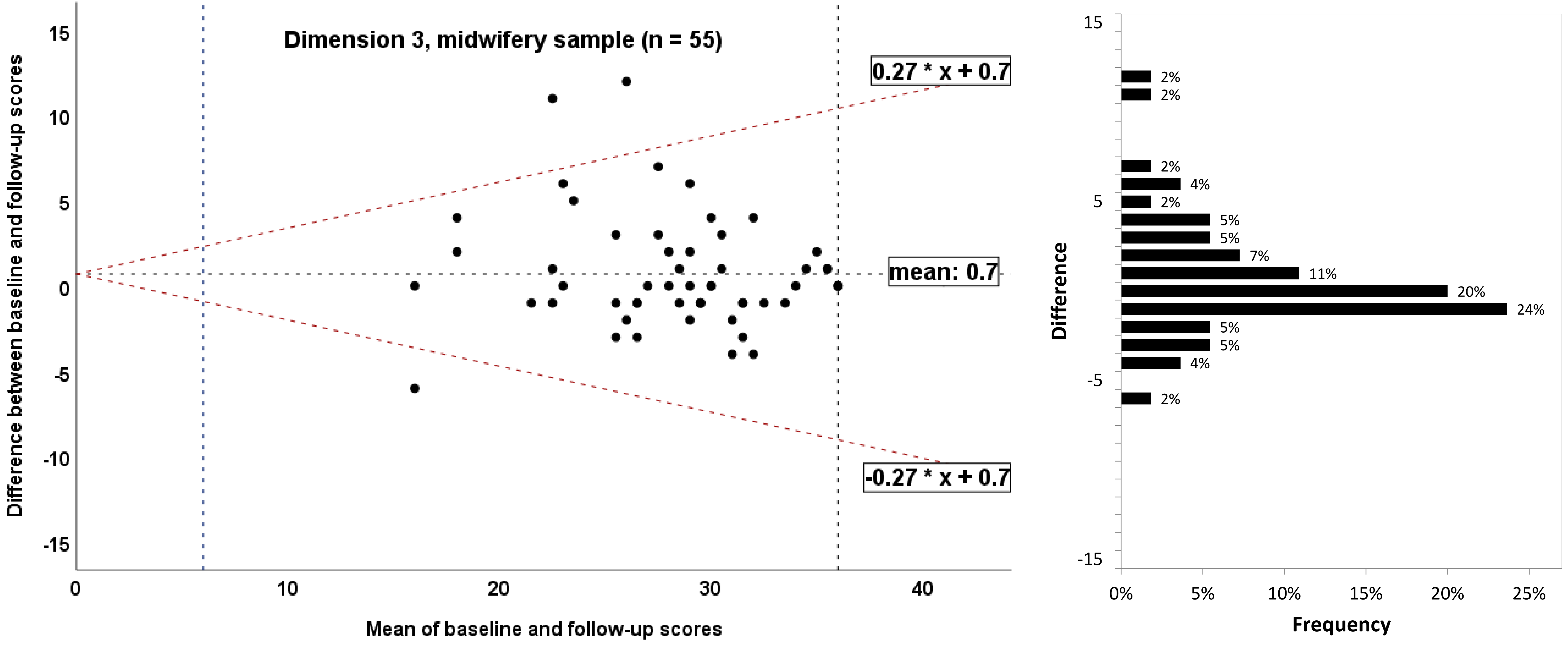

Figure E4: Bland & Altman plot for dimension 4 of the Evidence-based Practice Inventory (EBPI) for the sample of midwifes

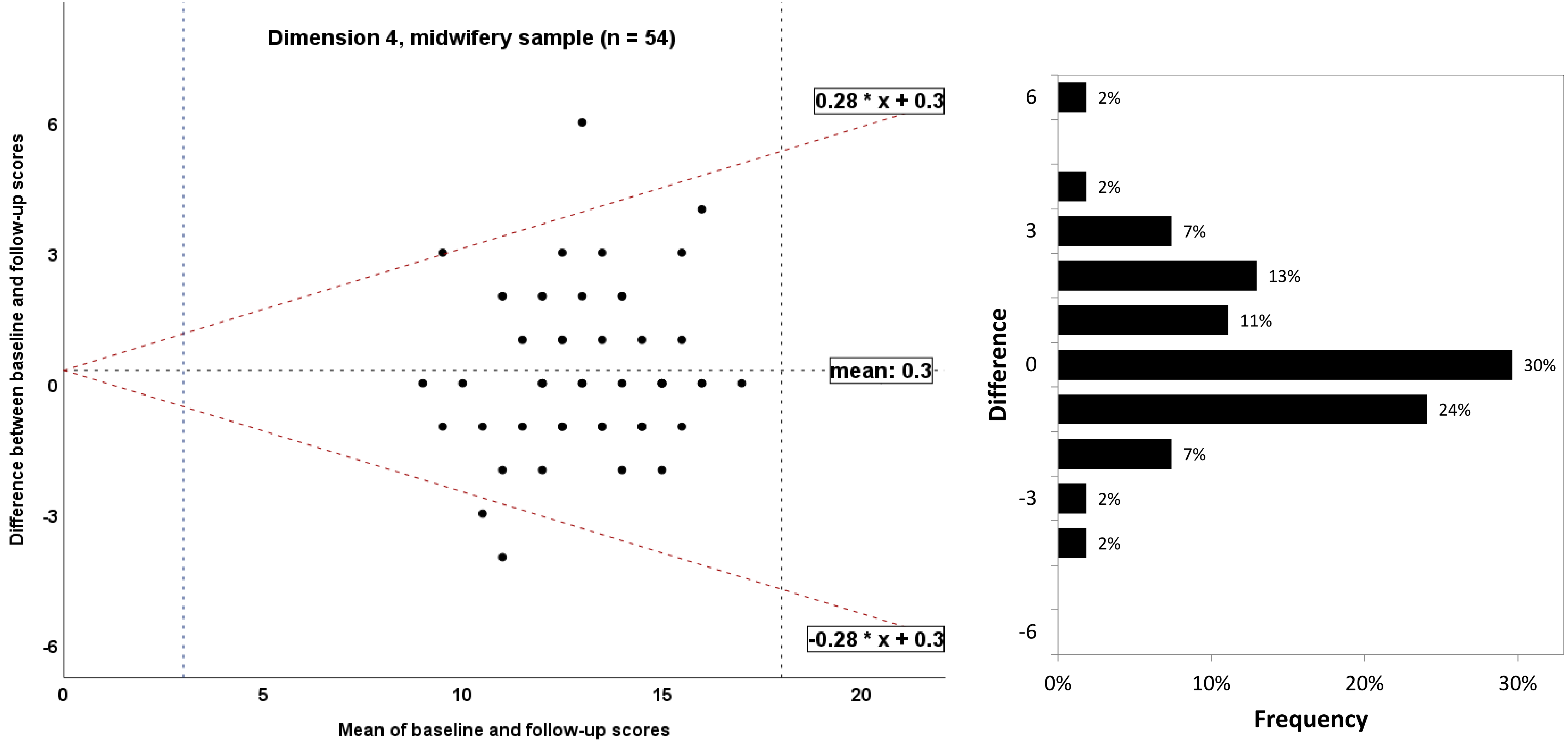

Figure E5: Bland & Altman plot for dimension 5 of the Evidence-based Practice Inventory (EBPI) for the sample of midwifes

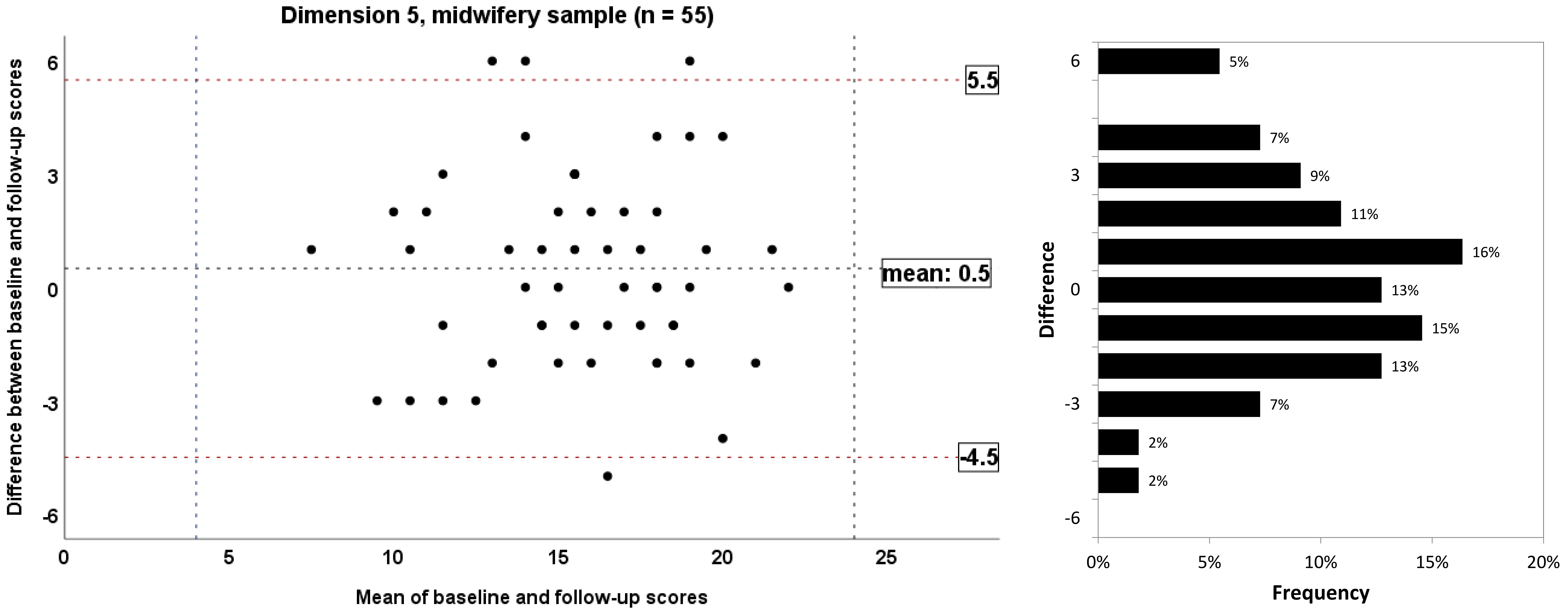

Figure F1: Response pattern per item for the complete sample

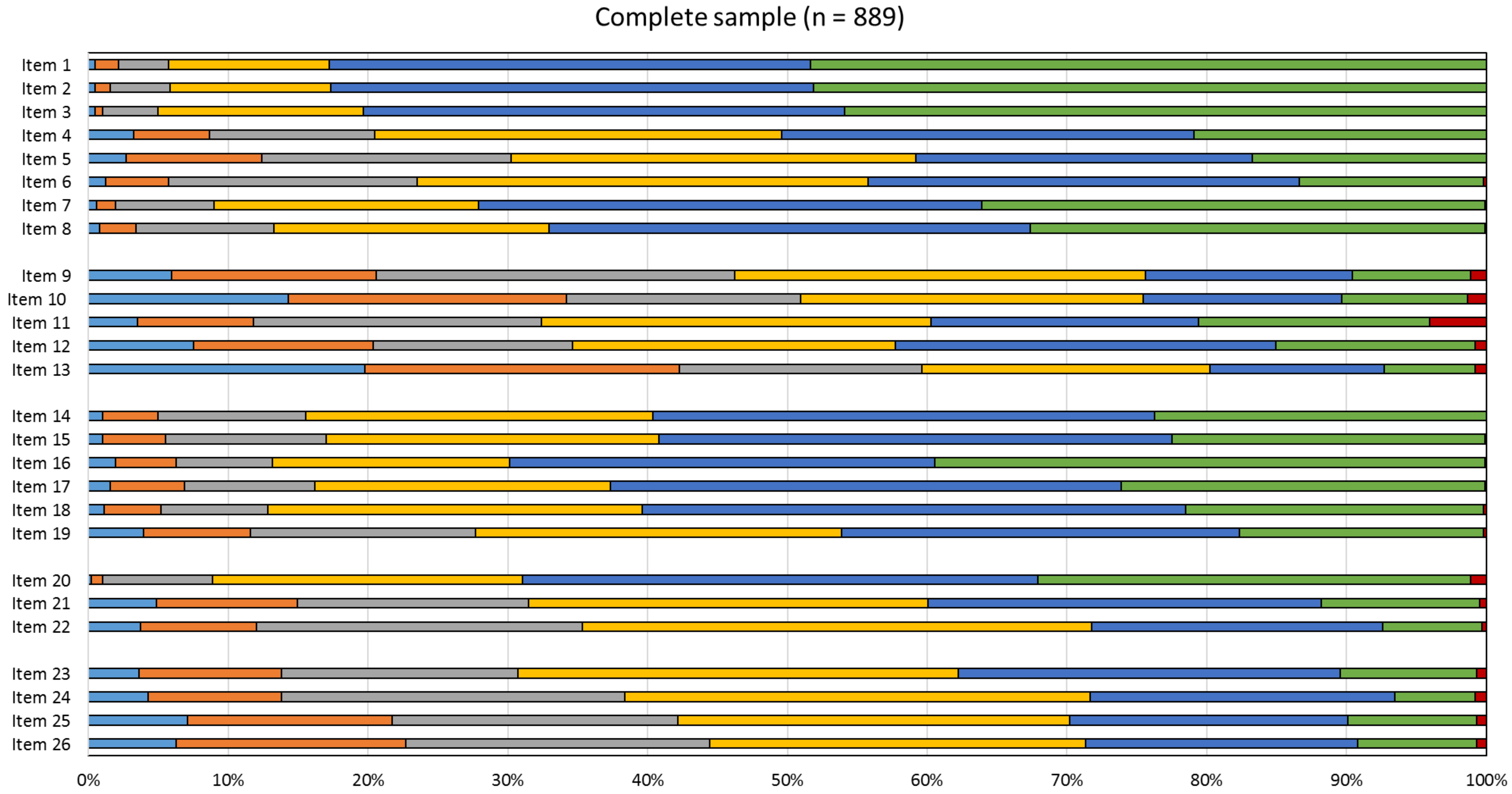

	Item 26	Item 25	Item 24	Item 23	Item 22	Item 21	Item 20	Item 19	Item 18	Item 17	Item 16	Item 15	Item 14	Item 13	Item 12	Item 11	Item 10	Item 9	Item 8	Item 7	Item 6	Item 5	Item 4	Item 3	Item 2	Item 1
Score 1	56	63	38	32	33	43	2	35	10	14	17	9	9	176	67	31	127	53	7	5	11	24	29	4	4	4
Score 2	146	130	85	91	74	90	7	68	36	47	39	40	35	200	114	74	177	130	23	12	40	86	48	5	10	15
Score 3	193	182	218	150	207	147	70	143	68	83	61	102	94	154	127	183	149	228	88	63	158	159	105	35	38	32
Score 4	239	249	296	280	324	254	197	233	238	188	151	212	221	183	205	248	218	261	175	168	287	257	259	131	102	102
Score 5	173	177	194	243	185	250	328	253	346	325	270	326	319	111	242	170	126	132	306	320	274	214	262	306	307	306
Score 6	76	82	51	87	63	101	275	155	189	231	350	199	211	58	127	147	80	75	289	320	117	149	186	408	428	430
missing	6	6	7	6	3	4	10	2	2	1	1	1	0	7	7	36	12	10	1	1	2	0	0	0	0	0

Score 1 Score 2 Score 3 Score 4 Score 5 Score 6 missing

Figure F2: Response pattern per item for the physiotherapy sample

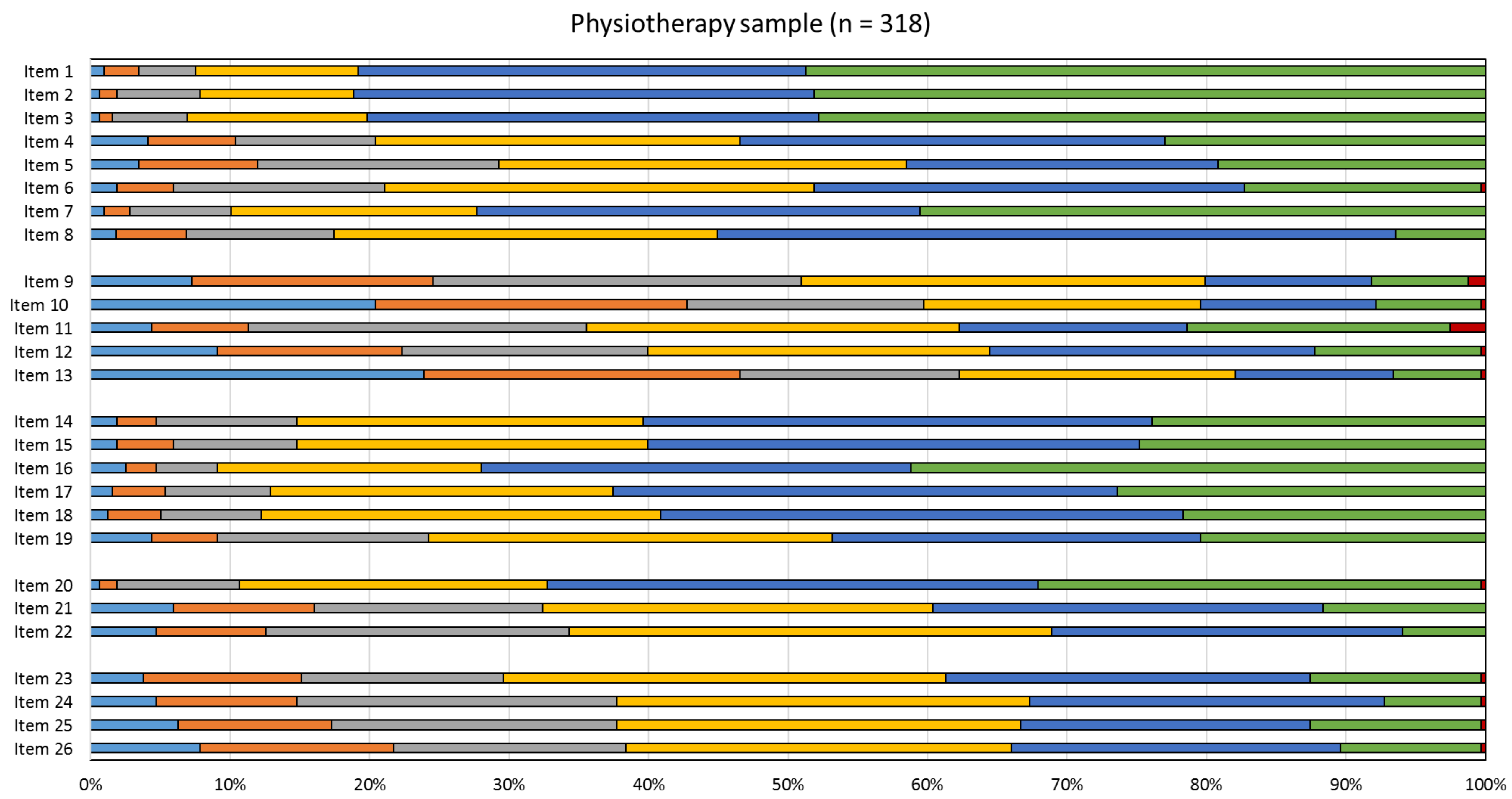

	Item 26	Item 25	Item 24	Item 23	Item 22	Item 21	Item 20	Item 19	Item 18	Item 17	Item 16	Item 15	Item 14	Item 13	Item 12	Item 11	Item 10	Item 9	Item 8	Item 7	Item 6	Item 5	Item 4	Item 3	Item 2	Item 1
Score 1	25	20	15	12	15	19	2	14	4	5	8	6	6	76	29	14	65	23	4	3	6	11	13	2	2	3
Score 2	44	35	32	36	25	32	4	15	12	12	7	13	9	72	42	22	71	55	11	6	13	27	20	3	4	8
Score 3	53	65	73	46	69	52	28	48	23	24	14	28	32	50	56	77	54	84	23	23	48	55	32	17	19	13
Score 4	88	92	94	101	110	89	70	92	91	78	60	80	79	63	78	85	63	92	60	56	98	93	83	41	35	37
Score 5	75	66	81	83	80	89	112	84	119	115	98	112	116	36	74	52	40	38	106	101	98	71	97	103	105	102
Score 6	32	39	22	39	19	37	101	65	69	84	131	79	76	20	38	60	24	22	14	129	54	61	73	152	153	155
missing	1	1	1	1	0	0	1	0	0	0	0	0	0	1	1	8	1	4	0	0	1	0	0	0	0	0

Score 1 Score 2 Score 3 Score 4 Score 5 Score 6 missing

Figure F3: Response pattern per item for the occupational therapy sample

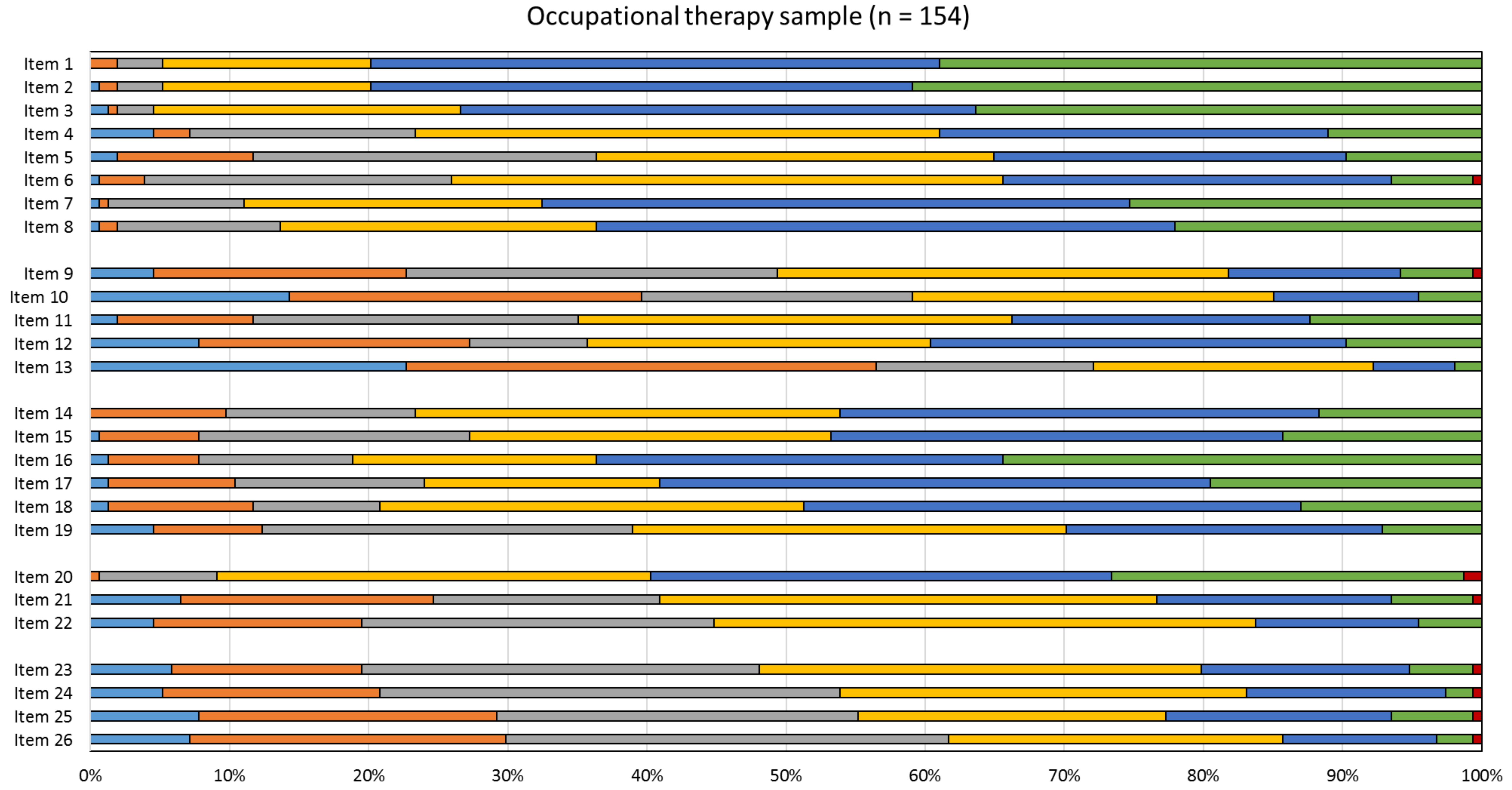

	Item 26	Item 25	Item 24	Item 23	Item 22	Item 21	Item 20	Item 19	Item 18	Item 17	Item 16	Item 15	Item 14	Item 13	Item 12	Item 11	Item 10	Item 9	Item 8	Item 7	Item 6	Item 5	Item 4	Item 3	Item 2	Item 1
Score 1	11	12	8	9	7	10	0	7	2	2	2	1	0	35	12	3	22	7	1	1	1	3	7	2	1	0
Score 2	35	33	24	21	23	28	1	12	16	14	10	11	15	52	30	15	39	28	2	1	5	15	4	1	2	3
Score 3	49	40	51	44	39	25	13	41	14	21	17	30	21	24	13	36	30	41	18	15	34	38	25	4	5	5
Score 4	37	34	45	49	60	55	48	48	47	26	27	40	47	31	38	48	40	50	35	33	61	44	58	34	23	23
Score 5	17	25	22	23	18	26	51	35	55	61	45	50	53	9	46	33	16	19	64	65	43	39	43	57	60	63
Score 6	4	9	3	7	7	9	39	11	20	30	53	22	18	3	15	19	7	8	34	39	9	15	17	56	63	60
missing	1	1	1	1	0	1	2	0	0	0	0	0	0	0	0	0	0	1	0	0	1	0	0	0	0	0

Score 1 Score 2 Score 3 Score 4 Score 5 Score 6 missing

Figure F4: Response pattern per item for the midwifery sample

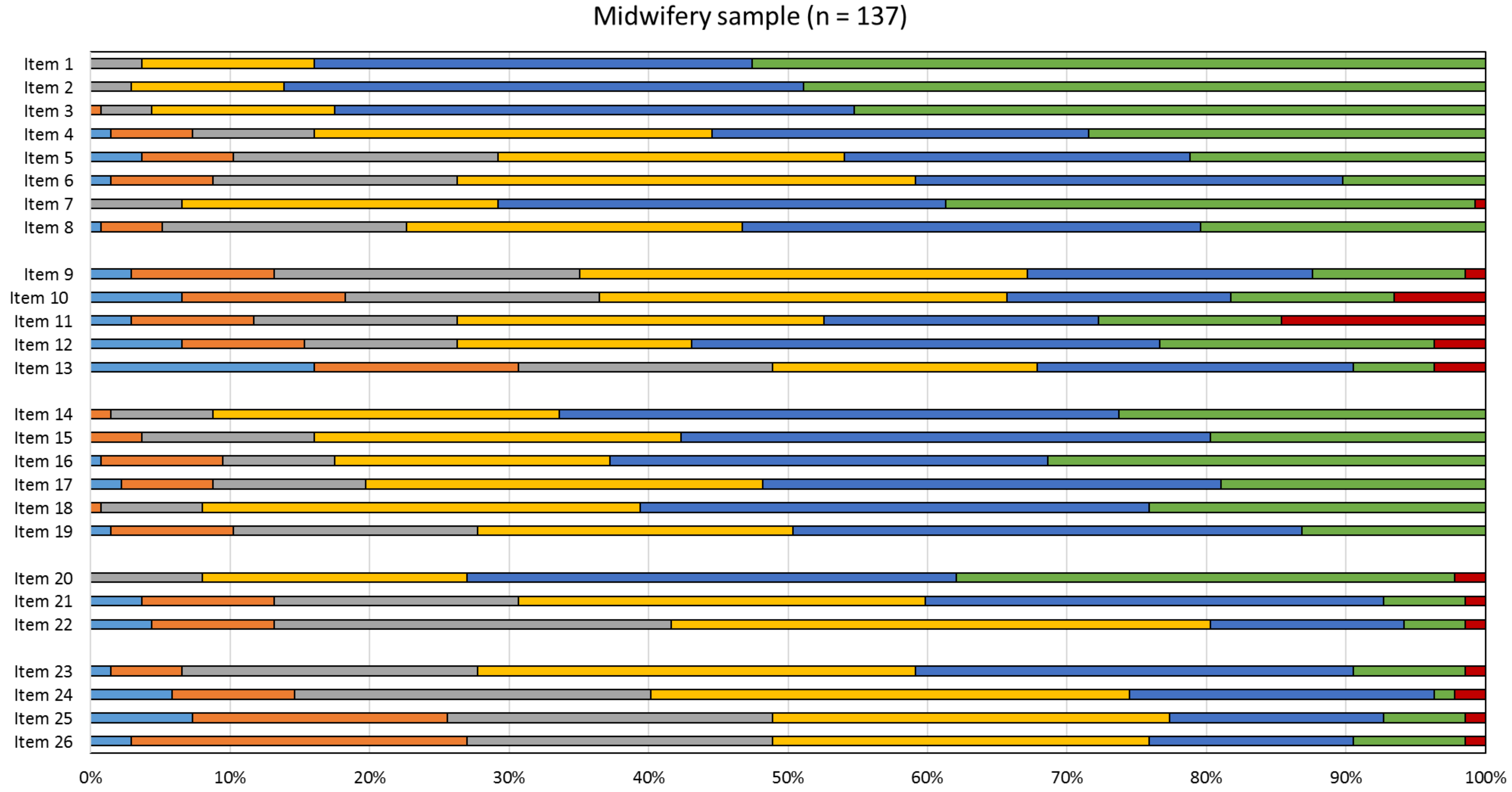

	Item 26	Item 25	Item 24	Item 23	Item 22	Item 21	Item 20	Item 19	Item 18	Item 17	Item 16	Item 15	Item 14	Item 13	Item 12	Item 11	Item 10	Item 9	Item 8	Item 7	Item 6	Item 5	Item 4	Item 3	Item 2	Item 1
Score 1	4	10	8	2	6	5	0	2	0	3	1	0	0	22	9	4	9	4	1	0	2	5	2	0	0	0
Score 2	33	25	12	7	12	13	0	12	1	9	12	5	2	20	12	12	16	14	6	0	10	9	8	1	0	0
Score 3	30	32	35	29	39	24	11	24	10	15	11	17	10	25	15	20	25	30	24	9	24	26	12	5	4	5
Score 4	37	39	47	43	53	40	26	31	43	39	27	36	34	26	23	36	40	44	33	31	45	34	39	18	15	17
Score 5	20	21	30	43	19	45	48	50	50	45	43	52	55	31	46	27	22	28	45	44	42	34	37	51	51	43
Score 6	11	8	2	11	6	8	49	18	33	26	43	27	36	8	27	18	16	15	28	52	14	29	39	62	67	72
missing	2	2	3	2	2	2	3	0	0	0	0	0	0	5	5	20	9	2	0	1	0	0	0	0	0	0

Score 1 Score 2 Score 3 Score 4 Score 5 Score 6 missing

Figure G1: Distributions of baseline total scores by sample for dimension 1

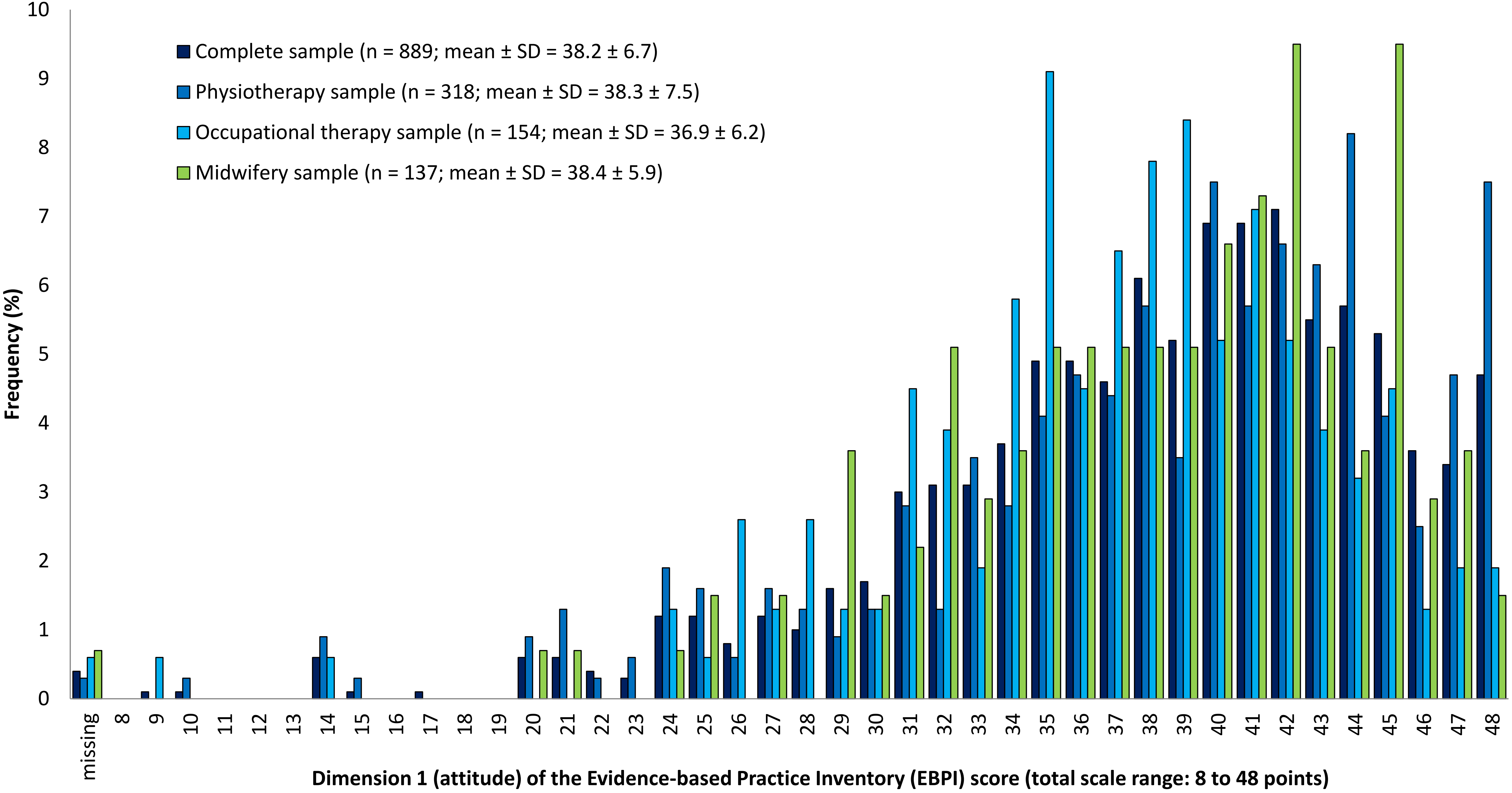

Figure G2: Distributions of baseline total scores by sample for dimension 2

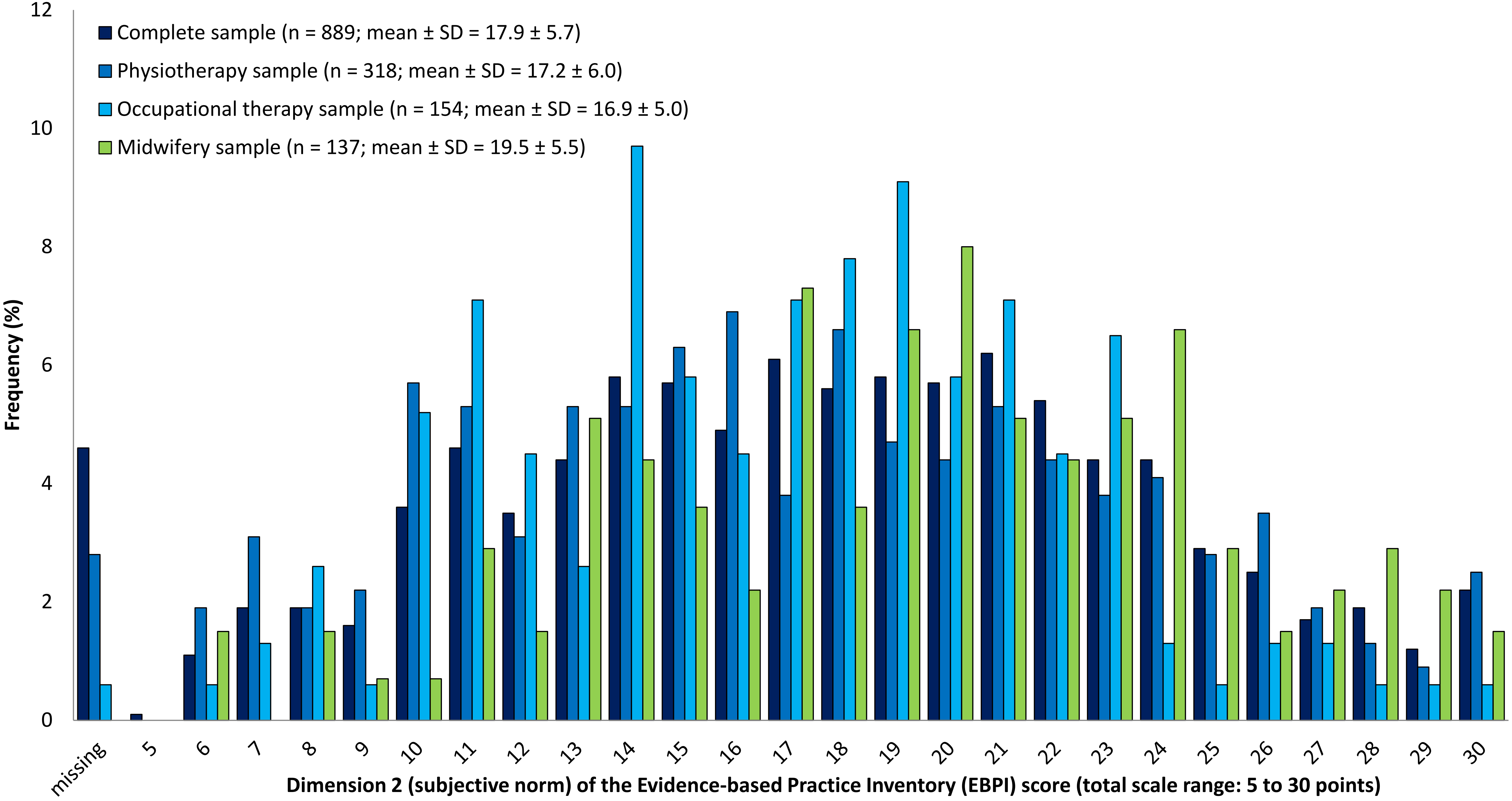

Figure G3: Distributions of baseline total scores by sample for dimension 3

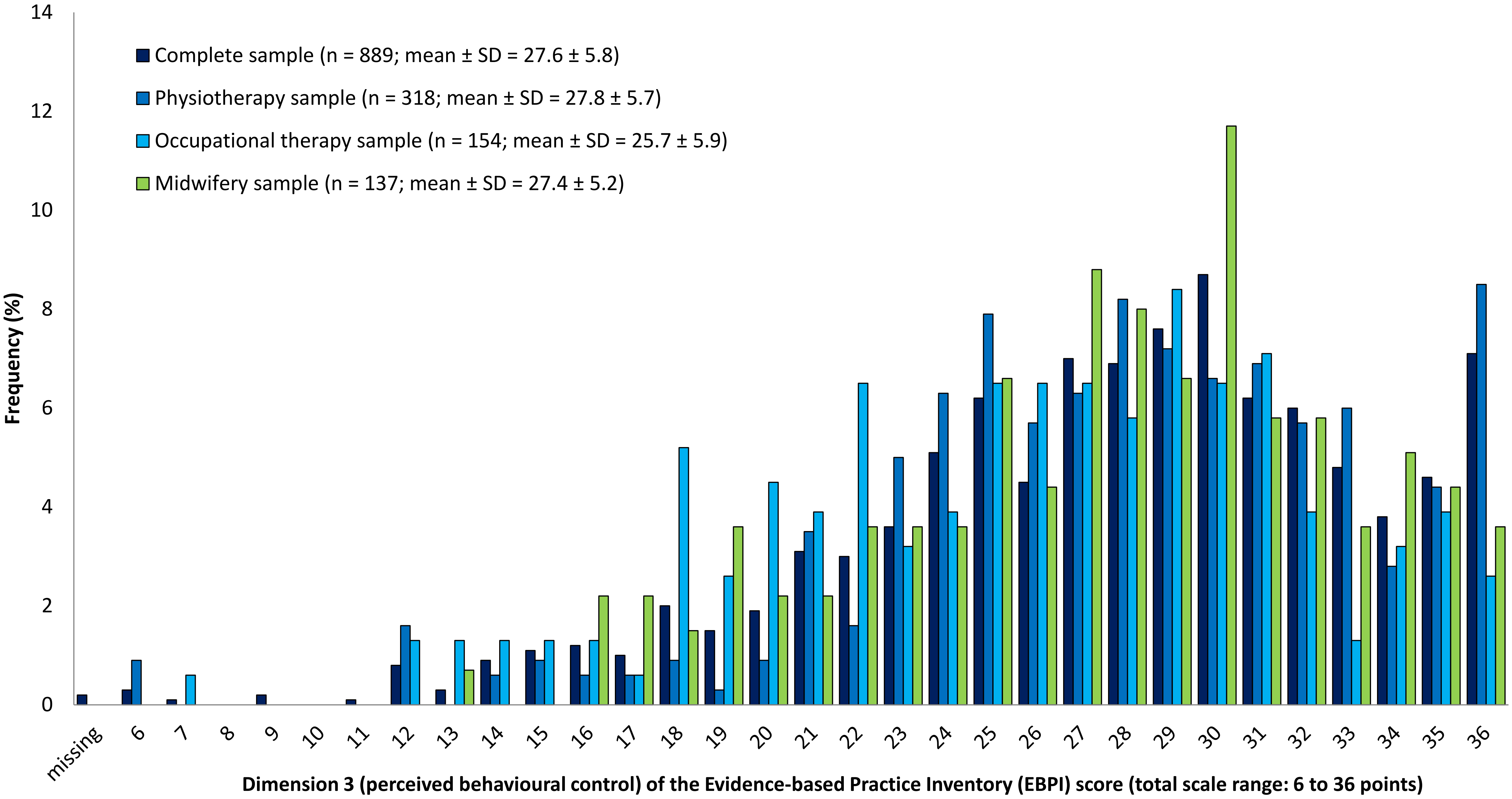

Figure G4: Distributions of baseline total scores by sample for dimension 4

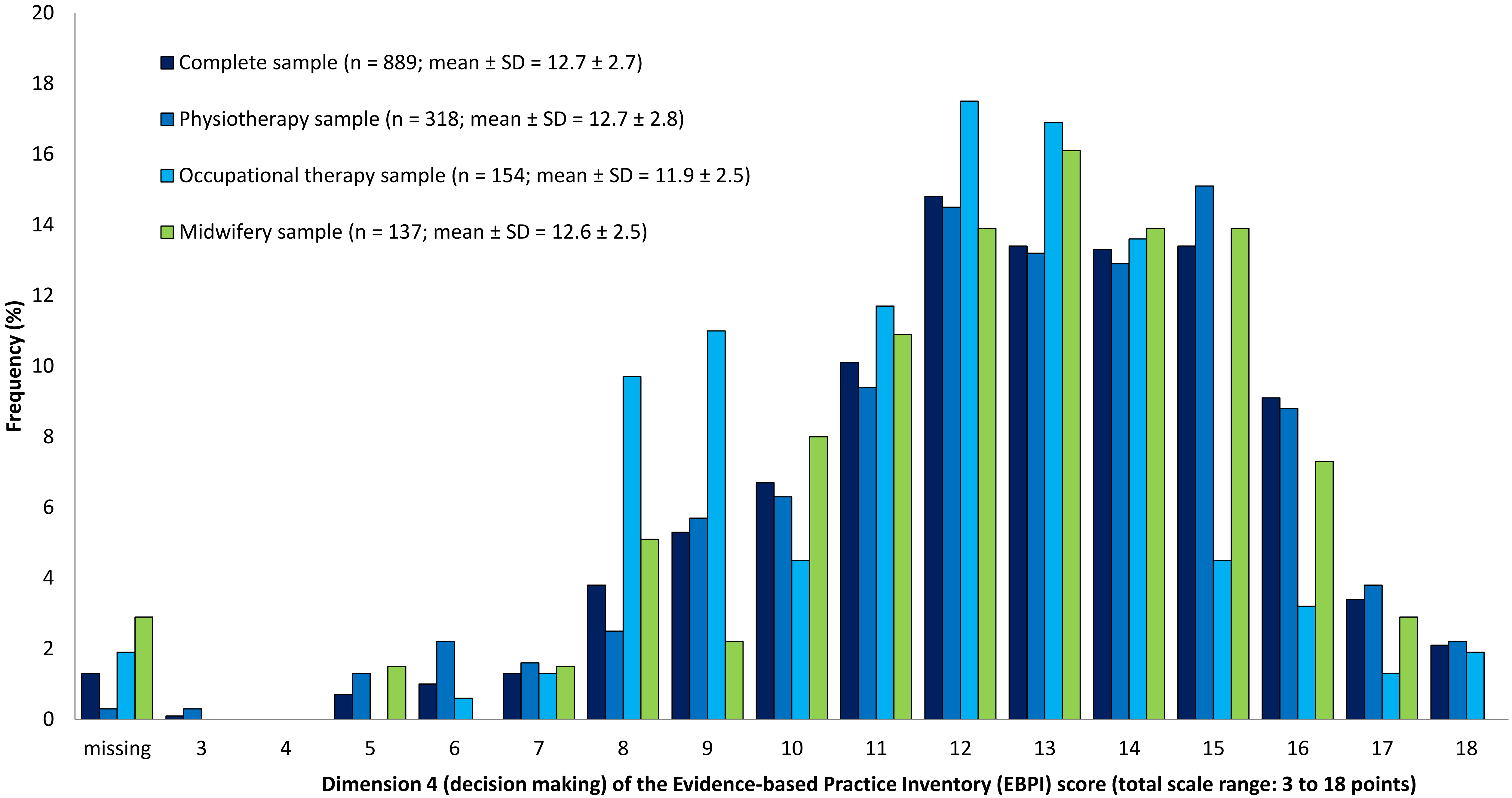

Figure G5: Distributions of baseline total scores by sample for dimension 5

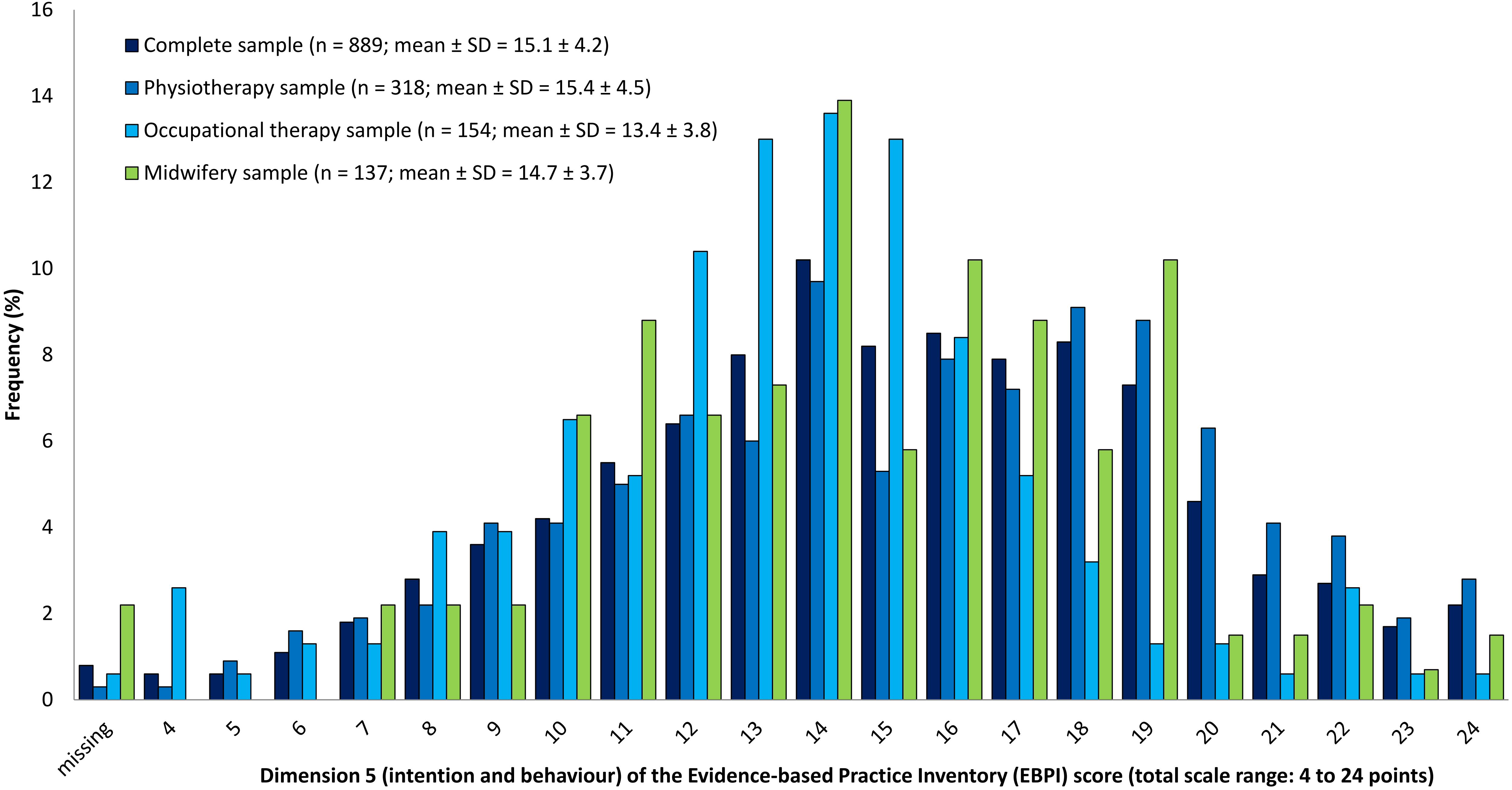

Supplement: Supplementary file 5 — Additional results (PDF 1251 kb) [file 12913_2019_4273_MOESM5_ESM.pdf]
